# Enigmatic Orthology Relationships between *Hox* Clusters of the African Butterfly Fish and Other Teleosts Following Ancient Whole-Genome Duplication

**DOI:** 10.1093/molbev/msu202

**Published:** 2014-06-27

**Authors:** Kyle J. Martin, Peter W.H. Holland

**Affiliations:** ^1^Department of Zoology, University of Oxford, Oxford, United Kingdom

**Keywords:** tetraploidy, diploidization, homeobox, WGD, *Pantodon*, teleost

## Abstract

Numerous ancient whole-genome duplications (WGD) have occurred during eukaryote evolution. In vertebrates, duplicated developmental genes and their functional divergence have had important consequences for morphological evolution. Although two vertebrate WGD events (1R/2R) occurred over 525 Ma, we have focused on the more recent 3R or TGD (teleost genome duplication) event which occurred approximately 350 Ma in a common ancestor of over 26,000 species of teleost fishes. Through a combination of whole genome and bacterial artificial chromosome clone sequencing we characterized all *Hox* gene clusters of *Pantodon buchholzi*, a member of the early branching teleost subdivision Osteoglossomorpha. We find 45 *Hox* genes organized in only five clusters indicating that *Pantodon* has suffered more *Hox* cluster loss than other known species. Despite strong evidence for homology of the five *Pantodon* clusters to the four canonical pre-TGD vertebrate clusters (one HoxA, two HoxB, one HoxC, and one HoxD), we were unable to confidently resolve 1:1 orthology relationships between four of the *Pantodon* clusters and the eight post-TGD clusters of other teleosts. Phylogenetic analysis revealed that many *Pantodon* genes segregate outside the conventional “a” and “b” post-TGD orthology groups, that extensive topological incongruence exists between genes physically linked on a single cluster, and that signal divergence causes ambivalence in assigning 1:1 orthology in concatenated *Hox* cluster analyses. Out of several possible explanations for this phenomenon we favor a model which keeps with the prevailing view of a single TGD prior to teleost radiation, but which also considers the timing of diploidization after duplication, relative to speciation events. We suggest that although the duplicated hoxa clusters diploidized prior to divergence of osteoglossomorphs, the duplicated hoxb, hoxc, and hoxd clusters concluded diploidization independently in osteoglossomorphs and other teleosts. We use the term “tetralogy” to describe the homology relationship which exists between duplicated sequences which originate through a shared WGD, but which diploidize into distinct paralogs from a common allelic pool independently in two lineages following speciation.

## Introduction

The vast majority of protein-coding genes can be organized into multigene families whose history has been shaped by duplication, loss, and speciation events (Dayhoff 1976; Nei and Rooney 2005; Demuth and Hahn 2009). When comparing genes between species, sequences are classified by categories of molecular sequence homology first introduced by Fitch (1970) over 40 years ago: Orthology (the relationship between genes present in a common ancestor and separated through a speciation event) or paralogy (the relationship between sister genes generated through a gene duplication event). Establishing correct homology relationships is a prerequisite for a powerful range of comparative genomic analyses, but requires careful phylogenetic assessment (Thornton and DeSalle 2000). It is essential to distinguish between orthologous and paralogous genes because only orthologous genes related through direct vertical descent from a common ancestor can be used to infer species-level relationships using phylogenetic tools. The type of homology between two genes may also bear on their functional evolution. The classical view is that orthologs tend to be more similar in function than paralogs, which are predisposed to diverge following duplication (Koonin 2005; Dolinski and Botstein 2007). However, the generality of this phenomenon, recently dubbed the “orthology-conjecture,” has been called into question (Studer and Robinson-Rechavi 2009; Nehrt et al. 2011; Gabaldón and Koonin 2013). With the explosion in the availability of genome sequence data, largely facilitated by the advent of “Next-Generation” sequencing technologies (Metzker 2009), the importance of accurate homology assessment has grown. This is especially true in lineages where whole-genome duplication (WGD) has occurred, resulting in the simultaneous duplication of all genes.

The high prevalence of extant polyploids suggests that WGD is both a pervasive and contemporary influence in genome evolution (Otto and Whitton 2000; Mable 2004; Otto 2007). Several clades, including vertebrates (Ohno 1970; Sidow 1996; Li et al. 2001; Putnam et al. 2008), have also experienced ancient WGDs deep in their evolutionary past, and are today considered paleopolyploids (reviewed in Van de Peer et al. 2009). Despite their polyploid ancestry, living descendants from each of these ancient WGD events are now karyotypically diploid, having undergone the remarkable and yet still largely enigmatic evolutionary transition from tetrasomic back to disomic inheritance known as diploidization. The fundamental change that characterizes diploidization is the termination of recombination between duplicated homeologous chromosomes, and their preferential bivalent pairing such that four genetically interacting tetraploid alleles at a single locus is transformed into two pairs of diploid alleles at two independent (now paralogous) loci (Wolfe 2001). Only after recombination between duplicated loci has ceased can mutations become independently fixed and duplicates diverge; we refer to this as the allele to paralog transition. Two pathways to polyploid formation are recognized: Allopolyploidy where interspecific hybridization results in the fusion of two separate parental genomes, and autopolyploidy which involves intraspecific doubling of chromosomes without hybridization. The mode of polyploidization in most paleopolyploid lineages and factors potentially affecting the rate and pattern of the subsequent diploidization process is largely unknown. Addressing these outstanding questions will be necessary to fully describe the earliest stages of duplicate gene divergence following both recent polyploidization and ancient WGDs.

**Fig. 1.**
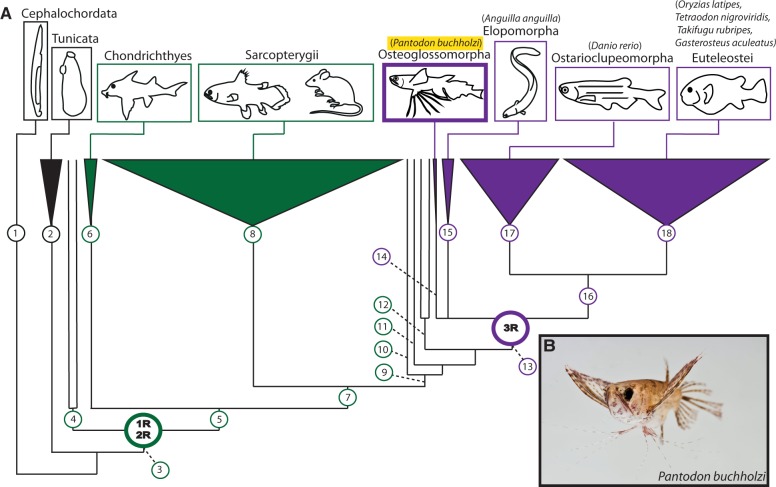
WGDs and the key phylogenetic position of *Pantodon buchholzi.* (*A*) Cladogram depicting chordate phylogeny and biodiversity with the position of WGD events shown (1R/2R, TGD/3R). Osteoglossomorpha (Clade 14) branched from other teleosts near the root of the Teleostei (Clade 13) and is therefore a useful group for deep genomic comparisons of the TGD event with teleost species whose genomes have been well characterized within the Clupeocephala (Clade 16). Other major chordate clades are also illustrated for perspective. 1: Cephalochordata, 2: Tunicata, 3: Craniata, 4: Agnatha, 5: Gnathostomata, 6: Chondrichthyes, 7: Osteichthyes, 8: Sarcopterygii, 9: Actinopterygii, 10: Polypteriformes, 11: Acipenseriformes, 12: Holostei, 13: Teleostei, 14: Osteoglossomorpha, 15: Elopomorpha, 16: Clupeocephala, 17: Ostarioclupeomorpha, 18: Euteleostei. Representative species with available genomic data used in our analyses are listed. (*B*) Adult male specimen of the African freshwater butterflyfish, *P. buchholzi*.

Within the vertebrates, two ancient WGD events are thought to have occurred in the stem lineage over 525 Ma, around the time of the divergence between the living jawless (agnathan) and jawed (gnathostome) vertebrates (fig. 1*A*; node 3); these are known as 1R/2R (first round/second round of WGD) (Holland et al. 1994; Li et al. 2001; Kuraku 2008; Putnam et al. 2008; Van de Peer et al. 2010; Smith et al. 2013). The vertebrate 1R/2R duplications were pivotal in the expansion of developmental gene families; following WGD there was a preferential retention of duplicates from this functional group, whereas most other gene families reverted to single copy (Freeling and Thomas 2006; Putnam et al. 2008; Edger and Pires 2009; Makino and McLysaght 2010). *Hox* genes encode transcription factors with many highly conserved roles during development including specification of anteroposterior positional identity across bilaterians. The expansion of the *Hox* gene repertoire by WGD in vertebrate evolution is perhaps the most widely cited example of developmental gene family expansion through duplication and has been credited with permitting a wide range of evolutionary innovations, including the establishment of fundamental components of the vertebrate body plan (Wada et al. 1999; Málaga-Trillo and Meyer 2001; Pick and Heffer 2012; Pascual-Anaya et al. 2013). The vertebrate 1R/2R events were responsible for generation of the four ancestral gnathostome *Hox* gene clusters (HoxA, HoxB, HoxC, and HoxD) from the single cluster shared with the last common ancestor of vertebrates and the cephalochordate *Branchiostoma* (Garcia-Fernàndez and Holland 1994; Putnam et al. 2008). Because of their highly conserved, colinear genomic organization, the vertebrate *Hox* gene clusters can serve as useful markers of the duplication status of entire genomes. It was partially on this basis that an additional independent WGD event was found in the teleost fish lineage (Amores et al. 1998). This event, known alternately as either 3R (third round of WGD) or the TGD (teleost genome duplication) (fig. 1*A*; node 13), occurred in the common ancestor of all approximately 26,000 species of teleost fish around 350 Ma and has been implicated by some authors in explosive speciation and morphological radiation of this group (which comprise ∼50% of all vertebrate biodiversity) (Christoffels et al. 2004; Hoegg et al. 2004; Jaillon et al. 2004; Meyer and Van de Peer 2005; Ravi and Venkatesh 2008).

Progress toward understanding the TGD event has been made by studying the relatively few teleost species with good genomic resources available, such as the laboratory models zebrafish (*Danio rerio*) and medaka (*Oryzias latipes*), the genomic models Fugu (*Takifugu rubripes*) and green spotted pufferfish (*Tetraodon nigroviridis*), and evolutionary genetic model stickleback (*Gasterosteus aculeatus*). However, all of these species belong to the single largest teleost subdivision: The Clupeocephala (fig. 1*A*, node 16), which in turn comprised the superorders Ostarioclupeomorpha (Ostariophysii + Clupeomorpha) (fig. 1*A*, node 17) and Euteleostei (fig. 1*A*, node 18). The remaining two smaller but earlier branching teleost subdivisions, Osteoglossomorpha (fig. 1*A*, node 14) and Elopomorpha (fig. 1*A*, node 15) are studied less, but are poised to extend the range of comparative genomic analyses to a time closer to the TGD. Molecular phylogenetic studies have disagreed on whether osteoglossomorphs, elopomorphs, or a clade containing both is the sister group to clupeocephans (Le et al. 1993; Inoue et al. 2001, 2003; Hurley et al. 2007; Alfaro et al. 2009; Near et al. 2012). Genomic resources for elopomorphs in the genus *Anguilla* have recently been developed (Coppe et al. 2010; Henkel, Dirks, et al. 2012), leaving the osteoglossomorphs the only major teleost subdivision without a substantial amount of genomic data available. The Osteoglossomorpha (bony-tongues) consists of approximately 220 species (<1% of all teleosts) in five families: Hiodontidae, Osteoglossidae, Notopteridae, Mormyridae, and Pantodontidae (Hilton 2003; Sullivan 2004). Pantodontidae consists of only a single species, a small (∼12 cm) tropical fish endemic to the fresh waters of Western Africa and common to the European aquarium trade since at least 1905 (White 1994): The African freshwater butterflyfish, *Pantodon buchholzi* (fig. 1*B*). The small size and widespread availability of *Pantodon*, in addition to its relatively small genome size (∼753 Mb) (Hinegardner and Rosen 1972), make it an attractive model for genomic investigations.

Seven clusters of *Hox* genes have been found in clupeocephalan teleost genomes, resulting from the duplication of the four gnathostome clusters to eight during the TGD, followed by the loss of either a single hoxc or a single hoxd cluster in the percomorph (i.e., pufferfishes, medaka, and stickleback) and ostariophysiian (i.e., zebrafish) lineages, respectively (Woltering and Durston 2006). When reconstructing gene genealogies for multiple species the resolution of two reciprocally monophyletic orthology groups, conventionally labeled by the extensions “a” and “b” in teleosts, each containing one of a pair of duplicates is considered good evidence for a gene duplication event occurring prior to a speciation event. The frequency with which this phylogenetic tree topology is observed between duplicates located on chromosomal loci sharing synteny (paralogons) forms the backbone of the evidence for the TGD. The nomenclature of the eight clupeocephalan teleost *Hox* clusters was formed on this basis (hoxa*a*/hoxa*b*, hoxb*a*/hoxb*b*, hoxc*a*/hoxc*b*, hoxd*a*/hoxd*b*). The ability to reconstruct these orthology groups with phylogenetic methods relies on the shared derived mutations unique to members of the “a” and “b” clades and it therefore stands to reason that by the time the major clupeocephalan lineages diverged, the majority of their duplicates were already segregating as distinct paralogous loci. The timing of diploidization following the TGD relative to elopomorph and osteoglossomorph divergence, however, is less clear. Recent work in an elopomorph, the European eel (*Anguilla anguilla),* revealed eight *Hox* clusters and very few individual gene losses suggesting remarkable conservation of an ancestral post-TGD *Hox* complement arrangement, but noted difficulties in assigning 1:1 orthology relationships with clupeocephalan post-TGD “a” and “b” orthology groups (Henkel, Burgerhout, et al. 2012). A polymerase chain reaction (PCR) survey of *Hox* gene fragments in an osteoglossomorph, the Goldeye (*Hiodon alosoides*), has suggested that up to eight *Hox* clusters may be also be present in this species, but relied on isolated fragments as short as 82 bp to form hypotheses of homology (Chambers et al. 2009). Other studies have also found evidence for duplication of individual genes in osteoglossomorphs, which have been interpreted as originating with the TGD (Hoegg et al. 2004; Crow et al. 2006; Chambers et al. 2009); however in many of these cases, accurate homology relationships between the osteoglossomorphs genes and other teleosts were hindered by the low phylogenetic support for inclusion of the osteoglossomorph sequence within either the “a” or “b” post-TGD orthology groups, and the absence of any potentially corroborating physical linkage data.

We have generated the first extensive genomic data set from an osteoglossomorph fish for use in deep comparative analysis of the post-TGD diploidization process. Using bacterial artificial chromosome (BAC) pyrosequencing and Illumina whole-genome sequencing methods, we obtained all *Hox* gene sequences and their complete gene cluster organization in the African butterflyfish *P. buchholzi*. Our data reveal that in *Pantodon* extensive whole *Hox* cluster loss events are coupled with high individual gene retention rates, whereas phylogenetic analysis suggests that *Pantodon* genes do not segregate as 1:1 orthologs with other teleost genes. We propose a model where following the TGD osteoglossomorphs diverged from other teleosts prior to complete genome diploidization and earlier than the fixation of the clupeocephalan teleost “a” and “b” orthology groups.

## Results

### Extensive *Hox* Cluster Loss in *Pantodon*

In order to investigate the evolutionary changes to genome structure in the immediate wake of the TGD we sought to characterize the *Hox* gene complement of the African butterflyfish *P. buchholzi*, a member of the early branching teleost subdivision Osteoglossomorpha. We hypothesized that if *Pantodon* shared the TGD with other teleosts it might have diverged before genomic changes ubiquitous to clupeocephalan teleosts arose and provide an earlier comparative genomic reference for inferring the condition of the ancestral teleost genome. We employed a three-tiered strategy involving degenerate PCR amplification, BAC clone pyrosequencing, and target-restricted assembly of Illumina whole-genome sequencing data to exhaustively screen the *Pantodon* genome for *Hox* genes.

**Fig. 2.**
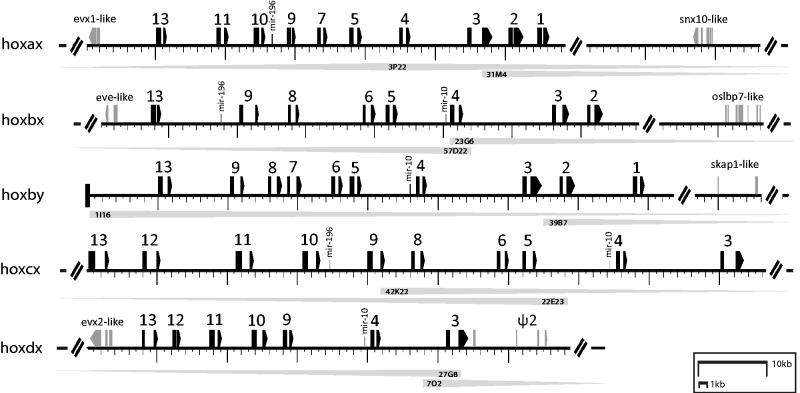
The *Hox* gene complement of *Pantodon buchholzi.* With only five clusters (hoxax, hoxbx, hoxby, hoxcx, and hoxdx) containing a total of 45 full-length protein-coding *Hox* genes, *Pantodon* possess fewer *Hox* gene clusters than any other teleosts known, but retain a similar total number of individual genes. In black, exons of predicted *Hox* genes are depicted to scale alongside conserved microRNA genes in the mir-196 and mir-10 families, flanking *Evx* family genes and the most proximate nonhomeobox flanking genes or pseudogenes (gray boxes) we could identify in the region we sequenced. The IDs of BAC clones sequenced to assemble each of these scaffolds noted underlying each cluster. The total sizes of the assembled loci, and size of the region limited by the most 5′- and 3′-*Hox* gene-coding exon for each cluster, respectively, are: hoxax (199,116/55,231 bp), hoxbx (379,217/66,149 bp), hoxby (268,422/69,795 bp), hoxcx (246,971/93,161 bp), and hoxdx (254,542/45,374 bp).

PCR amplification from *Pantodon* genomic DNA with degenerate primers targeted to homeobox sequences of *Hox* and the physically linked *Evx/Eve* gene families resulted in the amplification of 34 unique *Hox* gene and 3 *Evx/Eve* gene homeobox fragments. These fragments were pooled and used to screen a custom-made *Pantodon* approximately 6× coverage BAC library with an average clone insert size of 138 kb. Ten *Hox-*positive BACs confirmed to contain all 37 PCR fragments were pooled to build one single-end library, which was fully sequenced by 454 to an estimated average depth of 30× (supplementary table S1, Supplementary Material online). A further 48 BACs isolated in our initial screens were pooled with the previous ten *Hox*-positive BACS to construct an additional 3-kb paired-end library, which was sequenced by 454 to an additional depth of approximately 8× (supplementary table S1, Supplementary Material online). Sequencing of both libraries yielded a sufficient amount of data for de novo BAC assembly which was performed by combining both data sets using Roche’s newbler v2.6 software (supplementary table S2, Supplementary Material online). Careful manual annotation of the resulting scaffolds revealed that *P. buchholzi* possesses 45 intact full-length *Hox* gene coding sequences, interspersed with three mir-196 and four mir-10 family microRNAs in conserved positions (upstream *hox9* and *hox4* family genes, respectively), present on five separate scaffolds confirmed to encompass the ten original *Hox* BAC sequences by end sequencing (fig. 2). The five *Hox* gene clusters could be readily identified as one HoxA, two HoxB, one HoxC, and one HoxD cluster according to protein sequence similarity to the four ancestral gnathostome *Hox* gene clusters. On this basis, the five *Hox* clusters of *Pantodon* were tentatively named hoxa*x*, hoxb*x*, hoxb*y*, hoxc*x*, and hoxd*x* pending the phylogenetic evaluation of their homology with clupeocephalan teleost post-TGD “a” and “b” orthology groups.

Measured from the coding exons of the most 5′- and 3′-*hox* genes the approximate size of each *Pantodon* hox cluster is as follows: hoxax 55.2 kb, hoxbx 66.1 kb, hoxby 70 kb, hoxcx 93.2 kb, and hoxdx 45.4 kb. The size of the *Pantodon* genome has been estimated at 753 Mb (Hinegardner and Rosen 1972), intermediate between the genome sizes of zebrafish (1.5 Gb) and *Tetraodon* (342 Mb), the smallest vertebrate genome sequenced to date. The sizes of the hoxc and hoxd clusters of teleosts correlate well with the genome size and accordingly we find that the *Pantodon* hoxcx (93.2 kb) and hoxdx (45.4 kb) clusters are smaller than zebrafish hoxca (130.5 kb) and hoxda (52.6 kb) and larger than *Tetraodon* hoxca (69.3 kb) and hoxda (36 kb). The hoxcb cluster of zebrafish (72.2 kb) and the hoxdb cluster of *Tetraodon* (24.5 kb) are both reduced in size due to extensive gene loss not observed in *Pantodon* and are thus less relevant for comparison. The size of the hoxa and hoxb clusters of teleosts does not correlate as well with genome size and some *Tetraodon* clusters are larger than zebrafish. The *Pantodon* hoxax cluster (55.2 kb) is smaller than the hoxaa clusters of both zebrafish (58.2 kb) and *Tetraodon* (66.6 kb), but larger than their hoxab clusters (33.3 and 21.4 kb, respectively) which have experienced many gene losses. Both the *Pantodon* hoxbx (66.1 kb) and hoxby (70 kb) clusters are also smaller than the hoxba clusters of zebrafish (116.9 kb) and *Tetraodon* (148.8 kb) but larger than their hoxbb clusters (25.2 and 15.2 kb, respectively). Overall, the sizes of the hox clusters in *Pantodon* correlate with genome size in the case of hoxc and hoxd clusters, but not hoxa or hoxb clusters as observed in other teleosts.

Because five clusters is fewer than what has been found in any other teleost, we sought to guard against potential biases introduced by PCR amplification or BAC library construction and therefore sequenced the full genome of an individual male *Pantodon* specimen on one lane of Illumina using HiSeq2000 technology. This yielded 17.32 Gb of 100-bp paired-end (180 bp average insert size) reads corresponding to an estimated depth of 22.9× based on a genome size of 753 Mb (Hinegardner and Rosen 1972). We searched for *Hox* homeodomain sequences using the Target Restricted Assembly Method (TRAM), which allows targeted recovery and assembly of specific genomic loci from short-read sequencing data independent of whole-genome assembly (Johnson et al. 2013). The raw reads were used to build databases formatted for BLAST (Altschul et al. 1990) and HMMER (Söding 2005) searches which were queried using the collection of chordate homeodomain protein sequences contained in HomeoDB (Zhong et al. 2008) or the PFAM homeodomain hidden markov model profile (PF00046) using tBLASTn and hmmsearch algorithms, respectively, and reads were assembled using cap3 (Huang 1999). This method was specific enough to be able to independently assemble all 45 previously discovered *Hox* homeodomain-containing exon sequences at a read coverage level from 14× to 43×, and sensitive enough to recover homeodomain-encoding exons corresponding to members of all 11 currently recognized homeodomain classes. Two of these TRAM assembled exons appear to be fragments of *Hox* pseudogenes. A short fragment of a *hoxd2* gene, ψ*hoxd2x*, was subsequently found within a hoxdx BAC (7O2) sequence, whereas the homeodomain-containing exon of a second *hoxa13* gene, ψ*hoxa13y*, could not be recovered from our BAC assemblies. No sequences with similarity to an additional *hoxa13* exon 1 sequence could be identified by TRAM. We infer that this is a remnant of a deleted cluster, Δhoxay. No further *Hox* homeobox sequences could be detected. Searches for additional members of the mir-10 and mir-196 families by TRAM also failed to recover any sequences in addition to those present within the five previously characterized clusters. The results from the *Pantodon* genome searches therefore strongly corroborate the conclusion that we had characterized the complete *Hox* complement of *P. buchholzi*. Hence, according to the prevailing pan-teleost TGD model, *Pantodon* has lost three complete gene clusters (one each of HoxA, HoxC, and HoxD) following the TGD. This is the most extensive case of *Hox* cluster loss following the TGD described to date, tripling the number of whole *Hox* cluster losses previously observed in any other teleost. It therefore seems that whatever constraint might exist to limit the number of *Hox* cluster losses in other teleosts does not apply to the *Pantodon* lineage.

Interestingly, despite the unprecedented number of whole *Hox* cluster losses, *Pantodon* has retained a total *Hox* gene complement (45 genes) similar to other teleosts (e.g., 49 in zebrafish, 46 in medaka). This is a consequence of the high proportion of duplicated genes retained in the hoxbx and hoxby clusters. Two members of the *hoxb13* family are retained in *Pantodon*, where at least one copy has been lost from the genomes of all other examined teleosts. Two members of the *hoxb2*, *hoxb4*, and *hoxb9* families, which have been reduced to only one member in all clupeocephalans examined but are retained in duplicate in the elopomorph *A. anguilla* (Henkel, Burgerhout, et al. 2012), are also kept in *Pantodon*. These additional genes, notable for their absence in the genomes of zebrafish, stickleback, medaka, and pufferfish were generally assumed to have been lost simultaneously with the TGD. We infer that they were lost specifically in clupeocephans following their divergence from osteoglossomorphs and elopomorphs, indicating a significant time lag between the TGD and many duplicated gene losses. Individual *hox* gene losses have been documented even several hundred million years following the TGD (Hoegg et al. 2007). The conservation of both the duplicated *hoxb2*, *hoxb4*, *hoxb6* and *hoxb13* gene family in osteoglossomorphs suggests that the ancestral teleost hoxb clusters were far more complete than previously thought from examining only clupeocephalans.

In addition to the three whole-cluster losses, *Pantodon* has experienced several individual *Hox* gene losses since diverging from the last teleost common ancestor, including *hoxb1x*, *hoxb7x*, *hoxb10x*, *hoxb10y*, *hoxc1x*, *hoxd8x*, and *hoxd1x*. We sought to evaluate to what extent these losses were shared with clupeocephalans suggesting rapid gene loss following the TGD, or independent suggesting a time delay between TGD and major changes to the teleost *Hox* gene complement. In order to ensure the same genes are compared in *Pantodon* and other teleosts, it is necessary to establish 1:1 orthology between the *Pantodon* genes and the either the clupeocephalan counterparts belonging to either the post-TGD “a” or “b” orthology groups.

### Discordant *Hox* Gene Genealogies in *Pantodon*

We used phylogenetic methods to reconstruct unconstrained gene genealogies for each of the individual full-length *Hox* gene sequences of *P. buchholzi* to determine their orthology to clupeocephalan teleost *Hox* genes in the “a” and “b” orthology groups which formed following the TGD. The existence of more than four *Hox* gene clusters in *Pantodon* is consistent with a shared TGD model followed by the independent loss of three whole clusters in the *Pantodon* lineage. We therefore initially hypothesized that *Hox* genes in *Pantodon* would segregate with the canonical “a” or “b” post-TGD orthology groups recovered in clupeocephalan teleosts, whereas an unduplicated or independently duplicated sequence in *Pantodon* would segregate strictly outside of these clades. Because of their early divergence from other teleosts, osteoglossomorph sequences would by default be expected to segregate at the root of any teleost gene tree, potentially causing problems due to hidden paralogy where clupeocephalan teleosts retained only a single duplicate (Kuraku 2010). We therefore limited analyses to 26 orthology-informative *Pantodon* sequences (comprising 22 gene families) for which at least one clupeocephalan teleost with publicly available complete *Hox* gene complements retained both “a” and “b” paralogs. Two different phylogenetic reconstruction methods, maximum-likelihood (ML) and Bayesian Inference (BI), were used to evaluate the robustness of each gene genealogy and in order to minimize the potential impact of saturation due to the large amount of time since the TGD only amino acid sequences were used.

**Fig. 3.**
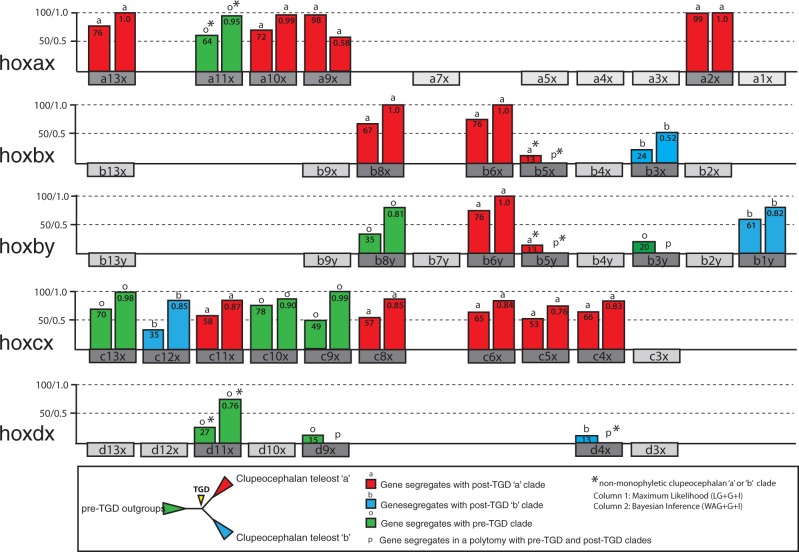
The *Hox* genes of *Pantodon buchholzi* do not reliably segregate with the post-TGD “a” and “b” orthology groups of other teleosts in individual unconstrained vertebrate *Hox* gene trees. Phylogenetic trees were computed using ML and Bayesian methods with the *Hox* gene-coding sequences of *Pantodon*, other teleosts (eel, zebrafish, salmon, medaka, stickleback, *Tetraodon*, *Takifugu*, and *Astatotilapia*), sarcopterygians (coelacanth, *Xenopus*, *Anolis*, human, and mouse), and elephant shark. Support values corresponding to clades containing *Pantodon* sequences are plotted as two columns above each individual orthology-informative gene in the Hox cluster schematic. The height of each column corresponds to either the bootstrap support value (left column) or the posterior probability (right column). The column color corresponds to the clade containing the *Pantodon* sequence. We observe that across a single cluster, *Hox* genes where the *Pantodon* sequence clusters best with clupeocephalan post-TGD “a” orthologs (red), are interleaved with those which cluster best with the post-TGD “b” orthologs (blue), or outside the combined post-TGD “a” and “b” clades with nonteleost outgroups (green). Polytomies between the *Pantodon* sequence, the pre-TGD outgroups, and post-TGD “a” and “b” clades occurring in the Bayesian trees are indicated with a letter “p.” Under a classic pan-teleost TGD model we would expect all *Pantodon* genes on each *Hox* cluster to segregate with either the “a” or the “b” orthology groups rather than a mixture, and no sequences which segregate best as an outgroup to the TGD node.

Contrary to our initial expectation, we found that many individual *Pantodon* gene tree topologies appeared to be inconsistent with our expectations under a shared TGD event (fig. 3; supplementary figs S1 and S2, Supplementary Material online). Rather than a single 1:1 orthology for each *Pantodon* gene with clupeocephalan genes belonging to either the TGD “a” or “b” orthology groups, several *Pantodon* genes segregated as an outgroup to, or in a polytomy with these clades. Furthermore, although we would expect genes which segregate within the “a” orthology group to be physically clustered together on a separate chromosome from those which segregate with the “b” orthology group, as found in other teleosts, we found that this pattern is disrupted in *Pantodon*. Even where strong phylogenetic support for 1:1 orthology of an individual *Pantodon* gene with members of either the clupeocephalan post-TGD “a” or “b” clades existed, adjacent genes yielded incongruent topologies such that within a single *Pantodon* cluster, genes grouping with the “a” and “b” orthology groups were interleaved, along with those which resolve best outside the combined TGD “a” and “b” clades entirely.

**Fig. 4.**
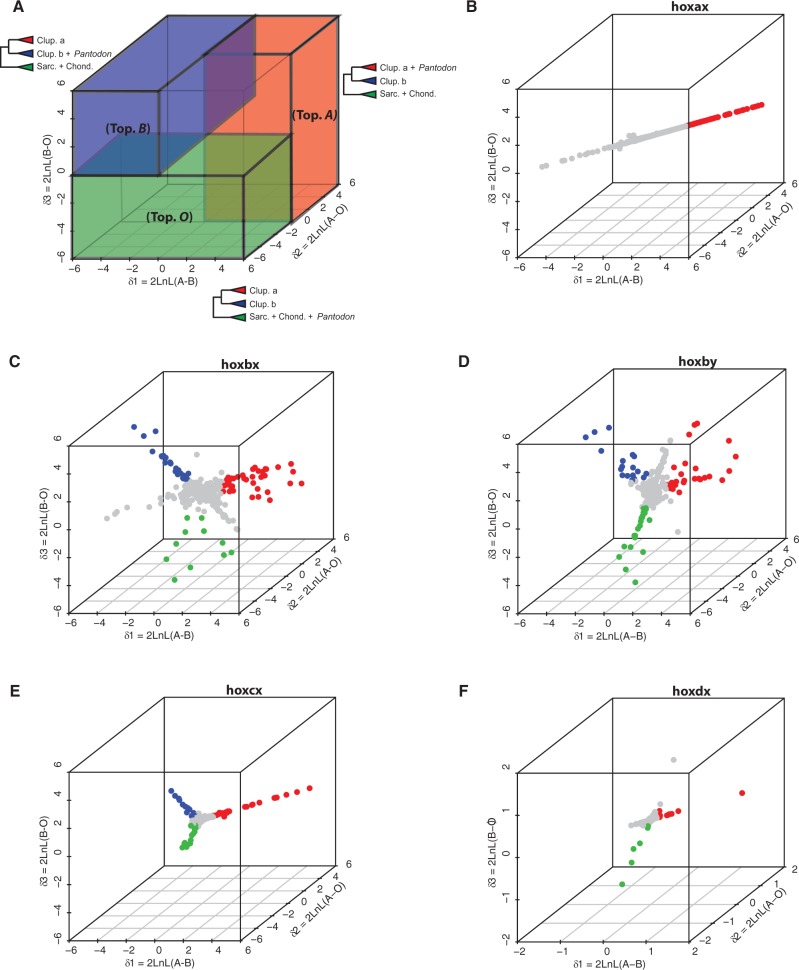
3D-SLRP using vertebrate whole Hox cluster concatenations reveals strong cluster-wide conflict between individual sites supporting each of three alternative hypotheses of *Pantodon* Hox cluster homology. Three different constrained tree topologies representing different hypotheses of *Pantodon* Hox cluster homology were compared. Topology A: ((post-TGD “a” + *Pantodon*)(post-TGD “b”))(pre-TGD outgroups), topology B:((post-TGD “a”)(post-TGD “b” + *Pantodon*))(pre-TGD outgroups), and topology O:((post-TGD “a”)(post-TGD “b”))(pre-TGD outgroups + *Pantodon*) were each compared in a pairwise fashion under a ML framework to model the support for each hypothesis of homology across each site in whole Hox cluster-concatenated alignments. (*A*) Schematized outline of a site-likelihood ratio plot showing regions in the graph which support each topology. Actual site-likelihood ratio plots for each *Pantodon* cluster are shown for hoxax (*B*), hoxbx (*C*), hoxby (*D*), hoxcx (*E*), and hoxdx (*F*). Each axis plots the site-wise likelihood ratio difference between one pair of competing topologies. The *x* axis plots the likelihood ratio between topology A and topology B (δ1), the *y* axis plots the likelihood ratio between topology A and topology O (δ2), and on the *z* axis the likelihood ratio between topology B and topology O (δ3) is plotted. Each point represents a single amino acid site. Sites are colored if the absolute magnitude of the corresponding site-likelihood ratio is more than 2 SD greater than the mean. Sites which support topology A are colored in red, sites which support topology B are blue, whereas sites which support topology O are green. Except for hoxax, which only contains sites which support topology A, there is conflicting phylogenetic signal in the *Hox* clusters of *Pantodon* which prevents unambiguous assignment to either the clupeocephalan teleost post-TGD “a” or “b” orthology groups.

Examining the five orthology-informative genes on the *Pantodon* hoxax cluster revealed that four genes segregate with high support and good concordance between both ML and BI methods (bootstrap/posterior probability) with the post-TGD “a” orthology group: *hoxa13x* (76/1.0)*, hoxa10x* (72/0.98)*, hoxa9x* (98/0.58), and *hoxa2x* (99/1.0). Only *hoxa11x* segregated as an outgroup (64/0.82) to both the post-TGD “a” and “b” clades with the caveat that zebrafish *hoxa11b* broke the expected monophyly of the clupeocephalan “b” clade. We used the likelihood-based approximately unbiased (AU) test for alternative tree topologies (Shimodaira 2002) to evaluate three constrained topologies in which the *Pantodon* sequences were forcibly orthologized with either the clupeocephalan “a” (topology A) or “b” (topology B) post-TGD orthology groups or as an outgroup to the combined TGD “a” and “b” clade (topology O) (see fig. 4). All three constrained topologies enforced the respective monophyly of the post-TGD “a” and “b” orthology groups of clupeocephalans as well as the monophyly of the sequences used as unduplicated outgroups. Using this test, we were able to confidently (*P* < 0.05) rule out alternatives to orthology with the post-TGD “a” group (topology A) for three of five genes on the hoxax cluster: *hoxa13x, hoxa9x*, and *hoxa2x.* We were also able to rule out orthology of *hoxa11x* with the post-TGD “b” group (topology B), but not the possibility of an outgroup relationship (topology O). Only for *hoxa10x* was this test not able to rule out any of our three alternative hypotheses. Taking both the unconstrained phylogenies and constrained topology tests into account, we suggest that the hoxax cluster is a true 1:1 ortholog of the clupeocephalan hoxaa clusters. This is in line with our initial expectations for simple 1:1 orthology between *Pantodon* sequences and other teleosts due to a shared TGD event. The results from the remaining *Hox* genes, however, did not meet with our expectations under the prevailing model of the TGD.

Out of four orthology informative genes on the *Pantodon* hoxbx cluster two genes, *hoxb8x* (67/1.0) and *hoxb6x* (76/1.0) segregated with the post-TGD “a” orthology group, whereas *hoxb5x* grouped either weakly within a subset of clupeocephalan sequences belonging an atypically paraphyletic “a” clade (ML) or formed a polytomy with “a” and “b” clades (BI), and *hoxb3x* segregated best in an contradictory manner with the post-TGD “b” orthology group with both tree reconstruction methods, albeit with low support (24/0.52). Most alternative constrained topologies evaluated by the AU test were not significantly different from each other and only topology O for *hoxb8x* could be confidently rejected (*P* < 0.05).

Considering the hoxby cluster, with five informative genes, *hoxb6y* grouped reliably with the post-TGD “a” orthology group according to both methods (76/1.0), whereas both *hoxb8y* and *hoxb3y* segregated either as outgroups to or formed polytomies with the inclusive TGD “a” and “b” clades, and *hoxb1y* segregated with the post-TGD “b” orthology group (81/1.0). Curiously, *hoxb5y* grouped closely with *hoxb5x* among a clade of clupeocephalan sequences belonging an atypically paraphyletic clade comprising the nonpercomorph members of the post-TGD “a” orthology group (ML), or formed a polytomy with both “a” and “b” clades (BI). We also observe that both *hoxb6x* and *hoxb6y* also segregate best within the same clade comprising members of the clupeocephalan “a” orthology group. These irregularities are contrary to the default expectation under a shared TGD event which predicts that each *Pantodon* duplicate would belong to a single one of either the “a” or “b” orthology groups rather than both segregating together with only one. When we tested the statistical support for alternative constrained topologies we were able to eliminate topology O for *hoxb8x*, topology A for *hoxb8y*, and both topology A and O for *hoxb3y* which had appeared to segregate best as an outgroup to the inclusive TGD “a” and “b” clades in our unconstrained ML trees.

The hoxcx cluster has nine informative genes, five of which group with the post-TGD “a” orthology group: *hoxc11x* (58/0.87)*, hoxc8x* (57/0.85), *hoxc6x* (65/0.84), *hoxc5x* (53/0.76), and *hoxc4x* (66/0.83). Three genes segregate as outgroups to the TGD “a” and “b” clades: *hoxc13x* (70/0.98), *hoxc10x* (78/0.90), and *hoxc9x* (49/0.99). Finally one gene segregated best with the post-TGD “b” clade: *hoxc12x* (35/0.85). The AU test was unable to rule out more than one of the three competing topologies for any gene of the hoxcx cluster, eliminating only topology B in the case of *hoxc10x, hoxc9x*, and *hoxc5x.*

Finally, within the hoxdx cluster, none of the three informative genes present segregated with high support in either the TGD “a” or “b” orthology groups. Although *hoxd4x* grouped best with clupeocephalan “b” genes in our ML analysis, bootstrap support was so exceedingly low (13) as to be considered unsupported. Of the other two informative genes, *hoxd11x* segregated as an outgroup to the TGD clade (27/0.76), whereas *hoxd9x* segregated either in an outgroup or in a polytomy inclusive of both TGD “a” and “b” clades. When the competing constrained topologies were evaluated through the AU test, none of the three topologies for any genes in the hoxdx could be rejected at a confidence level of *P* < 0.05.

The topological incongruences we observed between the individual *Pantodon* gene trees could be strictly interpreted as a contradiction of the prevailing hypothesis of a TGD event inclusive of osteoglossomorphs. However, both the presence of a duplicated cluster (hoxbx and hoxby) and the strong support for the inclusion of multiple individual *Pantodon* genes within the clupeocephalan teleost post-TGD “a” or “b” orthology groups (particularly those of the hoxax cluster) support the idea of a TGD event inclusive of *Pantodon*.

### Selection Is Not Sufficient to Explain Incongruent *Hox* Gene Genealogies

We sought an answer to the question of why the hoxbx, hoxby, hoxcx, and hoxdx clusters behaved atypically compared with the hoxax cluster in our phylogenetic analyses. It has been proposed that diversifying selection occurred in the immediate wake of the TGD between some gene duplicates, driving them apart, but not others (Crow et al. 2006). If this occurred between the genes in the duplicated hoxa clusters, but not in the hoxb, hoxc, or hoxd clusters this might cause stronger signal to accumulate between these duplicates and facilitate faithful reconstruction of their orthology. We tested for the presence of diversifying selection acting on the immediate post-TGD branches of each of the 22 orthology-informative *Hox* gene trees to compare the selective pressures on each of the duplicated *Hox* clusters under the assumption of an osteoglossomorph-inclusive TGD. Using constrained trees which enforced monophyly of the post-TGD “a” and “b” orthology groups of clupeocephalans but allowed the *Pantodon* sequence freedom, the MG94 codon substitution model (Muse and Gaut 1994) was used to estimate d*N*, d*S*, and ω ( = d*N*/d*S*) independently for each branch of the tree using a local model fit with the HyPhy software package (Pond et al. 2005). We found no evidence for different selective pressures acting immediately after the TGD between the hoxa gene trees and the hoxb, hoxc, or hoxd trees (supplementary table S3, Supplementary Material online). Only two branches arising directly from the TGD node (hoxa9a/hoxa9x and hoxc4b) had ω > 1 (ω = 0.183/0.128 = 1.43 and ω = 0.014/0.002 = 6.68, respectively), consistent with positive selection. There were 15 additional proximate post-TGD branches in gene trees belonging to each of the hoxa, hoxb, hoxc, and hoxd clusters which returned infinite estimates for ω because d*S* was estimated at 0 under the topological constraints we imposed. In each of these cases, d*N* was estimated at < 0.02 making any inference of positive selection based on these results weak. Previous studies have also found evidence for constraint in the synonymous substitution rate of *Hox* genes which may inflate estimates of positive selection (Lin et al. 2008). Even if we were to take these results as acceptable evidence for positive selection, the prevalence of selection after the TGD node is no higher in the duplicated hoxa than in the hoxb, hoxc, or hoxd clusters.

Finally, because diversifying selection may be both episodic and affect only a small proportion of sites in an alignment, we tested for selection acting on only a small number of sites which might otherwise be lost in an overwhelmingly constrained sequence using the branch-site REL method implemented in HyPhy (Kosakovsky Pond et al. 2011). This method allows for three independent categories of ω estimates along each branch to accommodate site-wise variation in selective pressures, and does not require constraining the rest of the tree to a single d*N* and d*S* rate while testing for selection along one branch. Only one additional statistically significant instance of positive selection (Bonferroni corrected *P* < 0.05) could be found at the root of a post-TGD clade: A small proportion of sites (*q*^+ ^= 4.44%) under positive selection could be found in the *hoxa2a/x* branch. We therefore believe that differences in selective pressure alone are therefore insufficient to explain the incongruent gene tree topologies in the *Pantodon* clusters.

### Heterogeneous Phylogenetic Signal within Concatenated *Hox* Clusters

We sought to explore additional explanations for the failure for the *Hox* gene genealogies of *Pantodon* to match the predictions of a shared TGD event. Given the relatively short sequence length of individual *Hox* genes and their high levels of conservation, we asked whether the incongruent topologies we recovered were the result of stochastic variation due to low signal intensity. The concatenation method has been useful in resolving phylogenetic problems where weak signal due to limited data has obscured underlying evolutionary relationships (Rokas et al. 2003). We built concatenated superalignments comprising all full-length protein-coding genes from each physically linked *Hox* cluster in *Pantodon* and a collection of other vertebrates. For each *Hox* cluster superalignment, we explicitly tested the collective orthology of the sequences within the *Pantodon* clusters to those of other teleosts by constructing trees for three competing constrained topologies and testing the statistical support for each using the AU test (Shimodaira 2002). As for individual genes, we constrained the *Pantodon* clusters explicitly within either the post-TGD “a” cluster (topology A) or “b” cluster (topology B) as would be expected under the canonical shared TGD, or outside the inclusive TGD “a” and “b” clades (topology O). The results of this test (summarized in supplementary table S4, Supplementary Material online) indicate that globally the *Pantodon* hoxax cluster is a 1:1 ortholog of the post-TGD “a” clusters of clupeocephalan teleosts, as expected for a shared TGD. Topologies B (*P* = 2 × 10^−^^4^) and O (*P* = 2 × 10^−^^4^) could be confidently rejected consistent with our results from individual gene genealogies. However, even when the signal of all informative *Hox* genes on a single cluster was concatenated, the results with the *Pantodon* hoxbx, hoxby, hoxcx, and hoxdx clusters still broke our assumptions of 1:1 orthology with other teleosts, as in our individual gene genealogies. For the *Pantodon* hoxby cluster and the hoxdx cluster none of the three competing topologies could be rejected, whereas for the hoxbx and the hoxcx clusters only topology O could be rejected (*P* = 0.02 and *P* = 0.05, respectively).

We employed a novel graphical method to visualize signal intensity and direction under three competing constrained tree topologies: 3D site-likelihood ratio plotting (3D-SLRP). We plotted the per-site likelihood ratios {δ_s_ = 2 ln[(Site-likelihood Topology 1) − (Site-likelihood Topology 2)]} which were calculated during the AU test between each pair of competing constrained topologies (A vs. B, A vs. O, and O vs. B) to obtain a 3D plot of the site-wise distribution of support for each of our three test topologies (fig. 4). Except for *Pantodon* hoxax, which shows a clear majority of sites uniquely supporting topology A (fig. 4*B*, red dots), the plots for the four remaining clusters show signal trifurcations in site-wise support for topologies A (red dots), B (blue dots), and O (green dots) (fig. 4*C*–*F*). Although the intensity of the trifurcating signals in hoxbx, hoxby, and hoxcx is as strong as the unidirectional signal in hoxax, in the case of hoxdx the signal is slightly weaker and this may have contributed to the difficulty in assigning orthology to this cluster. This heterogeneity in the signal for the full *Hox* cluster concatenations indicates that the failure of at least the *Pantodon* hoxbx, hoxby, hoxcx cluster concatenations to form clear 1:1 orthologous relationships with either the “a” or “b” post-TGD orthology groups in our AU tests is the result of conflicting signal between different sites in the *Pantodon* clusters, rather than an absence of signal. This outcome corroborates the results from the individual gene genealogies and supports the idea that the phylogenetic histories of individual sites/genes within the *Hox* clusters of *P. buchholzi* are heterogeneous.

### Ancient Conserved Noncoding Element Retention Patterns in *Pantodon* Hox Clusters

Identifying conserved noncoding elements (CNEs) is a useful method of identifying functional genomic elements, and many vertebrate CNEs found near developmental genes have been shown to play a role in gene regulation (Woolfe et al. 2005; Pennacchio et al. 2006). Genome-wide, ancient gnathostome CNEs (conserved between chondrichthyans and osteichthyans) appear to be evolving more quickly in teleosts than in other vertebrate lineages (Venkatesh et al. 2006; Lee et al. 2010). Earlier comparison of *Takifugu* and human Hox clusters with elephant shark *Callorhinchus milii* revealed that the duplicated *Takifugu* clusters retain fewer ancient CNEs than human and that all of the *Takifugu* post-TGD “b” clusters are depleted of ancient CNEs compared with the “a” clusters (Ravi et al. 2009). We performed VISTA alignments using the SLAGAN algorithm to identify ancient CNEs between elephant shark and the Hox clusters of *Pantodon* and five other teleosts (zebrafish, medaka, stickleback, *Takifugu*, and *Tetraodon*) (supplementary fig. S3 and table S5, Supplementary Material online). Our analysis reveals that although the post-TGD “b” clusters of all five clupeocephalan teleosts show a distinctive asymmetry in the retention of CNEs, the duplicated *Pantodon* hoxb clusters retain nearly the same number of CNEs (hoxbx:14, hoxby:15) (supplementary fig. S4*B*, Supplementary Material online). In clupeocephalans, the hoxbb cluster appears to have diverged more than the hoxba cluster having lost more CNEs (e.g., zebrafish hoxba: 26; hoxbb: 4, *Tetraodon* hoxba: 8, hoxbb: 3). The gene content of the clupeocephalan hoxbb clusters is also significantly lower than hoxba (e.g., zebrafish hoxba: 11; hoxbb: 4, *Tetraodon* hoxba: 9; hoxbb: 4). The symmetric pattern of CNE conservation in both *Pantodon* hoxb clusters is consistent with the higher degree of *Hox* gene retention (hoxbx: 8; hoxby: 10) and suggests a functional role for these CNEs, potentially in the regulation of *Hox *gene duplicates conserved in *Pantodon* but lost in other teleosts. This also suggests that the rapid structural evolution of the clupeocephalan hoxbb cluster only got underway after the speciation event separating *Pantodon* and other teleosts*.*

Ancient CNE retention patterns in the *Pantodon* hoxax, hoxcx and hoxdx clusters are more similar to clupeocephalan hoxaa, hoxca and hoxda clusters, respectively, than to hoxab, hoxcb or hoxdb which like hoxbb have experienced extensive *Hox* gene and CNE loss (supplementary fig. S4*A*, *C*, and *D*, Supplementary Material online). However, without corresponding duplicated clusters in *Pantodon* to compare with it is impossible to conclude whether the rapid structural evolution of the clupeocephalan hoxab, hoxcb and hoxdb clusters occurred before or after separation of the lineage leading to *Pantodon*. If asymmetric structural evolution only occurred in the clupeocephalan lineage after this speciation, as seems likely in the case of hoxbb, then presence or absence of CNEs is not phylogenetically informative.

## Discussion

We suggest a biological explanation for the unusual pattern of molecular evolution we observe that fits all of our data and is in line with our current understanding of genome diploidization dynamics in polyploids, but which has been largely overlooked in the study of ancient genome duplications. We propose that the four alleles of the duplicated hoxb, hoxc, and hoxd clusters did not complete diploidization into two pairs of alleles on separate paralogous gene clusters until after the very early divergence of osteoglossomorphs and clupeocephalans. In contrast, after the TGD the hoxa clusters resolved faster, prior to divergence of these lineages. This model implies that the last teleost common ancestor was a pseudotetraploid, like many present-day species which more recently underwent WGD, and had a composite genome consisting of both diploidized and tetraploid segments. Prolonged segregation as four alleles in a pseudotetraploid lineage would potentially subject the duplicated hoxb, hoxc, and hoxd clusters to concerted evolution between duplicated loci, violating the assumptions of immediate genetic independence and simple 1:1 orthology of all teleost duplicates following the TGD.

One alternative model is that osteoglossomorphs experienced an independent WGD around the same time as the TGD, after diverging from other teleosts. Recently, an independent WGD event has been described in the American paddlefish (*Polyodon spathula*), a nonteleost actinopterygian fish, underlining the potential for undiscovered independent WGDs in other vertebrate lineages (Crow et al. 2012). However, this model does not explain the strong phylogenetic affinity for *Pantodon* hoxax with clupeocephalan hoxaa and, depending on the relative position of osteoglossomorphs and elopomorphs in the teleost tree, it might require yet another independent WGD in the elopomorph lineage. Likewise, independent duplication of the hoxb cluster in *Pantodon* is unlikely to have occurred as this would implicate additional independent duplications of the other clusters in the *Hiodon* lineage, which retains eight Hox clusters (Chambers et al. 2009), and does not fit our phylogenetic results. Another model might be that although osteoglossomorphs shared the TGD, the unprecedented loss of three whole Hox clusters in *Pantodon* constrained the evolution of the remaining clusters causing them to retain their similarity to their pre-TGD counterparts. While plausible, under this model we would expect that singular clusters in *Pantodon* (hoxax, hoxcx, hoxdx) would be more constrained and more difficult to homologize than clusters retained in duplicate (hoxbx/hoxby). However, what we observe is that the singular *Pantodon* hoxax cluster segregates well with the hoxaa clusters of other teleosts (which have never lost either of their hoxaa or hoxab clusters). Conversely, the pair of *Pantodon* hoxbx/hoxby clusters does not form clear 1:1 orthologies with other teleosts. Additionally, in clupeocephalan *Hox* genes we do not note any consistent difference in the ability of orphaned duplicates compared with replete pairs of genes in their ability to segregate in a single post-TGD orthology group. Finally, although our analyses suggest that selection on protein-coding genes was not significantly different between Hox clusters in the immediate wake of the TGD, and that signal trifurcation rather than absence was responsible for ambiguity in assigning 1:1 orthologies to the most of the clusters, it remains possible that forces operating at some as yet unidentified level are responsible for the phenomenon we observe here. The possible effect of long-branch attraction which can result in the erroneous grouping of quickly evolving branches in a phylogenetic tree by misinterpreting homoplastic characters as homologous changes should not be discounted. However, the generally high sequence conservation of *Pantodon* genes evident from selection analyses (supplementary table S3, Supplementary Material online) and the absence of strong statistical support for grouping the *Pantodon* sequences with quickly evolving branches such as the percomorph *hoxd4b*, *hoxd9b*, and *hoxd11b* (supplementary figs. S1*T–V* and S2*T–V*, Supplementary Material online) suggest that long-branch attraction is not a prominent factor in this case. The most plausible explanation we can give at this time remains the segmental and independent diploidization of the *Pantodon* genome following a shared TGD.

### Considering Diploidization Following the TGD

The finding that *P. buchholzi* has five *Hox* clusters including two HoxB loci is in agreement with the prevailing view that the TGD predates the divergence of the Osteoglossomorpha and hence is shared by all teleosts (Hoegg et al. 2004; Crow et al. 2006; Hurley et al. 2007). Based on observations in other teleosts, it is thought that the last teleost common ancestor possessed eight *Hox* clusters immediately after the TGD. This implies that three *Hox* clusters were lost in the *Pantodon* lineage (one HoxA, one HoxC, and one HoxD cluster). Teleost *Hox* cluster evolution appears to be significantly more dynamic than other gnathostome vertebrates which almost invariably retain the four ancestral *Hox* clusters with relatively few independent gene losses in different lineages (Liang et al. 2011). The only known exception to this stability are the elasmobranchs *Scyliorhinus canicula* and *Leucoraja erinacea* which appear to have lost their HoxC cluster after diverging from chimaerids >400 Ma (Ravi et al. 2009; Oulion et al. 2010; King et al. 2011). However, even among teleosts which have seen the zebrafish, and salmon lineages independently lose their hoxdb clusters (Woltering and Durston 2006; Mungpakdee et al. 2008), and percomorphs including medaka, pufferfish, and cichlids lose their hoxcb clusters (Amores et al. 2004; Tümpel et al. 2006; Hoegg et al. 2007), the loss of the hoxay, hoxcy, and hoxdy clusters in *Pantodon* stands out as the most extensive case *Hox* cluster loss currently known.

The presence of the duplicated HoxB cluster alone, however, is not strictly sufficient to conclude that *Pantodon* shared the TGD and lost three clusters. Our phylogenetic analyses revealed that most individual genes within the *Pantodon* hoxax cluster, and the whole cluster considered together in a concatenation of its component genes, groups with very good support with the hoxaa clusters of clupeocephalan teleosts exactly as one would expect if *Pantodon* shared the TGD with clupeocephalans and subsequently lost the hoxay cluster. This also implies that by the time *Pantodon* diverged from other teleosts, the duplicated HoxA clusters were already segregating as fully diploidized paralogous loci. However, the interleaving phylogenetic affinities of individual *Hox* genes within the *Pantodon* hoxbx, hoxby, hoxcx, and hoxdx clusters for incongruent topologies, considered together with the trifurcation in the support of individual sites within these clusters for the post-TGD “a” or “b” orthology groups, or outgroup topologies, contradict the predictions for a shared TGD event if we assume that all *Hox* clusters concluded diploidization simultaneously prior to the radiation of major teleost subdivisions.

**Fig. 5.**
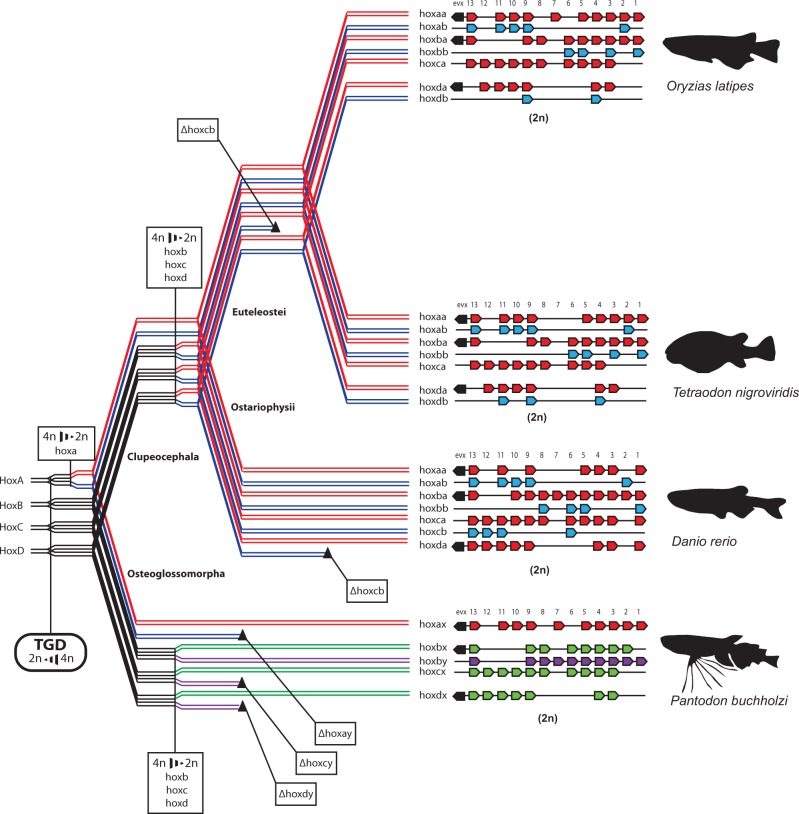
The evolution of teleost *Hox* gene clusters following the TGD outlining the relative timing of diploidization events and the speciation of major teleost subdivisions. This model of Hox cluster evolution in teleosts illustrates the independent diploidization of *Hox* clusters following the TGD and the relative timing of cluster diploidization and speciation events. Each line in the phylogram represents an allele and the separation of pairs of lines accompanied with a change in color represents the completion of the diploidization of this locus. In this model, the duplicated hoxa clusters diploidize first, before the last common ancestor of all teleosts. The remaining clusters diploidize later, and independently in Clupeocephala and Osteoglossomorpha. Whole *Hox* cluster losses (black triangles) are also mapped highlighting the massive Hox cluster losses in the *Pantodon* lineage.

These apparently paradoxical findings can be reconciled by postulating a protracted pseudotetraploid period in teleost evolution, following the ancient TGD polyploidization event and preceding the establishment of the fully diploid (paleopolyploid) karyotype of modern teleosts. If *Pantodon* diverged from other teleosts during this pseudotetraploid period, prior to the conclusion of the diploidization process, it is likely that it shared the diploidization of some genomic loci prior to speciation, but not others. The effects of homeologous recombination and gene conversion between the highly similar tetraploid alleles of duplicated but undiploidized loci, as well as the potential for independent sorting of alleles during the speciation process, could have led to the divergent phylogenetic pattern which we observe if osteoglossomorphs diverged early enough that diploidization was concluded independently from other teleosts. If these genetic homogenizing forces were strong enough they would have the effect of slowing down or even stopping the molecular clock, preventing differences between duplicates from becoming fixed and effectively delaying their divergence until after diploidization had concluded. If this continued until after the deep divergence between osteoglossomorphs and clupeocephalans, duplicates in *Pantodon* would have separate coalescents from other teleosts marking the independent cessation of recombination, and would fall as outgroups to the clupeocephalan teleost “a” and “b” clades, as we observed in many cases (e.g., *hoxb8y*, *hoxc13x*). Likewise, a weaker degree of genetic exchange between homeologous loci might allow some mutations between duplicates to become fixed, but subsequently cause them to swap their physical position between homeologous chromosomes, resulting in the interleaving pattern we observe (e.g., *hoxb8x*, *hoxb3x*). Nonreciprocal homeologous recombination (gene conversion) may also occur resulting in the segregation of two duplicates within a single orthology group (e.g., *hoxb6x*, *hoxb6y*). It may be impossible to mark a definitive point in time when genetic exchange between duplicated homeologous loci ceases completely. It is likely that homeologous loci diverge progressively, undergoing genetic exchange less and less frequently with time until finally it becomes so infrequent as to be effectively nonexistent. Using lineages which speciated close to the TGD event, we were able to use phylogenetic data to date the relative order that duplicated genomic segments achieved complete independence. With this method we can infer that the duplicated hoxa clusters diploidized first, prior to the speciation event separating osteoglossomorphs and clupeocephalans, whereas the hoxb, hoxc and hoxd clusters most likely concluded diploidization independently after divergence of these two lineages. Our model of teleost *Hox* cluster evolution which gives an account of the relative timing of diploidization of the different clusters in addition to detailing individual gene and whole cluster loss patterns is outlined in figure 5.

### Diploidization Dynamics in Other Polyploids

The types of complex genetic interactions between duplicated homeologous loci which are possible after polyploidization are best documented in plants, where allopolyploidy in particular is a pervasive phenomenon (Otto and Whitton 2000; Wendel 2000; Doyle and Egan 2010). The idea that sequence similarity between duplicated homeologous chromosomes might linger in allopolyploid plants causing tetrasomic inheritance was first proposed over 50 years ago (Stebbins 1947). Recent work in plants including artificially resynthesized allopolyploid lines in the genus *Brassica* (Gaeta et al. 2007), a natural assemblage of very recently (<80 years) formed allopolyploid species in the genus *Tragopogon* (Chester et al. 2012) and older (1–2 My) allopolyploid lineages such as the cotton *Gossypium hirsutum* (Salmon et al. 2010) has revealed molecular evidence for extensive genetic exchange between duplicated homeologous loci. In contrast to autopolyploidy where intraspecific genome doubling provides ample opportunity for tetrasomic inheritance, allopolyploidy comes in two categories which differ in the extent of genetic exchange expected between homeologous chromosomes: “Genomic” and “segmental” allopolyploidy (Stebbins 1971). Genomic allopolyploids result from the interspecific hybridization of two highly divergent parental species genomes causing homeologous chromosomes to exhibit almost immediate bivalent segregation and disomic inheritance. In these allopolyploids the genome is effectively prediploidized and the coalescent time of duplicated genes predates the date of hybrid formation, and instead corresponds to the speciation date of the parental species. Segmental allopolyploids, however, result from hybridization where only a fraction of the parental genomes is divergent enough for immediate bivalent formation, and other regions continue to exhibit tetrasomic inheritance and eventually must diploidize. Maize (*Zea mays*) which experienced a polyploidization event approximately 11.4 Ma seems to be a segmental allotetraploid in origin (Gaut and Doebley 1997). Our results are consistent with the ancient TGD being either an autopolyploidy event where the duplicated HoxA clusters diploidized before the HoxB, HoxC, and HoxD clusters, or a segmental allopolyploidy event in which the HoxA clusters of the parental species were effectively prediploidized immediately upon hybrid formation, but strongly exclude genomic allopolyploidy as an option.

Much less is known about the process of diploidization after ancient WGDs in animal lineages, especially in vertebrates. Tetravalent formation during meiosis, which favors homeologous recombination and gene conversion, has been directly observed in recently polyploidized lineages including autotetraploid frogs (*Odontophrynus americanus*) (Becak et al. 1966) and allotetraploid loaches (*Misgurnus anguillicaudatus*) (Li et al. 2011). Interestingly, salmonid teleosts, which underwent an additional independent WGD (sometimes called 4R) after the TGD between 25 and 100 Ma (Allendorf and Thorgaard 1984), still do not seem to have completed the diploidization of their genomes, as the recent analysis of the complete genome of the rainbow trout has demonstrated (Berthelot et al. 2014). Furthermore, there is evidence in the salmonid lineage for ongoing recombination between duplicated loci (Johnson et al. 1987; Allendorf and Danzmann 1997; Gharbi et al. 2006) and for occasional tetravalent formation during meiosis (Lee and Wright 1981; Allendorf and Thorgaard 1984). If the divergence of osteoglossomorphs and clupeocephalans occurred relatively rapidly following the TGD, it would therefore be conceivable that the diploidization process was only partially complete, and concluded independently following their divergence. The earliest recognizable fossils of the major teleost subdivisions (Osteoglossmorpha, Elopomorpha, Clupeocephala) only appear near the Jurassic–Cretaceous boundary approximately 150 Ma (Arratia 1997). Molecular estimates for the divergence time of crown teleosts vary widely according to whether mitochondrial (268–326 Ma) or nuclear (181–265 Ma) data are used (Hurley et al. 2007). Estimates for the timing of the TGD based on dating the divergence of paralogous genes in clupeocephalan teleosts range between 320 and 350 Ma (Taylor et al. 2001; Christoffels et al. 2004; Vandepoele et al. 2004). Using the median divergence time estimates for the teleost radiation and the TGD, respectively, we arrive at a very rough approximation of 38 My (mitochondrial) to 112 My (nuclear) between the TGD and the divergence of the major teleost subdivisions. Because of the strong dependence of the molecular clock used to make these estimates on the diploidization process itself, the relative dating of the TGD and teleost diversification may become somewhat confounded. However, even with this caveat in mind most authors concur it is likely that the period of time between the TGD and the teleost radiation was relatively brief. This brevity could conceivably have allowed for speciation to begin before diploidization had terminated.

### Tetralogy: A New Homology Subtype

We suggest that the classical concepts of orthology and paralogy are not adequate to describe the homology relationship between gene duplicates in two species which originated from a single duplication event, yet do not share a coalescent as a result of independent diploidization following speciation. We recommend the use of novel terminology for this subtype of molecular homology which will serve to quickly and accurately describe it: Tetralogy.

**Fig. 6.**
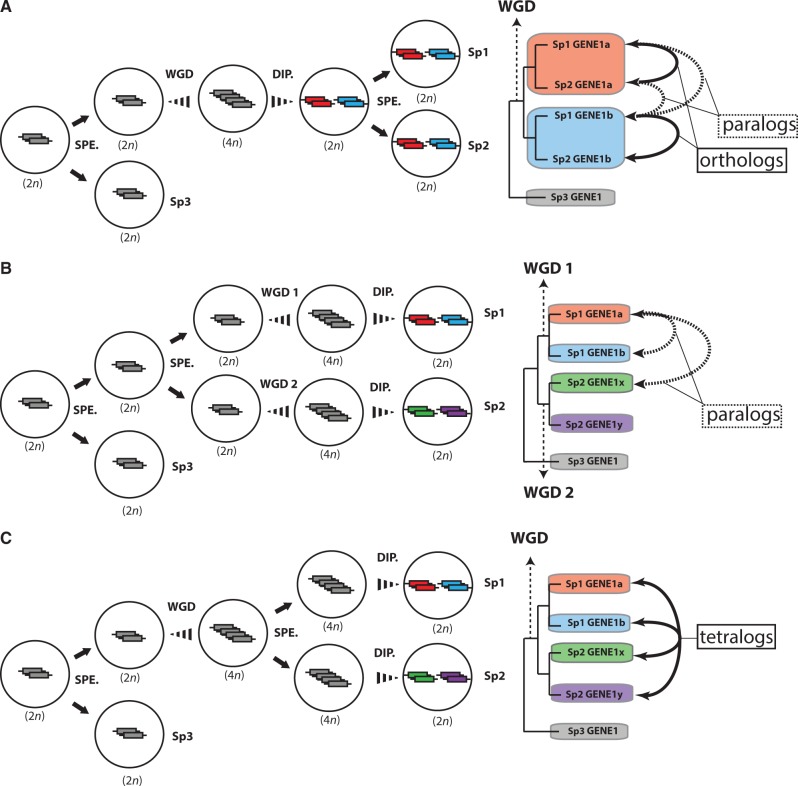
Types of homology relationships following WGD. Schematic outlining the types of homology relationships which can exist between gene duplicates as a result of WGD, taking into account the relative timing of diploidization. (*A*) The classical model where both WGD and full diploidization (DIP.) occur before speciation (SPE.) results in two types of homology relationships: Orthology (e.g., Sp1 GENE1b and Sp2 GENE1b) and paralogy (e.g., Sp1 GENE1a and Sp2 GENE1b). (*B*) The classical alternative scenario to the model presented in (A), where speciation occurs before two independent WGD and subsequent diploidization, only results in the formation of paralogs (e.g., Sp1 GENE1a and Sp2 GENE1x). (*C*) A new scenario where a single WGD occurs prior to speciation but where diploidization occurs independently in each lineage can result in a novel type of homology relationship: Tetralogy (e.g., Sp1 GENE 1a and Sp1 GENE 1b are tetralogous with Sp2 GENE 1x and Sp2 GENE 1y). Following duplication in a diploid (2*n*) ancestor, and the resultant tetraploid (4*n*) speciates prior to full diploidization, recombination will cease independently and loci will make the transition from 4*n* alleles to 2*n* paralogs separately in each lineage. Shared derived mutations between will then be able to accumulate independently between each pair of duplicates in each lineage, and no single duplicate in one lineage can be considered orthologous with a single duplicate in the other.

Under the classical model (single-WGD, shared diploidization), relationships between genes in two species which experienced a shared duplication (and shared diploidization) event prior to speciation resolve unambiguously into either 1:1 orthologs which descend from the same gene present in their last common ancestor, or paralogs which are descendants of two different duplicates in their last common ancestor (fig. 6*A*). If two separate duplication events of the same gene occurred independently following speciation (two-WGD model), 1:1 orthologs in the descendant species could not exist because the duplicates originated from two events and two distinct ancestral genes, and only paralogous relationships would exist (fig. 6*B*). In our model (single-WGD, independent diploidization) where two species share a duplication event but where duplicates diploidize into genetically independent loci from a shared allelic pool independently in the two lineages (fig. 6*C*), none of the resultant genes can be considered 1:1 orthologs because they do not share a single common ancestral sequence. However, they share an important property with the orthologous duplicates generated under the single-WGD shared diploidization model depicted in figure 6*A* in that they fundamentally originate from the exact same duplication event. This distinguishes them from the paralogous duplicates generated in the two-WGD model depicted in figure 6*B*. We use the term tetralogy to describe this unique type of 2:2 orthology relationship. Because the topology of individual gene trees resulting from independent diploidization is indistinguishable from those produced by independent duplication, only by examining multiple loci and syntenic genes can independent duplication be conclusively ruled out and tetralogy as a result of independent diploidization be inferred. When we compare phylogenetic trees of multiple genes across the *Hox* clusters of *Pantodon* and other teleosts, what we find is a combination of topologies. Some genes segregate in ways consistent with both the independent diploidization and independent duplication scenarios, whereas others segregate exactly as one would expect for a shared duplication and diploidization scenario. We can therefore infer that *Pantodon* did not experience a separate genome duplication event but rather that independent diploidization is likely to have occurred.

Because consistent *Hox* gene nomenclature which reflects both homology and physical position is needed, we advocate retaining the suffixes “*x*” and “*y*” for all *Hox* genes in *Pantodon*, including those individual genes which segregate within clupeocephalan “a” or “b” orthology groups but are part of a cluster which does not otherwise share unanimous 1:1 orthology with other teleost clusters. We also retain the naming system for members of the hoxax cluster mainly for consistency.

### Applications of the Tetralogy Concept to Outstanding Questions in Vertebrate Evolutionary Genomics

One area where the tetralogy concept may have profound implications concerns the outstanding question of whether osteoglossomorphs, elopomorphs, or a clade containing both is the sister clade to all other teleosts. Analysis using full mitochondrial genomes supports Osteoglossomorpha as the sister group to other teleosts (Inoue et al. 2001, 2003), whereas a more recent supertree analysis of nine nuclear gene exons suggests that Elopomorpha is the sister group (Near et al. 2012). Even some support for a combined (Osteoglossomorpha + Elopomorpha) sister clade to other teleosts has been found using ribosomal RNA genes (Le et al. 1993). In order for gene trees to have any hope of accurately reflecting real underlying evolutionary relationships between species, it is absolutely necessary to limit phylogenetic analysis to 1:1 orthologs. The problem of hidden paralogy, the mistaken use of paralogs rather than orthologs in phylogenetic trees as a result of a gene loss, is a known source of bias in phylogenomic analysis (Kuraku 2010). The prospect of tetralogous genes, that is genes which fundamentally do not have an unbroken lineage of 1:1 orthology between all species sharing a WGD event, adds a further complication to species tree reconstruction. In principle, any genes which cannot be shown to share a clearly orthologous relationship between species, including tetralogous genes, should not be used in phylogenomics. Although unduplicated mitochondrial genomes are not expected to be fraught with the same problems, all nuclear genes in teleosts used for phylogenomics should first be screened for potential hidden tetralogy, and only those loci which form well-supported orthologous relationships should be used. Hidden tetralogy will most likely only affect the phylogenetic relationships between species which diverged close to a WGD event and may not have experienced complete communal diploidization. Our results suggest that Osteoglossomorpha, Elopomorpha, and Clupeocephala may fit this criterion, and hidden tetralogy between phylogenetic markers of nuclear origin may be one reason why the relationship between these clades remains largely enigmatic.

A second outstanding controversy to which the concept of tetralogy may apply concerns the uncertainty surrounding the relative timing of the vertebrate 1R/2R WGD events to the divergence of agnathans and gnathostomes (Kuraku 2008; Shimeld and Donoghue 2012). Extant agnathans, comprising hagfish and lampreys, have many duplicated genes in similar proportions to gnathostomes, but when phylogenetic trees are constructed clear 1:1 orthologies with gnathostome genes are often absent (Escriva et al. 2002; Irvine et al. 2002; Stadler et al. 2004; Qiu et al. 2011; Fujimoto et al. 2013). Explanations to account for this have included agnathans and gnathostomes sharing only one genome duplication (plus additional duplications in each lineage), or multiple independent duplications in each lineage. Analysis of the a draft version of the sea lamprey (*Petromyzon marinus*) genome suggested that both 1R and 2R were likely shared between agnathans and gnathostomes, largely based on shared synteny (Smith et al. 2013). However the most thorough study of *Hox* genes in lamprey to date has found evidence of at least six Hox clusters implicating at least one independent round of genome duplication after 1R/2R in the lamprey lineage, and casting doubt on whether even 1R/2R was shared based partially on absence of 1:1 orthology of any lamprey genes with gnathostomes (Mehta et al. 2013). Our data suggest that, at least in the case of the TGD, gene trees do not necessarily resolve into clear orthology groups even following a single-shared genome duplication event and that the expectation of 1:1 orthology following a shared WGD may be unwarranted. One might speculate that if the ancestral vertebrate went through a prolonged tetraploid (or octoploid) stage with some regions of the genome diploidizing before divergence of agnathans and gnathostomes, and others diploidizing independently in each lineage, it could lead to the same type of incongruent phylogenetic patterns we observed in this study. Of course, these explanations are not mutually exclusive and it is possible that a combination of independent and shared genome duplications and diploidization events occurred.

## Conclusion

We have demonstrated that the *Hox* gene clusters of *P. buchholzi* represent a unique departure from the situation seen in other teleost fishes. *Pantodon* has fewer *Hox* gene clusters than any other teleost (five), but a similar total *Hox* gene number (45). We also observe that phylogenetic analysis of the *Hox* clusters of *P. buchholzi* fails to support a shared TGD hypothesis unless a model accounting for diploidization after duplication is considered. Under this model we propose that after a shared TGD, there was gradual and segmental diploidization of the genome which concluded independently in osteoglossomorphs and clupeocephalans, and that the last teleost common ancestor was therefore likely a pseudotetraploid. We argue that *Pantodon* shared TGD with other teleost fish, but that most of its *Hox* gene clusters (hoxbx/hoxby, hoxcx, hoxdx) loci violate assumptions of 1:1 orthology with clupeocephalan teleost *Hox* clusters as a result of independent diploidization. Therefore, instead of all duplicates which originated at the TGD showing clear 1:1 orthology across teleosts, some may share a novel type of 2:2 orthology relationship as a result of independent diploidization: Tetralogy. The prevalence of this phylogenetic pattern in other gene families across the genome of *Pantodon* and related osteoglossomorphs will be of significant interest and can be tested once additional genomic resources become available.

## Materials and Methods

### Genomic DNA Isolation

Adult specimens of *P. buchholzi* were obtained from retailers in the United Kingdom. Genomic DNA was prepared from muscle by standard proteinase K digestion and phenol-chloroform extraction.

### Isolation of Hox and Evx/Eve Probes by PCR

Degenerate primers (SO1 5′-garytngaraargartt-3′ and SO2 5′-cknckrttytgraacca-3′) were used to amplify *Hox* and related ANTP class homeobox-containing genes in *Pantodon*. Additional primers were designed to target-specific vertebrate *Hox* families PG1, PG5, PG9, and PG13: (Hox1SO1 5′-acvgarytngaraargartt-3′ and Hox1SO2 5′-catncknckrttytgraacca-3′), (Hox5F1 5′-gcntayacbcgytaycagac-3′, used with SO2), (Hox9F1 5′-tgyccytayacnaaryayca-3′, used with SO2), (Hox13F1 5′–aarmgnrtnccntayasnaa-3′ and Hox13SO2 5′-acnckbckrttytgraacca-3′). Primers (EvxF 5′–yaycgngcnttcacvmgnga-3′ and EvxR 5′-ckctgnckyttgtccttcat-3′) were used to amplify *Evx* family genes. Amplified bands resulting from gradients of annealing temperature from 45 °C to 55 °C and 0.5–5.0 mM MgCl_2_ were gel extracted and cloned in the pGEM-T vector. Sanger sequencing was performed using a standard BigDye protocol. In excess of 200 clones were sequenced identified by similarity to other vertebrate genes by BLAST against the NCBI nr database online.

### BAC Library Screening

An approximately 6 × BAC library for *P. buchholzi* containing 27,648 clones with an average insert size of 138 kb was prepared by Amplicon Express from genomic DNA extracted from muscle tissue of five mixed sex individuals. Digoxigenin (DIG)-labeled probes were synthesized by PCR from cloned *Hox* and *Evx* gene fragments. Five pools of *Hox* probes and three separate *Evx* probes were synthesized. Arrayed BAC library filters were hybridized under relaxed stringency conditions (35–42 °C in Easy Hyb buffer), and washed with 2× SSC, 0.1% SDS, then 0.2 × SSC, 0.1% SDS (25–42 °C), and finally in wash buffer (0.1 M maleic acid, 0.3% Tween, 25–42 °C). Detection used anti-DIG fab fragment and CDP Star. Two library screens, one with the 35 combined *Hox* probes and one with the three *Evx* probes, were performed and the results yielded 82 candidate hox cluster BACs which were then midi-prepped and screened by PCR for the presence of *Hox* or *Evx* genes.

### Isolation and Sequencing of BAC Clones

DNA from ten BACs comprising the full diversity of amplified probes (57D22, 23G6, 39B7, 1I16, 27G8, 7O2, 3P22, 31M4, 22E23, and 42K2) was pooled and used to prepare a single-end library for Roche 454 pyrosequencing; one-quarter plate gave targeted sequence coverage of approximately 30 × , and is referred to Library “E.” The additional 48 of the original 72 candidate BACs isolated by hybridization, but which did not yield known *Hox* or other homeobox gene fragments by PCR, were prepared for sequencing as eight pools of separate cultures of six BACs each. These BAC pools consisted of pool A (1E1, 2H15, 3I22, 5P8, 10E24, 10M4), pool B (14D16, 16K5, 17M5, 19D4, 19N18, 21J10), pool C (22I13, 22A23, 26A20, 28C18, 29I4, 30I11), pool D (33B5, 33D19, 37H21, 39J24, 41C14, 41B1), pool E (42H17, 44N19, 44O19, 49E9, 49M13, 56F18), pool F (51H11, 51L3, 51N5, 52E9, 52H23, 53N20), pool G (57L10, 58N11, 59D23, 61N19, 64F9, 47B22), and pool H (65E11, 65I16, 66K2, 67O21, 67F12, 72O20). DNA from these pools, plus an aliquot of library “E,” was mixed to give library “G,” and used to prepare a 3-kb paired-end library for Roche 454 sequenced at sequencing approximately 8× coverage on one-quarter plate.

Sequenced libraries “E” and “G” were assembled together with the Roche Newbler assembler version 2.6. Assembly resulted in 251 scaffolds. Scaffolds comprising the full *Hox* clusters of *Pantodon* for hoxax (200,509 bp), hoxbx (379,906 bp), hoxby (370,487 bp), hoxcx (247,835 bp), and hoxdx (254,542 bp) were identified by BLAST, and confirmed to contain the entire length of the original ten best BACs by end sequencing the clones using Sanger technology. Each cluster was spanned by two BACs from the original ten BACs sequenced in library “E.”

### Whole-Genome Sequencing and the TRAM

Five micrograms of DNA from a single adult male specimen of *P. buchholzi* was used for Illumina HiSeq sequencing at the Wellcome Trust Center for Human Genetics, Oxford, using a 100-bp paired-end protocol and 180-bp insert size library. In total, 171 million reads, corresponding to approximately 22.9 × coverage of the approximately 753-Mb genome, were generated and used to build both local BLAST and HMMER search databases. To seed the searches against these databases, all vertebrate homeodomain protein sequences were downloaded from the homeodb web server (http://homeodb.zoo.ox.ac.uk/, last accessed July 4, 2014). Relaxed stringency tBLASTn search (*E* = 10) returned 14,297 sequences, which were extracted from the 171-million read database using a custom bash script, and were assembled using cap3 to produce 378 consensus sequences with a mean size of 227 bp and an N50 of 233 bp, corresponding to the approximately 180-bp homeobox-encoding exon. HMMER searches were performed using the PFAM homeobox.hmm profile and the hmmsearch algorithm to search the 6-frame translation of the reads. In total, 9,881 reads were extracted and assembled with cap3 (Huang 1999) into 338 consensus sequences. Cross-referencing the hmmsearch and tBLASTn results revealed 456 unique consensus sequences which represent putative homeobox sequences from the *Pantodon* genome. These were annotated using tBLASTn to the homeodb database and to a manually curated set of vertebrate *Hox* genes.

### Annotation of *Hox* Cluster Scaffolds

Initial ab initio predictions of all coding genes in the BAC assembly scaffolds were performed with genscan and verified by BLASTx searches against NCBI nr database. Only predicted proteins with high similarity (*E* < 10^−^^3^) to known proteins were retained for further annotation. Predicted *Hox* gene sequences were mapped to the scaffolds and predicted coding sequences of these scaffolds were refined by BLASTn searches to the NCBI nr database and ClustalW multiple sequence alignment of predicted genes with the *Hox* gene-coding sequences of *D. rerio, Ta. rubripes, G. aculeatus, Te. nigroviridis, Homo sapiens, Xenopus tropicalis,* and *Gallus gallus* to manually refine predicted start codons, stop codons, and splice sites. The five *Hox* cluster scaffold sequences of *P. buchholzi* are deposited in GenBank under accession numbers KM102157–KM102161.

### Phylogenetic Analysis

Vertebrate species chosen for phylogenetic analysis were the clupeocephalan teleosts *D. rerio*, *Salmo salar, O. latipes, G. aculeatus, Ta. rubripes, Te. nigroviridis,* and *Astatotilapia burtoni*, the elopomorph teleost *A. anguilla* and sarcopterygian outgroups *Latimeria menadoensis, X. tropicalis, Anolis caroliensis, Mus musculus* and *H. sapiens* as well as the chondrichthyan *C. milii*. Full-length protein-coding Hox gene sequences from five complete teleost genomes *D. rerio* (zv9), *O. latipes* (MEDAKA1), *G. aculeatus* (BROADS1), *Ta. rubripes* (FUGU4), and *Te. nigroviridis* (TETRAODON8) as well as the sarcopterygian outgroups *X. tropicalis* (JGI_4.2), *A. caroliensis* (AnoCar2.0), *M. musculus* (GRCm38), and *H. sapiens* (GRCh37) were downloaded using the export function of the Ensembl genome browser (http://www.ensembl.org, last accessed July 4, 2014). *Salmo salar* sequences were obtained from NCBI accession numbers (EF695248–EF695353; EU02568–EU025719; EU221640–EU221655), *C. milli* from NCBI accession numbers (FJ824598–FJ824601), *L. menadoensis* from NCBI accession numbers (FJ497005–FJ497008), *A. burtoni* from NCBI accession numbers (EF594310–EF594316), and *A. anguilla* from NCBI accession numbers (JF891390–JF891400). Alignments for all individual Hox gene families were performed on amino acid sequences using ClustalW (Larkin et al. 2007) and trimmed with trimAl (Capella-Gutiérrez et al. 2009) using a gap threshold of 25%. Alignments used in the AU likelihood ratio tests were built using all available Hox gene family sequences, concatenated after separate alignment. ML trees were built using RAxML (Stamatakis 2006) with the LG amino acid substitution model with gamma and invariable parameters, and 1,000 bootstrap replicates. Bayesian trees were built using MrBayes (Huelsenbeck and Ronquist 2001) using the WAG amino acid substitution model and the invariable and gamma parameters across four chains for 500,000 generations and checked for convergence. Alternative topologies for likelihood ratio tests were constructed by building sets of three constrained trees containing three branches each—the clupeocephalan post-TGD “a” constrained branch, the clupeocephalan post-TGD “b” constrained branch, and an unduplicated outgroup branch—and forcing the individual Pantodon genes, or the whole concatenated cluster, to segregate alternatively within the “a” (topology A), “b” (topology B), or outgroup clades (topology O). Anguilla sequences were left unconstrained. The total tree and site-wise likelihood of each constrained topology was calculated using RAxML and the AU test (Shimodaira 2002) was performed with the program consel (Shimodaira and Hasegawa 2001). In each case, the unconstrained best topology was also built with RAxML and compared with the three constrained topologies. 3D-SLRPs were constructed using R.

### CNE Analysis

The mVISTA application of VISTA-tools (http://genome.lbl.gov/vista/index.shtml, last accessed July 4, 2014) using the SLAGAN algorithm was used to form VISTA plots of *Pantodon* and other vertebrate *hox* gene clusters. CNEs using the Elephant shark *C. millii* sequence as the baseline were predicted using the cutoff threshold 70% identity over 100 bp. CNEs were downloaded, and the total number, length, and average size were counted using homemade PERL scripts.

### Selection Analysis

For selection analyses, individual *Hox* gene trees were constrained by assuming *Pantodon* shared the TGD and cluster diploidization with other teleosts and forced all *Pantodon* sequences in a single cluster within either the post-TGD “a” or “b” orthology groups as follows (hoxax + hoxaa, hoxbx + hoxba, hoxby + hoxbb, hoxcx + hoxca, hoxdx + hoxda) and built these constrained trees using RAxML with the same parameters as the unconstrained trees. We estimated selection on the most proximate post-TGD branches (“a” + “x,” “b” + “y”) using local branch models and branch-site models fit to the resultant ML trees using the software package HYPHY (Kosakovsky Pond et al. 2005). First, the MG94 codon substitution model (Muse and Gaut 1994) was fit locally to each individual branch to estimate d*N*, d*S*, and ω using the AnalyzeCodonData.bf function in HYPHY. Next, we used the BranchSiteREL.bf function of HYPHY to fit the MG94 model allowing three independent estimates of ω (ω^−^, ω^0^, ω^+^) along each branch to representing negative, neutral, and positive selection, respectively, quantified the proportion of sites (p^+^) which appeared to evolve under positive selection.

## Supplementary Material

Supplementary figures S1–S4 and tables S1–S5 are available at *Molecular Biology and Evolution* online (http://www.mbe.oxfordjournals.org/).

Supplementary Data

## References

[msu202-B1] Alfaro ME, Santini F, Brock C, Alamillo H, Dornburg A, Rabosky DL, Carnevale G, Harmon LJ (2009). Nine exceptional radiations plus high turnover explain species diversity in jawed vertebrates. Proc Natl Acad Sci U S A..

[msu202-B2] Allendorf FW, Danzmann RG (1997). Secondary tetrasomic segregation of MDH-B and preferential pairing of homeologues in rainbow trout. Genetics.

[msu202-B3] Allendorf FW, Thorgaard GH, Turner BJ (1984). Tetraploidy and the evolution of salmonid fishes.

[msu202-B4] Altschul SF, Gish W, Miller W, Myers EW, Lipman DJ (1990). Basic Local Alignment Search Tool. J Mol Biol..

[msu202-B5] Amores A, Force A, Yan Y-L, Joly L, Amimiya C, Fritz A, Ho RK, Langeland J, Prince V, Wang Y-L (1998). Zebrafish hox clusters and vertebrate genome evolution. Science.

[msu202-B6] Amores A, Suzuki T, Yan Y-L, Pomeroy J, Singer A, Amemiya C, Postlethwait JH (2004). Developmental roles of pufferfish Hox clusters and genome evolution in ray-fin fish. Genome Res..

[msu202-B7] Arratia G (1997). Basal teleosts and teleostean phylogeny. Palaeo Ichthyol..

[msu202-B8] Becak M, Becak W, Rabello M (1966). Cytological evidence of constant tetraploidy in the bisexual South American frog *Odontophrynus americanus*. Chromosoma.

[msu202-B9] Berthelot C, Brunet F, Chalopin D, Juanchich A, Bernard M, Noël B, Bento P, Da Silva C, Labadie K, Alberti A (2014). The rainbow trout genome provides novel insights into evolution after whole-genome duplication in vertebrates. Nat Commun..

[msu202-B10] Capella-Gutiérrez S, Silla-Martínez JM, Gabaldón T (2009). trimAl: a tool for automated alignment trimming in large-scale phylogenetic analyses. Bioinformatics.

[msu202-B11] Chambers KE, McDaniell R, Raincrow JD, Deshmukh M, Stadler PF, Chiu C (2009). Hox cluster duplication in the basal teleost *Hiodon alosoides* (Osteoglossomorpha). Theory Biosci..

[msu202-B12] Chester M, Gallagher JP, Symonds VV, Cruz da Silva AV, Mavrodiev EV, Leitch AR, Soltis PS, Soltis DE (2012). Extensive chromosomal variation in a recently formed natural allopolyploid species, *Tragopogon miscellus* (Asteraceae). Proc Natl Acad Sci U S A..

[msu202-B13] Christoffels A, Koh EGL, Chia J-M, Brenner S, Aparicio S, Venkatesh B (2004). Fugu genome analysis provides evidence for a whole-genome duplication early during the evolution of ray-finned fishes. Mol Biol Evol..

[msu202-B14] Coppe A, Pujolar JM, Maes GE, Larsen PF, Hansen MM, Bernatchez L, Zane L, Bortoluzzi S (2010). Sequencing, de novo annotation and analysis of the first *Anguilla anguilla* transcriptome: EeelBase opens new perspectives for the study of the critically endangered European eel. BMC Genomics.

[msu202-B15] Crow KD, Smith CD, Cheng J-F, Wagner GP, Amemiya CT (2012). An independent genome duplication inferred from Hox paralogs in the American paddlefish—a representative basal ray-finned fish and important comparative reference. Genome Biol Evol..

[msu202-B16] Crow KD, Stadler PF, Lynch VJ, Amemiya C, Wagner GP (2006). The “fish-specific” Hox cluster duplication is coincident with the origin of teleosts. Mol Biol Evol..

[msu202-B17] Dayhoff M (1976). Origin and evolution of protein superfamilies. Fed Proc..

[msu202-B18] Demuth JP, Hahn MW (2009). The life and death of gene families. BioEssays.

[msu202-B19] Dolinski K, Botstein D (2007). Orthology and functional conservation in eukaryotes. Annu Rev Genet..

[msu202-B20] Doyle JJ, Egan AN (2010). Dating the origins of polyploidy events. New Phytol..

[msu202-B21] Edger PP, Pires JC (2009). Gene and genome duplications: the impact of dosage-sensitivity on the fate of nuclear genes. Chromosome Res..

[msu202-B22] Escriva H, Manzon L, Youson J, Laudet V (2002). Analysis of lamprey and hagfish genes reveals a complex history of gene duplications during early vertebrate evolution. Mol Biol Evol..

[msu202-B23] Fitch WM (1970). Distinguishing homologous from analogous proteins. Syst Zool..

[msu202-B24] Freeling M, Thomas BC (2006). Gene-balanced duplications, like tetraploidy, provide predictable drive to increase morphological complexity. Genome Res..

[msu202-B25] Fujimoto S, Oisi Y, Kuraku S, Ota KG, Kuratani S (2013). Non-parsimonious evolution of hagfish Dlx genes. BMC Evol Biol..

[msu202-B26] Gabaldón T, Koonin EV (2013). Functional and evolutionary implications of gene orthology. Nat Rev Genet..

[msu202-B27] Gaeta RT, Pires JC, Iniguez-Luy F, Leon E, Osborn TC (2007). Genomic changes in resynthesized *Brassica napus* and their effect on gene expression and phenotype. Plant Cell.

[msu202-B28] Garcia-Fernàndez J, Holland PWH (1994). Archetypal organization of the amphioxus Hox gene cluster. Nature.

[msu202-B29] Gaut BS, Doebley JF (1997). DNA sequence evidence for the segmental allotetraploid origin of maize. Proc Natl Acad Sci U S A..

[msu202-B30] Gharbi K, Gautier A, Danzmann RG, Gharbi S, Sakamoto T, Hoyheim B, Taggart JB, Cairney M, Powell R, Krieg F (2006). A linkage map for brown trout (*Salmo trutta*): chromosome homeologies and comparative genome organization with other salmonid fish. Genetics.

[msu202-B31] Henkel CV, Burgerhout E, De Wijze DL, Dirks RP, Minegishi Y, Jansen HJ, Spaink HP, Dufour S, Weltzien F-A, Tsukamoto K (2012). Primitive duplicate Hox clusters in the European eel’s genome. PLoS One.

[msu202-B32] Henkel CV, Dirks RP, De Wijze DL, Minegishi Y, Aoyama J, Jansen HJ, Turner B, Knudsen B, Bundgaard M, Hvam KL (2012). First draft genome sequence of the Japanese eel, *Anguilla japonica*. Gene.

[msu202-B33] Hilton EJ (2003). Comparative osteology and phylogenetic systematics of fossil and living bony-tongue fishes (Actinopterygii, Teleostei, Osteoglossomorpha). Zool J Linn Soc..

[msu202-B34] Hinegardner R, Rosen DE (1972). Cellular DNA content and the evolution of teleostean fishes. Museum.

[msu202-B35] Hoegg S, Boore JL, Kuehl JV, Meyer A (2007). Comparative phylogenomic analyses of teleost fish Hox gene clusters: lessons from the cichlid fish *Astatotilapia burtoni*. BMC Genomics.

[msu202-B36] Hoegg S, Brinkmann H, Taylor JS, Meyer A (2004). Phylogenetic timing of the fish-specific genome duplication correlates with the diversification of teleost fish. J Mol Evol..

[msu202-B37] Holland PW, Garcia-Fernàndez J, Williams NA, Sidow A (1994). Gene duplications and the origins of vertebrate development. Development.

[msu202-B38] Huang X (1999). CAP3: A DNA sequence assembly program. Genome Res..

[msu202-B39] Huelsenbeck JP, Ronquist F (2001). MRBAYES: Bayesian inference of phylogenetic trees. Bioinformatics.

[msu202-B40] Hurley IA, Mueller RL, Dunn KA, Schmidt EJ, Friedman M, Ho RK, Prince VE, Yang Z, Thomas MG, Coates MI (2007). A new time-scale for ray-finned fish evolution. Proc R Soc Lond B Biol Sci..

[msu202-B41] Inoue JG, Miya M, Tsukamoto K, Nishida M (2001). A mitogenomic perspective on the basal teleostean phylogeny: resolving higher-level relationships with longer DNA sequences. Mol Phylogenet Evol..

[msu202-B42] Inoue JG, Miya M, Tsukamoto K, Nishida M (2003). Basal actinopterygian relationships: a mitogenomic perspective on the phylogeny of the “ancient fish.”. Mol Phylogenet Evol..

[msu202-B43] Irvine SQ, Carr JL, Bailey WJ, Kawasaki K, Shimizu N, Amemiya CT (2002). Genomic analysis of Hox clusters in the sea lamprey *Petromyzon marinus*. J Exp Zool..

[msu202-B44] Jaillon O, Aury J-M, Brunet F, Petit J-L, Strange-Thomann N, Mauceli E, Bouneau L, Fischer C, Ozouf-Costaz C, Bernot A (2004). Genome duplication in the teleost fish *Tetraodon nigroviridis* reveals the early vertebrate proto-karyotype. Nature.

[msu202-B45] Johnson KP, Walden KKO, Robertson HM (2013). Next-generation phylogenomics using a Target Restricted Assembly Method. Mol Phylogenet Evol..

[msu202-B46] Johnson KR, Wright JE, May B (1987). Linkage relationships reflecting ancestral tetraploidy in salmonid fish. Genetics.

[msu202-B47] King BL, Gillis JA, Carlisle HR, Dahn RD (2011). A natural deletion of the HoxC cluster in elasmobranch fishes. Science.

[msu202-B48] Koonin EV (2005). Orthologs, paralogs, and evolutionary genomics. Annu Rev Genet..

[msu202-B49] Kosakovsky Pond SL, Murrell B, Fourment M, Frost SD, Delport W, Scheffler K (2011). A random effects branch-site model for detecting episodic diversifying selection. Mol Biol Evol..

[msu202-B50] Kuraku S (2008). Insights into cyclostome phylogenomics: pre-2R or post-2R. Zool Sci..

[msu202-B51] Kuraku S (2010). Palaeophylogenomics of the vertebrate ancestor—impact of hidden paralogy on hagfish and lamprey gene phylogeny. Integr Comp Biol..

[msu202-B52] Larkin MA, Blackshields G, Brown NP, Chenna R, McGettigan PA, McWilliam H, Valentin F, Wallace IM, Wilm A, Lopez R (2007). Clustal W and Clustal X version 2.0. Bioinformatics.

[msu202-B53] Le HLV, Lecointre G, Perasso R (1993). A 28S rRNA-based phylogeny of the gnathostomes—first steps in athe analysis of conflict and congruence with morphologically based cladeograms. Mol Phylogenet Evol..

[msu202-B54] Lee AP, Kerk SY, Tan YY, Brenner S, Venkatesh B (2010). Ancient vertebrate conserved noncoding elements have been evolving rapidly in teleost fishes. Mol Biol Evol..

[msu202-B55] Lee GM, Wright JE (1981). Mitotic and meiotic analyses of brook trout, *Salvelinus fontinalis*. J Hered..

[msu202-B56] Li WH, Gu Z, Wang H, Nekrutenko A (2001). Evolutionary analyses of the human genome. Nature.

[msu202-B57] Li Y-J, Yu Z, Zhang M-Z, Qian C, Abe S, Arai K (2011). The origin of natural tetraploid loach *Misgurnus anguillicaudatus* (Teleostei: Cobitidae) inferred from meiotic chromosome configurations. Genetica.

[msu202-B58] Liang D, Wu R, Geng J, Wang C, Zhang P (2011). A general scenario of Hox gene inventory variation among major sarcopterygian lineages. BMC Evol Biol..

[msu202-B59] Lin Z, Ma H, Nei M (2008). Ultraconserved coding regions outside the homeobox of mammalian Hox genes. BMC Evol Biol..

[msu202-B60] Mable BK (2004). “Why polyploidy is rarer in animals than in plants”: myths and mechanisms. Biol J Linn Soc Lond..

[msu202-B61] Makino T, McLysaght A (2010). Ohnologs in the human genome are dosage balanced and frequently associated with disease. Proc Natl Acad Sci U S A..

[msu202-B62] Málaga-Trillo E, Meyer A (2001). Genome duplications and accelerated evolution of Hox genes and cluster architecture in teleost fishes. Am Zool..

[msu202-B63] Mehta TK, Ravi V, Yamasaki S, Lee AP, Lian MM, Tay B-H, Tohari S, Yanai S, Tay A, Brenner S (2013). Evidence for at least six Hox clusters in the Japanese lamprey (*Lethenteron japonicum*). Proc Natl Acad Sci U S A..

[msu202-B64] Metzker ML (2009). Sequencing technologies—the next generation. Nat Rev Genet..

[msu202-B65] Meyer A, Van de Peer Y (2005). From 2R to 3R: evidence for a fish-specific genome duplication (FSGD). BioEssays.

[msu202-B66] Mungpakdee S, Seo H-C, Angotzi AR, Dong X, Akalin A, Chourrout D (2008). Differential evolution of the 13 Atlantic salmon Hox clusters. Mol Biol Evol..

[msu202-B67] Muse SV, Gaut BS (1994). A likelihood approach for comparing synonymous and nonsynonymous nucleotide substitution rates, with application to the chloroplast genome. Mol Biol Evol..

[msu202-B68] Near TJ, Eytan RI, Dornburg A, Kuhn KL, Moore JA, Davis MP, Wainwright PC, Friedman M, Smith WL (2012). Resolution of ray-finned fish phylogeny and timing of diversification. Proc Natl Acad Sci U S A..

[msu202-B69] Nehrt NL, Clark WT, Radivojac P, Hahn MW (2011). Testing the ortholog conjecture with comparative functional genomic data from mammals. PLoS Comput Biol..

[msu202-B70] Nei M, Rooney AP (2005). Concerted and birth-and-death evolution of multigene families. Annu Rev Genet..

[msu202-B71] Ohno S (1970). Evolution by gene duplication.

[msu202-B72] Otto SP (2007). The evolutionary consequences of polyploidy. Cell.

[msu202-B73] Otto SP, Whitton J (2000). Polyploid incidence and evolution. Annu Rev Genet..

[msu202-B74] Oulion S, Debiais-Thibaud M, d’Aubenton-Carafa Y, Thermes C, Da Silva C, Bernard-Samain S, Gavory F, Wincker P, Mazan S, Casane D (2010). Evolution of Hox gene clusters in gnathostomes: insights from a survey of a shark (*Scyliorhinus canicula*) transcriptome. Mol Biol Evol..

[msu202-B75] Pascual-Anaya J, D’Aniello S, Kuratani S, Garcia-Fernàndez J (2013). Evolution of Hox gene clusters in deuterostomes. BMC Dev Biol..

[msu202-B76] Pennacchio LA, Ahituv N, Moses AM, Prabhakar S, Nobrega MA, Shoukry M, Minovitsky S, Dubchak I, Holt A, Lewis KD (2006). In vivo enhancer analysis of human conserved non-coding sequences. Nature.

[msu202-B77] Pick L, Heffer A (2012). Hox gene evolution: multiple mechanisms contributing to evolutionary novelties. Ann N Y Acad Sci..

[msu202-B78] Pond SLK, Frost SDW, Muse SV (2005). HyPhy: hypothesis testing using phylogenies. Bioinformatics.

[msu202-B79] Putnam NH, Butts T, Ferrier DEK, Furlong RF, Hellsten U, Kawashima T, Robinson-Rechavi M, Shoguchi E, Terry A, Yu J-K (2008). The amphioxus genome and the evolution of the chordate karyotype. Nature.

[msu202-B80] Qiu H, Hildebrand F, Kuraku S, Meyer A (2011). Unresolved orthology and peculiar coding sequence properties of lamprey genes: the KCNA gene family as test case. BMC Genomics.

[msu202-B81] Ravi V, Lam K, Tay B-H, Tay A, Brenner S, Venkatesh B (2009). Elephant shark (*Callorhinchus milii*) provides insights into the evolution of Hox gene clusters in gnathostomes. Proc Natl Acad Sci U S A..

[msu202-B82] Ravi V, Venkatesh B (2008). Rapidly evolving fish genomes and teleost diversity. Curr Opin Genet Dev..

[msu202-B83] Rokas A, Williams BL, King N, Carroll SB (2003). Genome-scale approaches to resolving incongruence in molecular phylogenies. Nature.

[msu202-B84] Salmon A, Flagel L, Ying B, Udall JA, Wendel JF (2010). Homoeologous nonreciprocal recombination in polyploid cotton. New Phytol..

[msu202-B85] Shimeld SM, Donoghue PCJ (2012). Evolutionary crossroads in developmental biology: cyclostomes (lamprey and hagfish). Development.

[msu202-B86] Shimodaira H (2002). An approximately unbiased test of phylogenetic tree selection. Syst Biol..

[msu202-B87] Shimodaira H, Hasegawa M (2001). CONSEL: for assessing the confidence of phylogenetic tree selection. Bioinformatics.

[msu202-B88] Sidow A (1996). Gen(om)e duplications in the evolution of early vertebrates. Curr Opin Genet Dev..

[msu202-B89] Smith JJ, Kuraku S, Holt C, Sauka-Spengler T, Jiang N, Campbell M, Yandell MD, Manousaki T, Meyer A, Bloom OE (2013). Sequencing of the sea lamprey (*Petromyzon marinus*) genome provides insights into vertebrate evolution. Nat Genet..

[msu202-B90] Söding J (2005). Protein homology detection by HMM-HMM comparison. Bioinformatics.

[msu202-B91] Stadler PF, Fried C, Prohaska SJ, Bailey WJ, Misof BY, Ruddle FH, Wagner GP (2004). Evidence for independent Hox gene duplications in the hagfish lineage: a PCR-based gene inventory of *Eptatretus stoutii*. Mol Phylogenet Evol..

[msu202-B92] Stamatakis A (2006). RAxML-VI-HPC: maximum likelihood-based phylogenetic analyses with thousands of taxa and mixed models. Bioinformatics.

[msu202-B93] Stebbins GL (1947). Types of polyploids; their classification and significance. Adv Genet..

[msu202-B95] Studer RA, Robinson-Rechavi M (2009). How confident can we be that orthologs are similar, but paralogs differ?. Trends Genet..

[msu202-B96] Sullivan JP (2004). Simultaneous analysis of five molecular markers provides a well-supported phylogenetic hypothesis for the living bony-tongue fishes (Osteoglossomorpha: Teleostei). Mol Phylogenet Evol..

[msu202-B97] Taylor JS, Van de Peer Y, Meyer A (2001). Revisiting recent challenges to the ancient fish-specific genome duplication hypothesis. Curr Biol..

[msu202-B98] Thornton JW, DeSalle R (2000). Gene family evolution and homology: genomics meets phylogenetics. Annu Rev Genomics Hum Genet..

[msu202-B99] Tümpel S, Cambronero F, Wiedemann LM, Krumlauf R (2006). Evolution of cis elements in the differential expression of two Hoxa2 coparalogous genes in pufferfish (*Takifugu rubripes*). Proc Natl Acad Sci U S A..

[msu202-B100] Van de Peer Y, Maere S, Meyer A (2009). The evolutionary significance of ancient genome duplications. Nat Rev Genet..

[msu202-B101] Van de Peer Y, Maere S, Meyer A (2010). 2R or not 2R is not the question anymore. Nat Rev Genet..

[msu202-B102] Vandepoele K, De Vos W, Taylor JS, Meyer A, Van de Peer Y (2004). Major events in the genome evolution of vertebrates: paranome age and size differ considerably between ray-finned fishes and land vertebrates. Proc Natl Acad Sci U S A..

[msu202-B103] Venkatesh B, Kirkness EF, Loh Y-H, Halpern AL, Lee AP, Johnson J, Dandona N, Viswanathan LD, Tay A, Venter JC (2006). Ancient noncoding elements conserved in the human genome. Science.

[msu202-B104] Wada H, Garcia-Fernàndez J, Holland PW (1999). Colinear and segmental expression of amphioxus Hox genes. Dev Biol..

[msu202-B105] Wendel JF (2000). Genome evolution in polyploids. Plant Mol Biol..

[msu202-B106] White S (1994). Out of deepest Africa. Tropical Fish Hobbyist.

[msu202-B107] Wolfe KH (2001). Yesterday’s polyploids and the mystery of diploidization. Nat Rev Genet..

[msu202-B108] Woltering JM, Durston AJ (2006). The zebrafish hoxDb cluster has been reduced to a single microRNA. Nat Genet..

[msu202-B109] Woolfe A, Goodson M, Goode DK, Snell P, McEwan GK, Vavouri T, Smith SF, North P, Callaway H, Krys K (2005). Highly conserved non-coding sequences are associated with vertebrate development. PLoS Biol..

[msu202-B110] Zhong Y-F, Butts T, Holland PWH (2008). HomeoDB: a database of homeobox gene diversity. Evol Dev..

