# Supplementary material for: Enigmatic Orthology Relationships between Hox Clusters of the African Butterfly Fish and Other Teleosts Following Ancient Whole-Genome Duplication
Source: Mol Biol Evol. 2014 Jun 27;31(10):2592–611. doi: 10.1093/molbev/msu202 (PMC4166920; doi:10.1093/molbev/msu202)
Supplement: Supplementary Data [file supp_31_10_2592__index.html]

Enigmatic Orthology Relationships between Hox Clusters of the African Butterfly Fish and Other Teleosts Following Ancient Whole-Genome Duplication — Enigmatic Orthology Relationships between Hox Clusters of the African Butterfly Fish and Other Teleosts Following Ancient Whole-Genome Duplication — Supplementary Data 

# Enigmatic Orthology Relationships between *Hox* Clusters of the African Butterfly Fish and Other Teleosts Following Ancient Whole-Genome Duplication

## Supplementary Data

file

**Files in this Data Supplement:**

- Supplementary Data - pdf file
- Supplementary Data - pdf file
- Supplementary Data - xlsx file
