# Supplementary material for: Enigmatic Orthology Relationships between Hox Clusters of the African Butterfly Fish and Other Teleosts Following Ancient Whole-Genome Duplication
Source: Mol Biol Evol. 2014 Jun 27;31(10):2592–611. doi: 10.1093/molbev/msu202 (PMC4166920; doi:10.1093/molbev/msu202)
Supplement: Supplementary Data [file supp_msu202_SI_FigS1_ML_Trees_FigS2_mb_Trees.pdf]

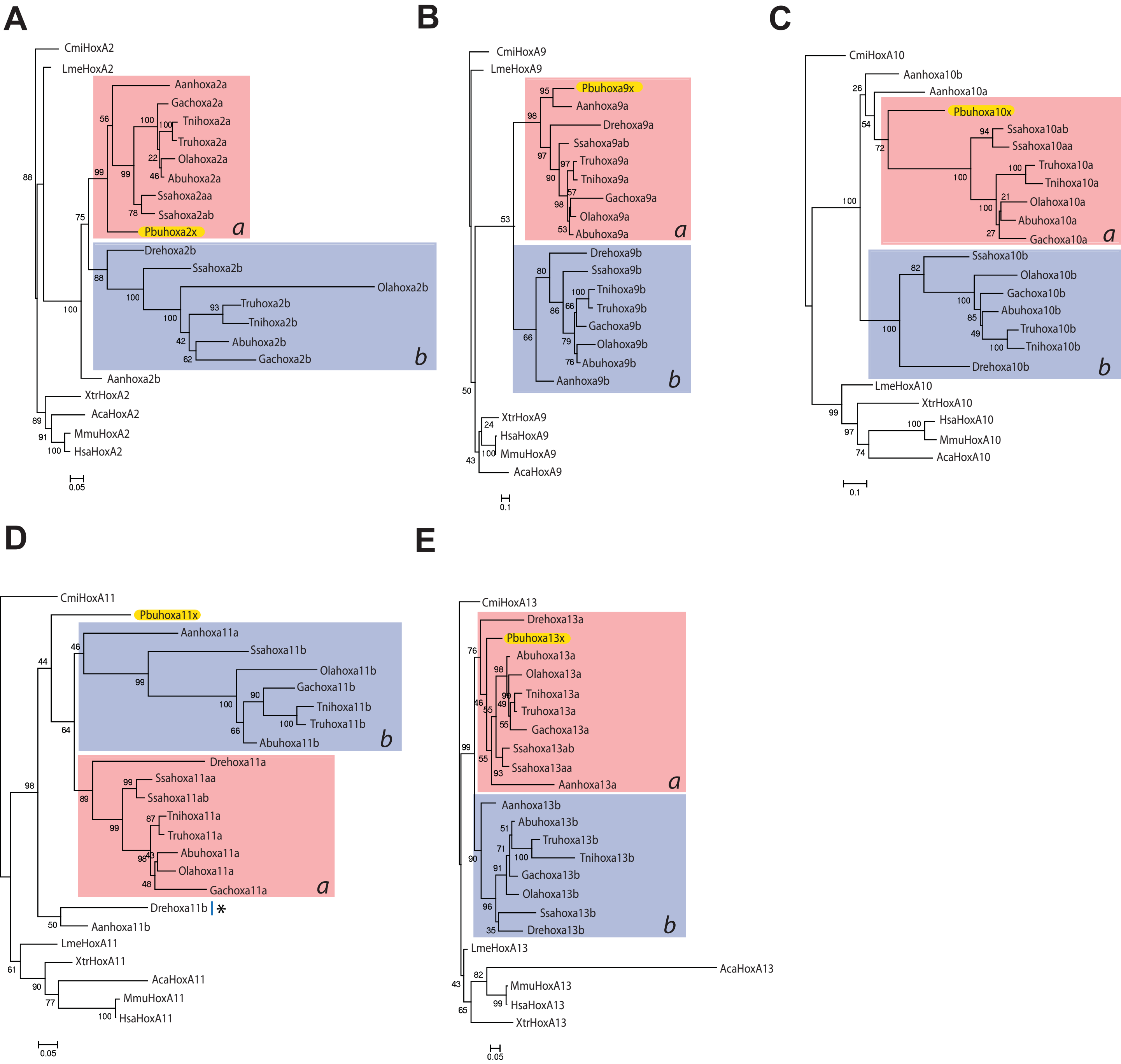

A
CmiHoxA2
LmeHoxA2
Aanhoxa2a
Gachoxa2a
Tnihoxa2a
Truhoxa2a
Olahoxa2a
Abuhoxa2a
Ssahoxa2aa
Ssahoxa2ab
Pbuhoxa2x
a
Drehoxa2b
Ssahoxa2b
Olahoxa2b
Truhoxa2b
Tnihoxa2b
Abuhoxa2b
Gachoxa2b
b
Aanhoxa2b
XtrHoxA2
AcaHoxA2
MmuHoxA2
HsaHoxA2
0.05
B
CmiHoxA9
LmeHoxA9
Pbuhoxa9x
Aanhoxa9a
Drehoxa9a
Ssahoxa9ab
Truhoxa9a
Tnihoxa9a
Gachoxa9a
Olahoxa9a
Abuhoxa9a
a
Drehoxa9b
Ssahoxa9b
Tnihoxa9b
Truhoxa9b
Gachoxa9b
Olahoxa9b
Abuhoxa9b
Aanhoxa9b
b
XtrHoxA9
HsaHoxA9
MmuHoxA9
AcaHoxA9
0.1
C
CmiHoxA10
Aanhoxa10b
Aanhoxa10a
Pbuhoxa10x
Ssahoxa10ab
Ssahoxa10aa
Truhoxa10a
Tnihoxa10a
Olahoxa10a
Abuhoxa10a
Gachoxa10a
a
Ssahoxa10b
Olahoxa10b
Gachoxa10b
Abuhoxa10b
Truhoxa10b
Tnihoxa10b
Drehoxa10b
b
LmeHoxA10
XtrHoxA10
HsaHoxA10
MmuHoxA10
AcaHoxA10
0.1
D
CmiHoxA11
Pbuhoxa11x
Aanhoxa11a
Ssahoxa11b
Olahoxa11b
Gachoxa11b
Tnihoxa11b
Truhoxa11b
Abuhoxa11b
b
Drehoxa11a
Ssahoxa11aa
Ssahoxa11ab
Tnihoxa11a
Truhoxa11a
Abuhoxa11a
Olahoxa11a
Gachoxa11a
a
Drehoxa11b
*
Aanhoxa11b
LmeHoxA11
XtrHoxA11
AcaHoxA11
MmuHoxA11
HsaHoxA11
0.05
E
CmiHoxA13
Drehoxa13a
Pbuhoxa13x
Abuhoxa13a
Olahoxa13a
Tnihoxa13a
Truhoxa13a
Gachoxa13a
Ssahoxa13ab
Ssahoxa13aa
Aanhoxa13a
a
Aanhoxa13b
Abuhoxa13b
Truhoxa13b
Tnihoxa13b
Gachoxa13b
Olahoxa13b
Ssahoxa13b
Drehoxa13b
b
LmeHoxA13
AcaHoxA13
MmuHoxA13
HsaHoxA13
XtrHoxA13
0.05

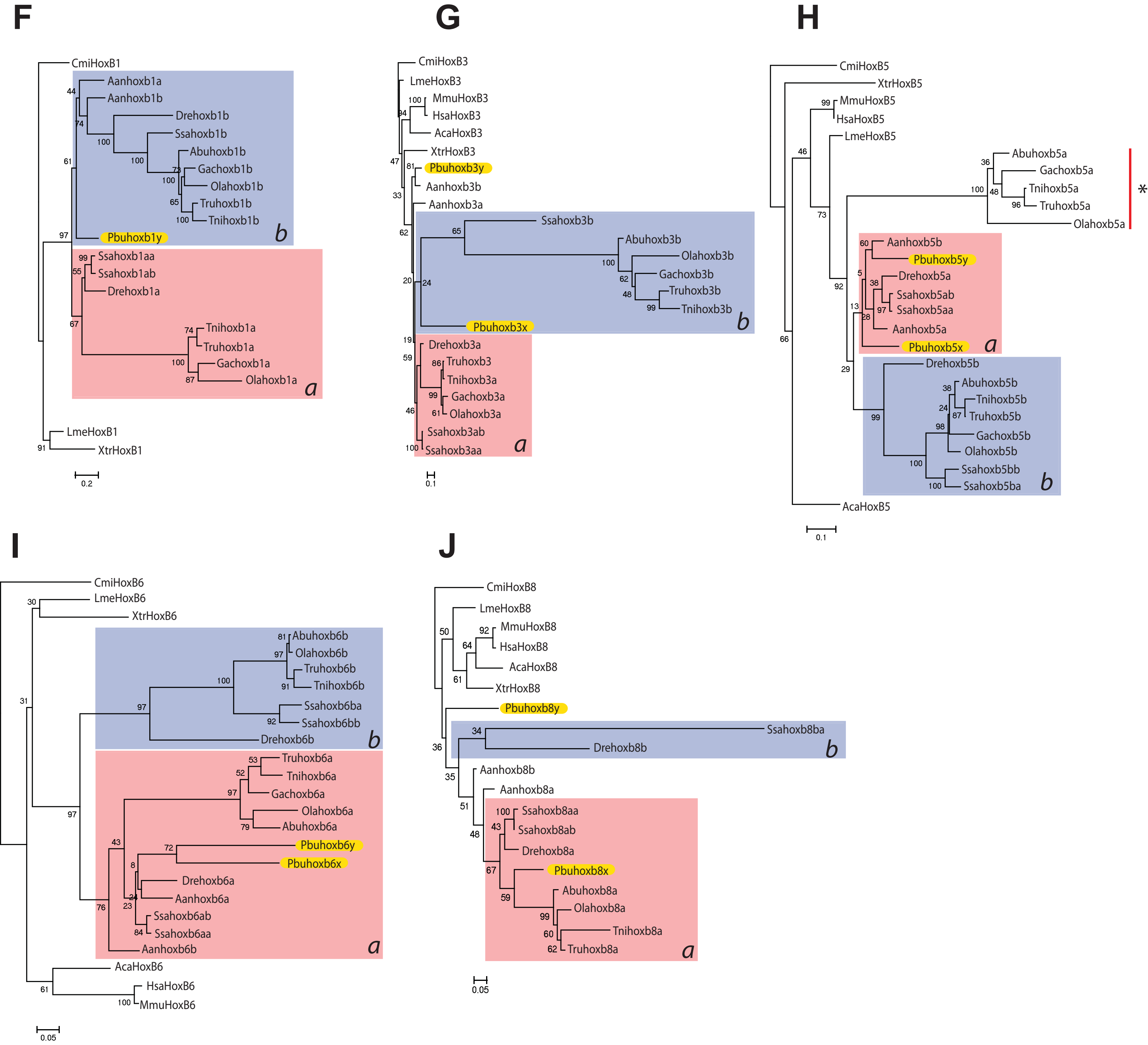
F
CmiHoxB1
Aanhoxb1a
Aanhoxb1b
Drehoxb1b
Ssahoxb1b
Abuhoxb1b
Gachoxb1b
Olahoxb1b
Truhoxb1b
Tnihoxb1b
Pbuhoxb1y
b
Ssahoxb1aa
Ssahoxb1ab
Drehoxb1a
Tnihoxb1a
Truhoxb1a
Gachoxb1a
Olahoxb1a
a
LmeHoxB1
XtrHoxB1
0.2
G
CmiHoxB3
LmeHoxB3
MmuHoxB3
HsaHoxB3
AcaHoxB3
XtrHoxB3
Pbuhoxb3y
Aanhoxb3b
Aanhoxb3a
Ssahoxb3b
Abuhoxb3b
Olahoxb3b
Gachoxb3b
Truhoxb3b
Tnihoxb3b
Pbuhoxb3x
b
Drehoxb3a
Truhoxb3
Tnihoxb3a
Gachoxb3a
Olahoxb3a
Ssahoxb3ab
Ssahoxb3aa
a
0.1
H
CmiHoxB5
XtrHoxB5
MmuHoxB5
HsaHoxB5
LmeHoxB5
Abuhoxb5a
Gachoxb5a
Tnihoxb5a
Truhoxb5a
Olahoxb5a
*
Aanhoxb5b
Pbuhoxb5y
Drehoxb5a
Ssahoxb5ab
Ssahoxb5aa
Aanhoxb5a
Pbuhoxb5x
a
Drehoxb5b
Abuhoxb5b
Tnihoxb5b
Truhoxb5b
Gachoxb5b
Olahoxb5b
Ssahoxb5bb
Ssahoxb5ba
b
AcaHoxB5
0.1
I
CmiHoxB6
LmeHoxB6
XtrHoxB6
Abuhoxb6b
Olahoxb6b
Truhoxb6b
Tnihoxb6b
Ssahoxb6ba
Ssahoxb6bb
Drehoxb6b
b
Truhoxb6a
Tnihoxb6a
Gachoxb6a
Olahoxb6a
Abuhoxb6a
Pbuhoxb6y
Pbuhoxb6x
Drehoxb6a
Aanhoxb6a
Ssahoxb6ab
Ssahoxb6aa
Aanhoxb6b
a
AcaHoxB6
HsaHoxB6
MmuHoxB6
0.05
J
CmiHoxB8
LmeHoxB8
MmuHoxB8
HsaHoxB8
AcaHoxB8
XtrHoxB8
Pbuhoxb8y
Ssahoxb8ba
Drehoxb8b
b
Aanhoxb8b
Aanhoxb8a
Ssahoxb8aa
Ssahoxb8ab
Drehoxb8a
Pbuhoxb8x
Abuhoxb8a
Olahoxb8a
Tnihoxb8a
Truhoxb8a
a
0.05

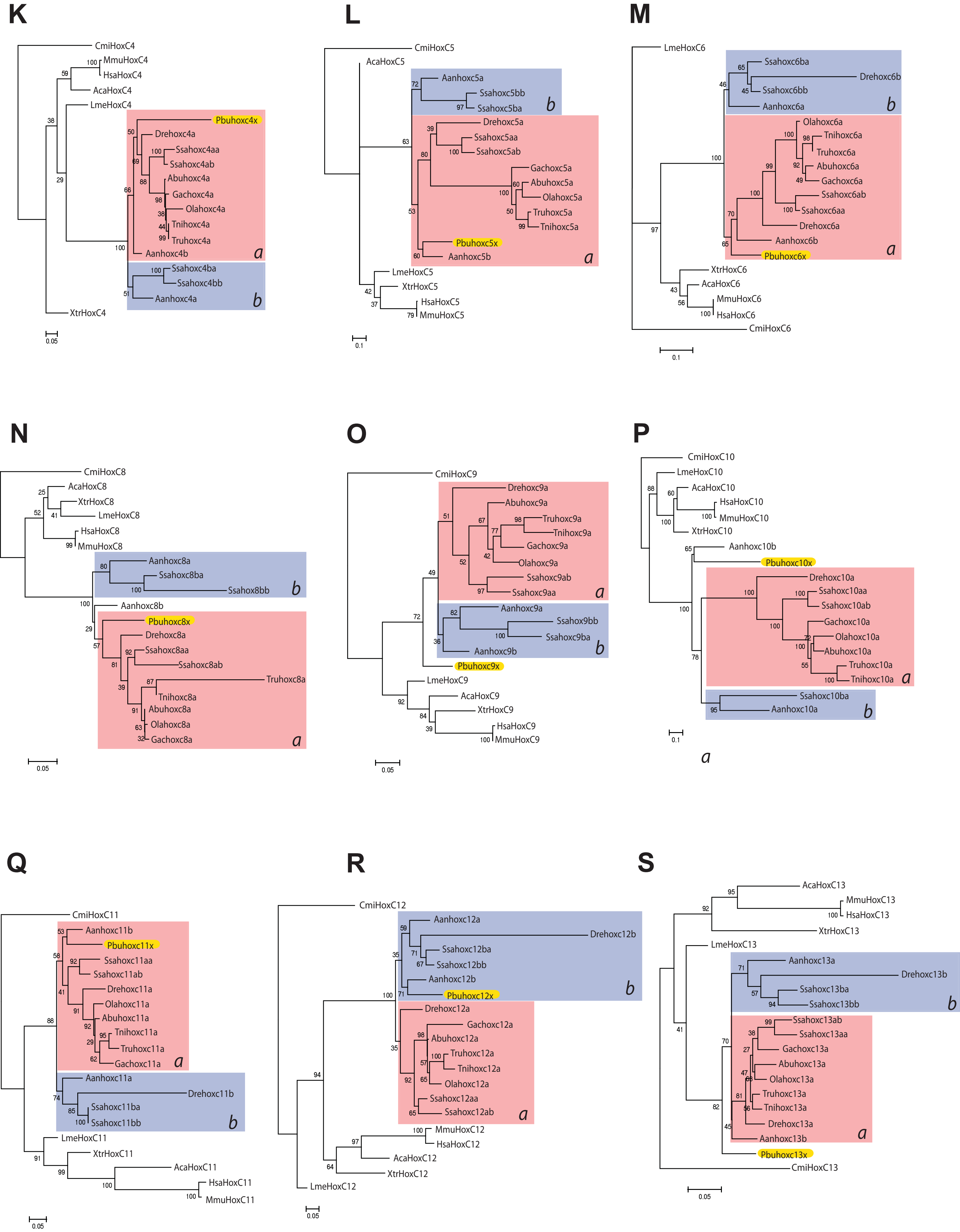
K
CmiHoxC4
MmuHoxC4
HsaHoxC4
AcaHoxC4
LmeHoxC4
Pbuhoxc4x
Drehoxc4a
Ssahoxc4aa
Ssahoxc4ab
Abuhoxc4a
Gachoxc4a
Olahoxc4a
Tnihoxc4a
Truhoxc4a
Aanhoxc4b
a
Ssahoxc4ba
Ssahoxc4bb
Aanhoxc4a
b
XtrHoxC4
0.05
L
CmiHoxC5
AcaHoxC5
Aanhoxc5a
Ssahoxc5bb
Ssahoxc5ba
b
Drehoxc5a
Ssahoxc5aa
Ssahoxc5ab
Gachoxc5a
Abuhoxc5a
Olahoxc5a
Truhoxc5a
Tnihoxc5a
Pbuhoxc5x
Aanhoxc5b
a
LmeHoxC5
XtrHoxC5
HsaHoxC5
MmuHoxC5
0.1
M
LmeHoxC6
Ssahoxc6ba
Drehoxc6b
Ssahoxc6bb
Aanhoxc6a
b
Olahoxc6a
Tnihoxc6a
Truhoxc6a
Abuhoxc6a
Gachoxc6a
Ssahoxc6ab
Ssahoxc6aa
Drehoxc6a
Aanhoxc6b
Pbuhoxc6x
a
XtrHoxC6
AcaHoxC6
MmuHoxC6
HsaHoxC6
CmiHoxC6
0.1
N
CmiHoxC8
AcaHoxC8
XtrHoxC8
LmeHoxC8
HsaHoxC8
MmuHoxC8
Aanhoxc8a
Ssahoxc8ba
Ssahox8bb
b
Aanhoxc8b
Pbuhoxc8x
Drehoxc8a
Ssahoxc8aa
Ssahoxc8ab
Truhoxc8a
Tnihoxc8a
Abuhoxc8a
Olahoxc8a
Gachoxc8a
a
0.05
O
CmiHoxC9
Drehoxc9a
Abuhoxc9a
Truhoxc9a
Tnihoxc9a
Gachoxc9a
Olahoxc9a
Ssahoxc9ab
Ssahoxc9aa
a
Aanhoxc9a
Ssahox9bb
Ssahoxc9ba
Aanhoxc9b
b
Pbuhoxc9x
LmeHoxC9
AcaHoxC9
XtrHoxC9
HsaHoxC9
MmuHoxC9
0.05
P
CmiHoxC10
LmeHoxC10
AcaHoxC10
HsaHoxC10
MmuHoxC10
XtrHoxC10
Aanhoxc10b
Pbuhoxc10x
Drehoxc10a
Ssahoxc10aa
Ssahoxc10ab
Gachoxc10a
Olahoxc10a
Abuhoxc10a
Truhoxc10a
Tnihoxc10a
a
Ssahoxc10ba
Aanhoxc10a
b
0.1
a
Q
CmiHoxC11
Aanhoxc11b
Pbuhoxc11x
Ssahoxc11aa
Ssahoxc11ab
Drehoxc11a
Olahoxc11a
Abuhoxc11a
Tnihoxc11a
Truhoxc11a
Gachoxc11a
a
Aanhoxc11a
Drehoxc11b
Ssahoxc11ba
Ssahoxc11bb
b
LmeHoxC11
XtrHoxC11
AcaHoxC11
HsaHoxC11
MmuHoxC11
0.05
R
CmiHoxC12
Aanhoxc12a
Drehoxc12b
Ssahoxc12ba
Ssahoxc12bb
Aanhoxc12b
Pbuhoxc12x
b
Drehoxc12a
Gachoxc12a
Abuhoxc12a
Truhoxc12a
Tnihoxc12a
Olahoxc12a
Ssahoxc12aa
Ssahoxc12ab
a
MmuHoxC12
HsaHoxC12
AcaHoxC12
XtrHoxC12
LmeHoxC12
0.05
S
AcaHoxC13
MmuHoxC13
HsaHoxC13
XtrHoxC13
LmeHoxC13
Aanhoxc13a
Drehoxc13b
Ssahoxc13ba
Ssahoxc13bb
b
Ssahoxc13ab
Ssahoxc13aa
Gachoxc13a
Abuhoxc13a
Olahoxc13a
Truhoxc13a
Tnihoxc13a
Drehoxc13a
Aanhoxc13b
a
Pbuhoxc13x
CmiHoxC13
0.05

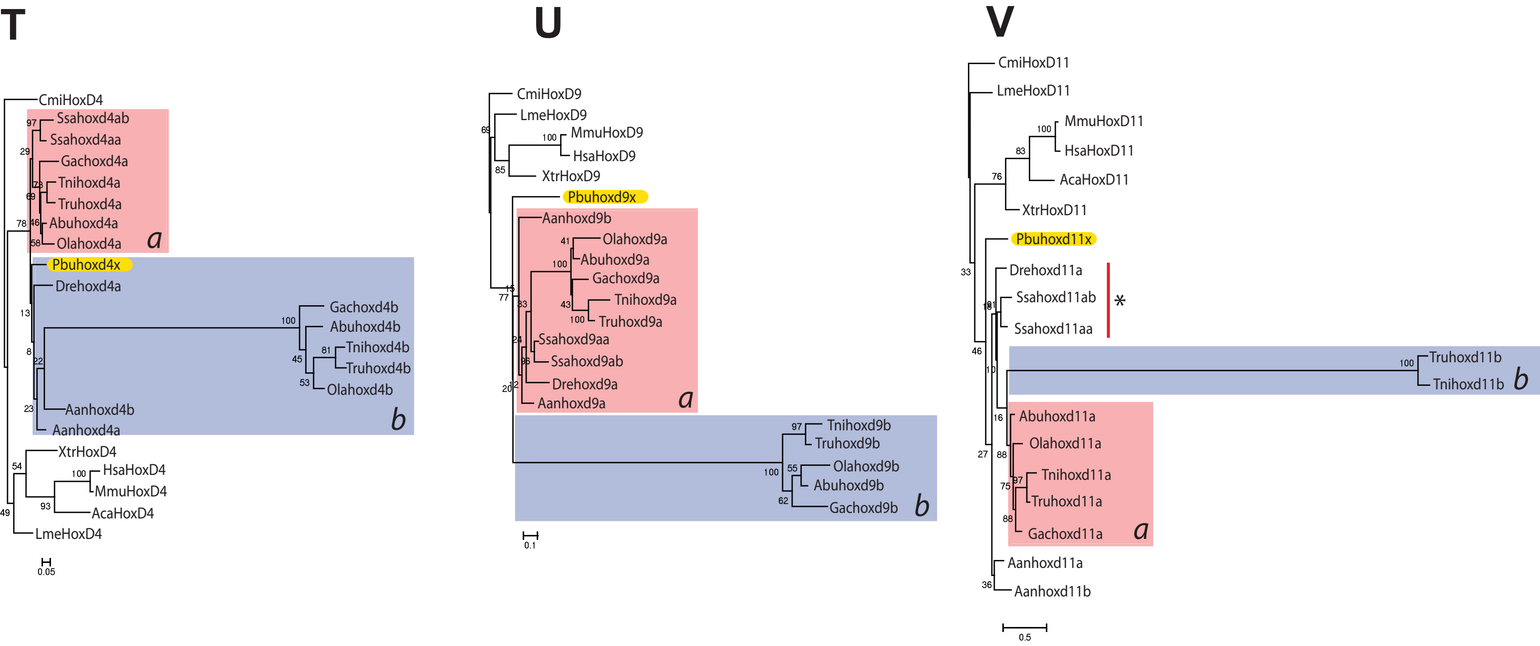
T
CmiHoxD4
Ssahoxd4ab
Ssahoxd4aa
Gachoxd4a
Tnihoxd4a
Truhoxd4a
Abuhoxd4a
Olahoxd4a
a
Pbuhoxd4x
Drehoxd4a
Gachoxd4b
Abuhoxd4b
Tnihoxd4b
Truhoxd4b
Olahoxd4b
Aanhoxd4b
Aanhoxd4a
b
XtrHoxD4
HsaHoxD4
MmuHoxD4
AcaHoxD4
LmeHoxD4
0.05
U
CmiHoxD9
LmeHoxD9
MmuHoxD9
HsaHoxD9
XtrHoxD9
Pbuhoxd9x
Aanhoxd9b
Olahoxd9a
Abuhoxd9a
Gachoxd9a
Tnihoxd9a
Truhoxd9a
Ssahoxd9aa
Ssahoxd9ab
Drehoxd9a
Aanhoxd9a
a
Tnihoxd9b
Truhoxd9b
Olahoxd9b
Abuhoxd9b
Gachoxd9b
b
0.1
V
CmiHoxD11
LmeHoxD11
MmuHoxD11
HsaHoxD11
AcaHoxD11
XtrHoxD11
Pbuhoxd11x
Drehoxd11a
Ssahoxd11ab
Ssahoxd11aa
*
Truhoxd11b
Tnihoxd11b
b
Abuhoxd11a
Olahoxd11a
Tnihoxd11a
Truhoxd11a
Gachoxd11a
a
Aanhoxd11a
Aanhoxd11b
0.5

Figure S1: Maximum likelihood *Hox* gene genealogies.

Maximum-Likelihood trees were built using with the program RAxML using the LG amino acid substitution model with gamma and invariant parameters and 1000 bootstrap replicates. Trees are shown for each orthology informative *Hox* gene family for which a *Pantodon* sequence was found. A: *HoxA2*, B: *HoxA9*, C: *HoxA10*, D: *HoxA11*, E: *HoxA13* F: *HoxB1*, G: *HoxB3*, H: *HoxB5*, I: *HoxB6*, J: *HoxB8* K: *HoxC4*, L: *HoxC5*, M: *HoxC6*, N: *HoxC8*, O: *HoxC9*, P: *HoxC10*, Q: *HoxC11*, R: *HoxC12*, S: *HoxC13*, T: *HoxD4*, U: *HoxD9*, V: *HoxD1*. The red boxes demarcate the post-TGD 'a' clades, while blue boxes mark is the post-TGD 'b' clades. Each clade is delineated at the most highly supported node inclusive of all clupeocephalan teleost 'a' or 'b' genes and the *Pantodon* gene where applicable, and may or may not include *Anguilla* sequences. Atypically paraphyletic clupeocephalan post-TGD 'a' or 'b' clades are demarcated with an asterisk (*). Bootstrap support values are shown above each node. *Pantodon* sequences are displayed in bold and highlighted in yellow. Species names are abbreviated are as follows. Cmi: *Callorhinchus milii*, Lme: *Latimeria menadoensis*, Xtr: *Xenopus tropicalis*, Aca: *Anolis caroliensis*, Mmu: *Mus musculus*, Hsa: *Homo sapiens*, Aan: *Anguilla anguilla*, Pbu: *Pantodon buchholzi*, Dre: *Danio rerio*, Ssa: *Salmo salar*, Ola: *Oryzias latipes*, Gac: *Gasterosteus aculeatus*, Abu: *Astatotilapia burtoni*, Tru: *Takifugu rubripes*, Tni: *Tetraodon nigroviridis*.

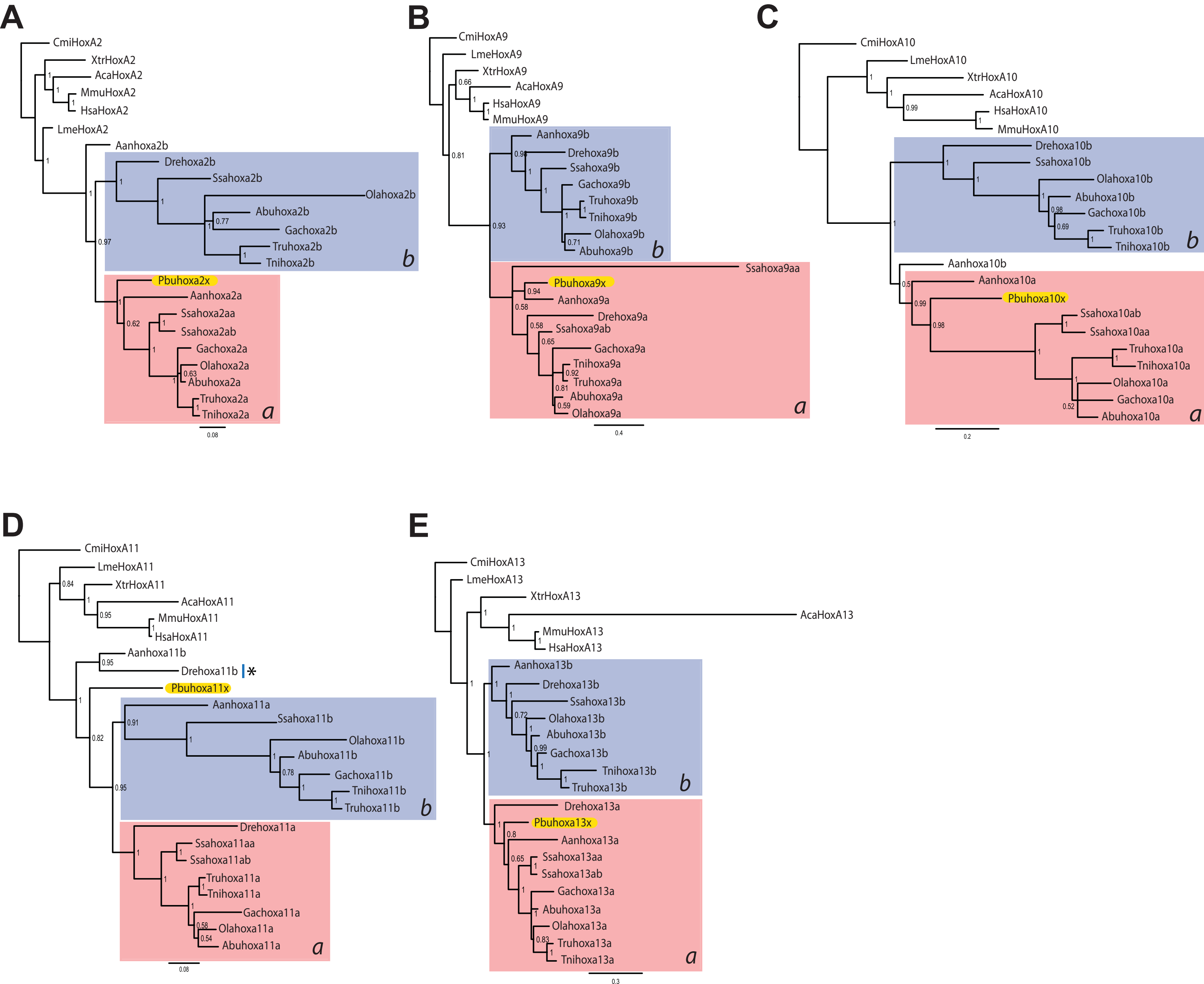
A
CmiHoxA2
XtrHoxA2
AcaHoxA2
MmuHoxA2
HsaHoxA2
LmeHoxA2
Aanhoxa2b
Drehoxa2b
Ssahoxa2b
Olahoxa2b
Abuhoxa2b
Gachoxa2b
Truhoxa2b
Tnihoxa2b
b
Pbuhoxa2x
Aanhoxa2a
Ssahoxa2aa
Ssahoxa2ab
Gachoxa2a
Olahoxa2a
Abuhoxa2a
Truhoxa2a
Tnihoxa2a
a
0.08
B
CmiHoxA9
LmeHoxA9
XtrHoxA9
AcaHoxA9
HsaHoxA9
MmuHoxA9
Aanhoxa9b
Drehoxa9b
Ssahoxa9b
Gachoxa9b
Truhoxa9b
Tnihoxa9b
Olahoxa9b
Abuhoxa9b
b
Ssahoxa9aa
Pbuhoxa9x
Aanhoxa9a
Drehoxa9a
Ssahoxa9ab
Gachoxa9a
Tnihoxa9a
Truhoxa9a
Abuhoxa9a
Olahoxa9a
a
0.4
C
CmiHoxA10
LmeHoxA10
XtrHoxA10
AcaHoxA10
HsaHoxA10
MmuHoxA10
Drehoxa10b
Ssahoxa10b
Olahoxa10b
Abuhoxa10b
Gachoxa10b
Truhoxa10b
Tnihoxa10b
b
Aanhoxa10b
Aanhoxa10a
Pbuhoxa10x
Ssahoxa10ab
Ssahoxa10aa
Truhoxa10a
Tnihoxa10a
Olahoxa10a
Gachoxa10a
Abuhoxa10a
a
0.2
D
CmiHoxA11
LmeHoxA11
XtrHoxA11
AcaHoxA11
MmuHoxA11
HsaHoxA11
Aanhoxa11b
Drehoxa11b *
Pbuhoxa11x
Aanhoxa11a
Ssahoxa11b
Olahoxa11b
Abuhoxa11b
Gachoxa11b
Tnihoxa11b
Truhoxa11b
b
Drehoxa11a
Ssahoxa11aa
Ssahoxa11ab
Truhoxa11a
Tnihoxa11a
Gachoxa11a
Olahoxa11a
Abuhoxa11a
a
0.08
E
CmiHoxA13
LmeHoxA13
XtrHoxA13
AcaHoxA13
MmuHoxA13
HsaHoxA13
Aanhoxa13b
Drehoxa13b
Ssahoxa13b
Olahoxa13b
Abuhoxa13b
Gachoxa13b
Tnihoxa13b
Truhoxa13b
b
Drehoxa13a
Pbuhoxa13x
Aanhoxa13a
Ssahoxa13aa
Ssahoxa13ab
Gachoxa13a
Abuhoxa13a
Olahoxa13a
Truhoxa13a
Tnihoxa13a
a
0.3

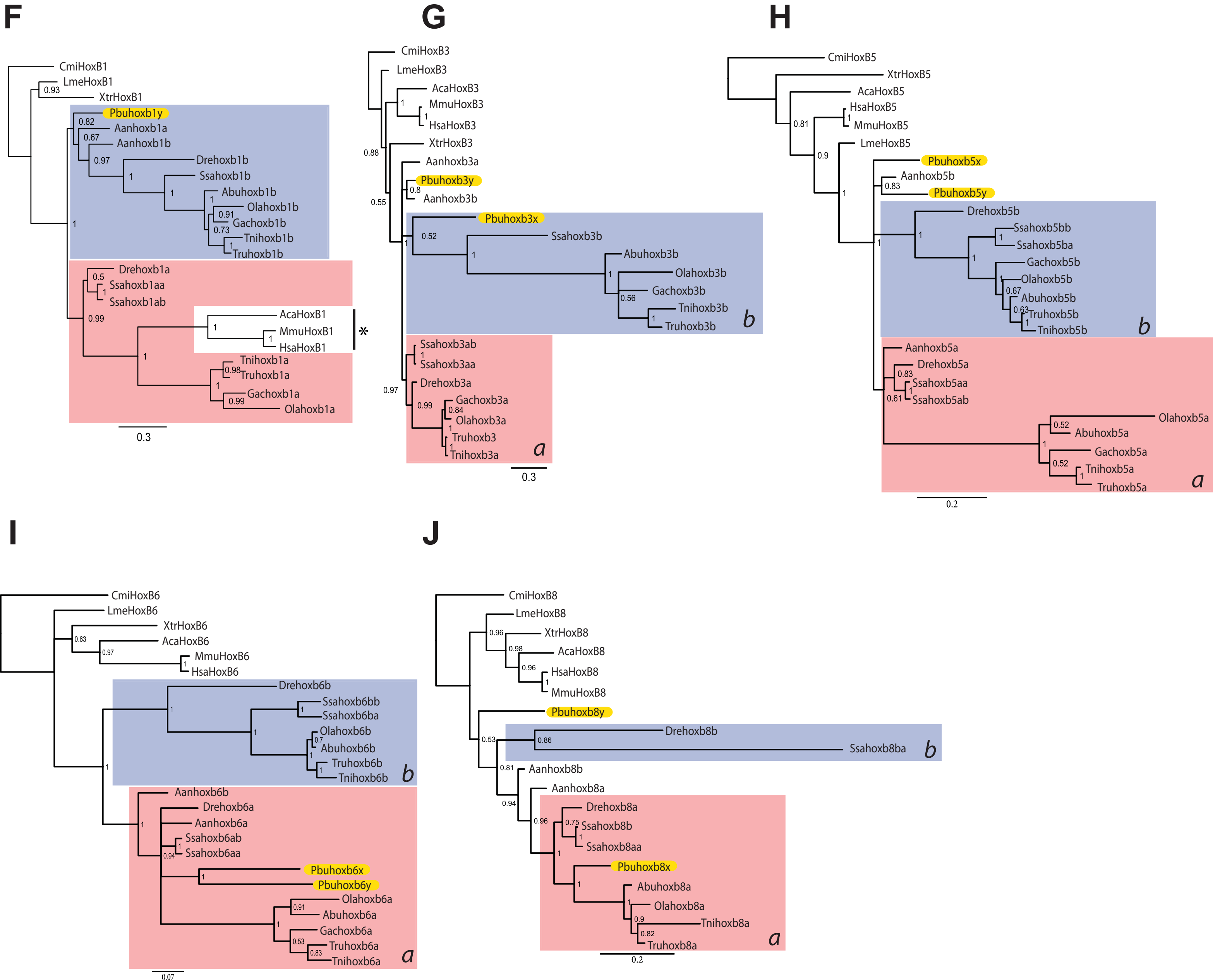
F
CmiHoxB1
LmeHoxB1
XtrHoxB1
Pbuhoxb1y
Aanhoxb1a
Aanhoxb1b
Drehoxb1b
Ssahoxb1b
Abuhoxb1b
Olahoxb1b
Gachoxb1b
Tnihoxb1b
Truhoxb1b
Drehoxb1a
Ssahoxb1aa
Ssahoxb1ab
AcaHoxB1
MmuHoxB1
HsaHoxB1
*
Tnihoxb1a
Truhoxb1a
Gachoxb1a
Olahoxb1a
0.3
G
CmiHoxB3
LmeHoxB3
AcaHoxB3
MmuHoxB3
HsaHoxB3
XtrHoxB3
Aanhoxb3a
Pbuhoxb3y
Aanhoxb3b
Pbuhoxb3x
Ssahoxb3b
Abuhoxb3b
Olahoxb3b
Gachoxb3b
Tnihoxb3b
Truhoxb3b
b
Ssahoxb3ab
Ssahoxb3aa
Drehoxb3a
Gachoxb3a
Olahoxb3a
Truhoxb3
Tnihoxb3a
a
0.3
H
CmiHoxB5
XtrHoxB5
AcaHoxB5
HsaHoxB5
MmuHoxB5
LmeHoxB5
Pbuhoxb5x
Aanhoxb5b
Pbuhoxb5y
Drehoxb5b
Ssahoxb5bb
Ssahoxb5ba
Gachoxb5b
Olahoxb5b
Abuhoxb5b
Truhoxb5b
Tnihoxb5b
b
Aanhoxb5a
Drehoxb5a
Ssahoxb5aa
Ssahoxb5ab
Olahoxb5a
Abuhoxb5a
Gachoxb5a
Tnihoxb5a
Truhoxb5a
a
0.2
I
CmiHoxB6
LmeHoxB6
XtrHoxB6
AcaHoxB6
MmuHoxB6
HsaHoxB6
Drehoxb6b
Ssahoxb6bb
Ssahoxb6ba
Olahoxb6b
Abuhoxb6b
Truhoxb6b
Tnihoxb6b
b
Aanhoxb6b
Drehoxb6a
Aanhoxb6a
Ssahoxb6ab
Ssahoxb6aa
Pbuhoxb6x
Pbuhoxb6y
Olahoxb6a
Abuhoxb6a
Gachoxb6a
Truhoxb6a
Tnihoxb6a
a
0.07
J
CmiHoxB8
LmeHoxB8
XtrHoxB8
AcaHoxB8
HsaHoxB8
MmuHoxB8
Pbuhoxb8y
Drehoxb8b
Ssahoxb8ba
b
Aanhoxb8b
Aanhoxb8a
Drehoxb8a
Ssahoxb8b
Ssahoxb8aa
Pbuhoxb8x
Abuhoxb8a
Olahoxb8a
Tnihoxb8a
Truhoxb8a
a
0.2

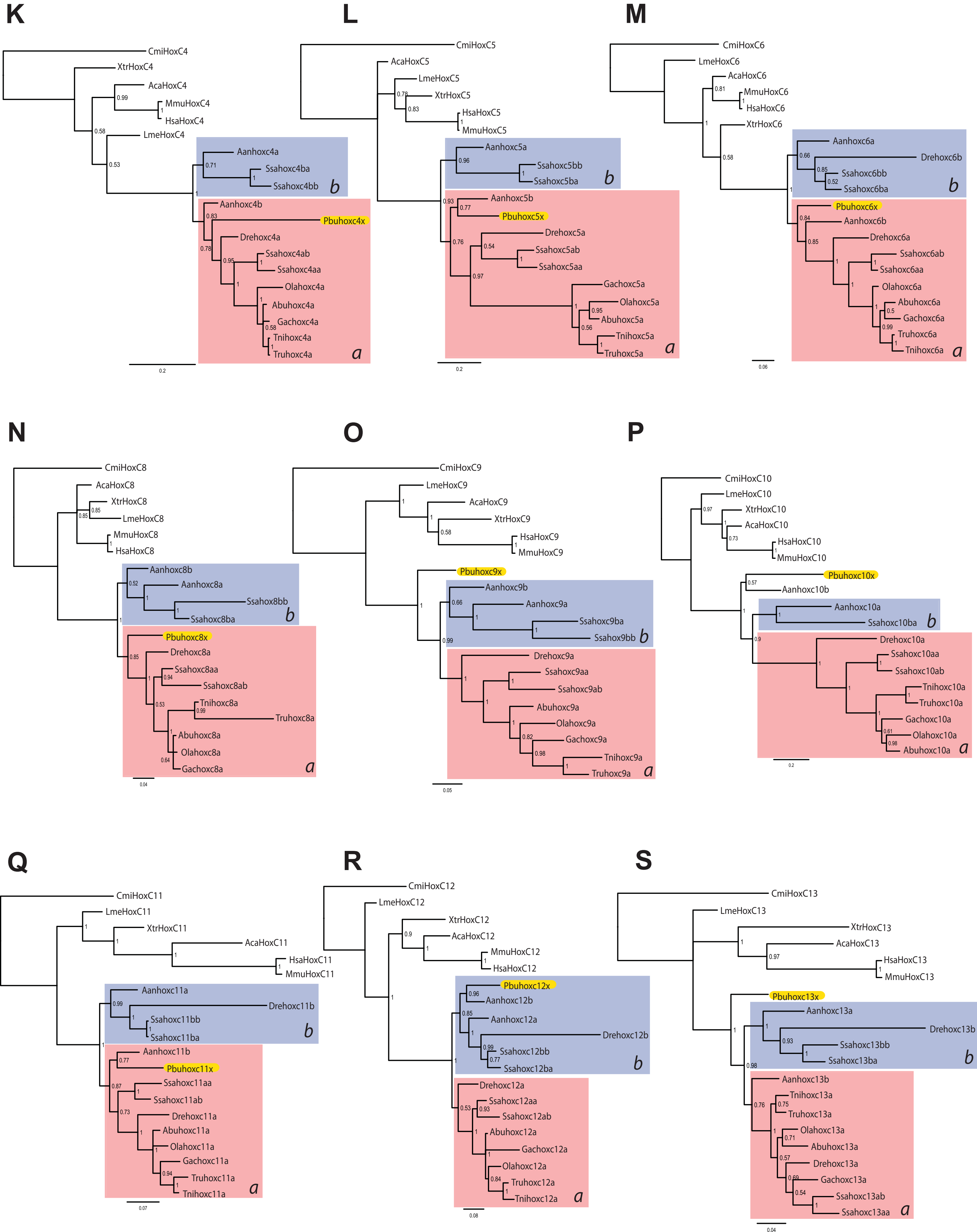

K
CmiHoxC4
XtrHoxC4
AcaHoxC4
MmuHoxC4
HsaHoxC4
LmeHoxC4
Aanhoxc4a
Ssahoxc4ba
Ssahoxc4bb
b
Aanhoxc4b
Pbuhoxc4x
Drehoxc4a
Ssahoxc4ab
Ssahoxc4aa
Olahoxc4a
Abuhoxc4a
Gachoxc4a
Tnihoxc4a
Truhoxc4a
a
0.2
L
CmiHoxC5
AcaHoxC5
LmeHoxC5
XtrHoxC5
HsaHoxC5
MmuHoxC5
Aanhoxc5a
Ssahoxc5bb
Ssahoxc5ba
b
Aanhoxc5b
Pbuhoxc5x
Drehoxc5a
Ssahoxc5ab
Ssahoxc5aa
Gachoxc5a
Olahoxc5a
Abuhoxc5a
Tnihoxc5a
Truhoxc5a
a
0.2
M
CmiHoxC6
LmeHoxC6
AcaHoxC6
MmuHoxC6
HsaHoxC6
XtrHoxC6
Aanhoxc6a
Drehoxc6b
Ssahoxc6bb
Ssahoxc6ba
b
Pbuhoxc6x
Aanhoxc6b
Drehoxc6a
Ssahoxc6ab
Ssahoxc6aa
Olahoxc6a
Abuhoxc6a
Gachoxc6a
Truhoxc6a
Tnihoxc6a
a
0.06
N
CmiHoxC8
AcaHoxC8
XtrHoxC8
LmeHoxC8
MmuHoxC8
HsaHoxC8
Aanhoxc8b
Aanhoxc8a
Ssahox8bb
Ssahoxc8ba
b
Pbuhoxc8x
Drehoxc8a
Ssahoxc8aa
Ssahoxc8ab
Tnihoxc8a
Truhoxc8a
Abuhoxc8a
Olahoxc8a
Gachoxc8a
a
0.04
O
CmiHoxC9
LmeHoxC9
AcaHoxC9
XtrHoxC9
HsaHoxC9
MmuHoxC9
Pbuhoxc9x
Aanhoxc9b
Aanhoxc9a
Ssahoxc9ba
Ssahox9bb
b
Drehoxc9a
Ssahoxc9aa
Ssahoxc9ab
Abuhoxc9a
Olahoxc9a
Gachoxc9a
Tnihoxc9a
Truhoxc9a
a
0.05
P
CmiHoxC10
LmeHoxC10
XtrHoxC10
AcaHoxC10
HsaHoxC10
MmuHoxC10
Pbuhoxc10x
Aanhoxc10b
Aanhoxc10a
Ssahoxc10ba
b
Drehoxc10a
Ssahoxc10aa
Ssahoxc10ab
Tnihoxc10a
Truhoxc10a
Gachoxc10a
Olahoxc10a
Abuhoxc10a
a
0.2
Q
CmiHoxC11
LmeHoxC11
XtrHoxC11
AcaHoxC11
HsaHoxC11
MmuHoxC11
Aanhoxc11a
Drehoxc11b
Ssahoxc11bb
Ssahoxc11ba
b
Aanhoxc11b
Pbuhoxc11x
Ssahoxc11aa
Ssahoxc11ab
Drehoxc11a
Abuhoxc11a
Olahoxc11a
Gachoxc11a
Truhoxc11a
Tnihoxc11a
a
0.07
R
CmiHoxC12
LmeHoxC12
XtrHoxC12
AcaHoxC12
MmuHoxC12
HsaHoxC12
Pbuhoxc12x
Aanhoxc12b
Aanhoxc12a
Drehoxc12b
Ssahoxc12bb
Ssahoxc12ba
b
Drehoxc12a
Ssahoxc12aa
Ssahoxc12ab
Abuhoxc12a
Gachoxc12a
Olahoxc12a
Truhoxc12a
Tnihoxc12a
a
0.08
S
CmiHoxC13
LmeHoxC13
XtrHoxC13
AcaHoxC13
HsaHoxC13
MmuHoxC13
Pbuhoxc13x
Aanhoxc13a
Drehoxc13b
Ssahoxc13bb
Ssahoxc13ba
b
Aanhoxc13b
Tnihoxc13a
Truhoxc13a
Olahoxc13a
Abuhoxc13a
Drehoxc13a
Gachoxc13a
Ssahoxc13ab
Ssahoxc13aa
a
0.04

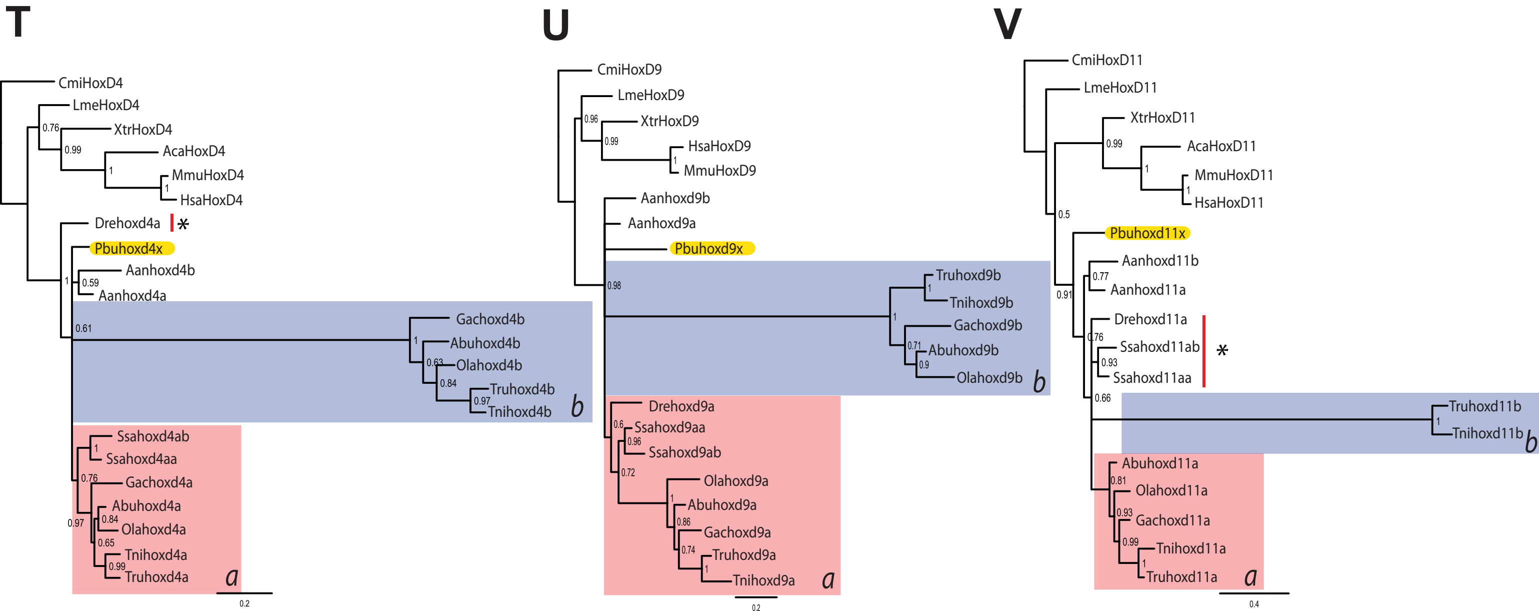
T
CmiHoxD4
LmeHoxD4
XtrHoxD4
AcaHoxD4
MmuHoxD4
HsaHoxD4
Drehoxd4a
*
Pbuhoxd4x
Aanhoxd4b
Aanhoxd4a
Gachoxd4b
Abuhoxd4b
Olahoxd4b
Truhoxd4b
Tnihoxd4b
b
Ssahoxd4ab
Ssahoxd4aa
Gachoxd4a
Abuhoxd4a
Olahoxd4a
Tnihoxd4a
Truhoxd4a
a
0.2
U
CmiHoxD9
LmeHoxD9
XtrHoxD9
HsaHoxD9
MmuHoxD9
Aanhoxd9b
Aanhoxd9a
Pbuhoxd9x
Truhoxd9b
Tnihoxd9b
Gachoxd9b
Abuhoxd9b
Olahoxd9b
b
Drehoxd9a
Ssahoxd9aa
Ssahoxd9ab
Olahoxd9a
Abuhoxd9a
Gachoxd9a
Truhoxd9a
Tnihoxd9a
a
0.2
V
CmiHoxD11
LmeHoxD11
XtrHoxD11
AcaHoxD11
MmuHoxD11
HsaHoxD11
Pbuhoxd11x
Aanhoxd11b
Aanhoxd11a
Drehoxd11a
Ssahoxd11ab
Ssahoxd11aa
*
Truhoxd11b
Tnihoxd11b
b
Abuhoxd11a
Olahoxd11a
Gachoxd11a
Tnihoxd11a
Truhoxd11a
a
0.4

Figure S2: Bayesian *Hox* gene genealogies.

Bayesian trees were built using with the program mrbayes using the WAG amino acid substitution model with gamma and invariant parameters and run over 1000000 generations. Trees are shown for each orthology informative *Hox* gene family for which a *Pantodon* sequence was found. A: *HoxA2*, B: *HoxA9*, C: *HoxA10*, D: *HoxA11*, E: *HoxA13* F: *HoxB1*, G: *HoxB3*, H: *HoxB5*, I: *HoxB6*, J: *HoxB8* K: *HoxC4*, L: *HoxC5*, M: *HoxC6*, N: *HoxC8*, O: *HoxC9*, P: *HoxC10*, Q: *HoxC11*, R: *HoxC12*, S: *HoxC13*, T: *HoxD4*, U: *HoxD9*, V: *HoxD1*. The red boxes demarcate the post-TGD 'a' clades, while blue boxes mark is the post-TGD 'b' clades. Each clade is delineated at the most highly supported node inclusive of all clupeocephalan teleost 'a' or 'b' genes and the *Pantodon* gene where applicable, and may or may not include *Anguilla* sequences. Atypically paraphyletic clupeocephalan post-TGD 'a' or 'b' clades are demarcated with an asterisk (*). Posterior probabilities are shown above each node. *Pantodon* sequences are displayed in bold and highlighted in yellow. Species names are abbreviated are as follows. Cmi: *Callorhinchus milii*, Lme: *Latimeria menadoensis*, Xtr: *Xenopus tropicalis*, Aca: *Anolis caroliensis*, Mmu: *Mus musculus*, Hsa: *Homo sapiens*, Aan: *Anguilla anguilla*, Pbu: *Pantodon buchholzi*, Dre: *Danio rerio*, Ssa: *Salmo salar*, Ola: *Oryzias latipes*, Gac: *Gasterosteus aculeatus*, Abu: *Astatotilapia burtoni*, Tru: *Takifugu rubripes*, Tni: *Tetraodon nigroviridis*.
