# Supplementary material for: Enigmatic Orthology Relationships between Hox Clusters of the African Butterfly Fish and Other Teleosts Following Ancient Whole-Genome Duplication
Source: Mol Biol Evol. 2014 Jun 27;31(10):2592–611. doi: 10.1093/molbev/msu202 (PMC4166920; doi:10.1093/molbev/msu202)
Supplement: Supplementary Data [file supp_msu202_msu202_SI_FigS3_VISTA_alignments_FigS4_CNE_histogram-KM.pdf]

Cmi_HoxA|FJ824598|1-286881

HoxA VISTA alignment with *C. milli* baseline

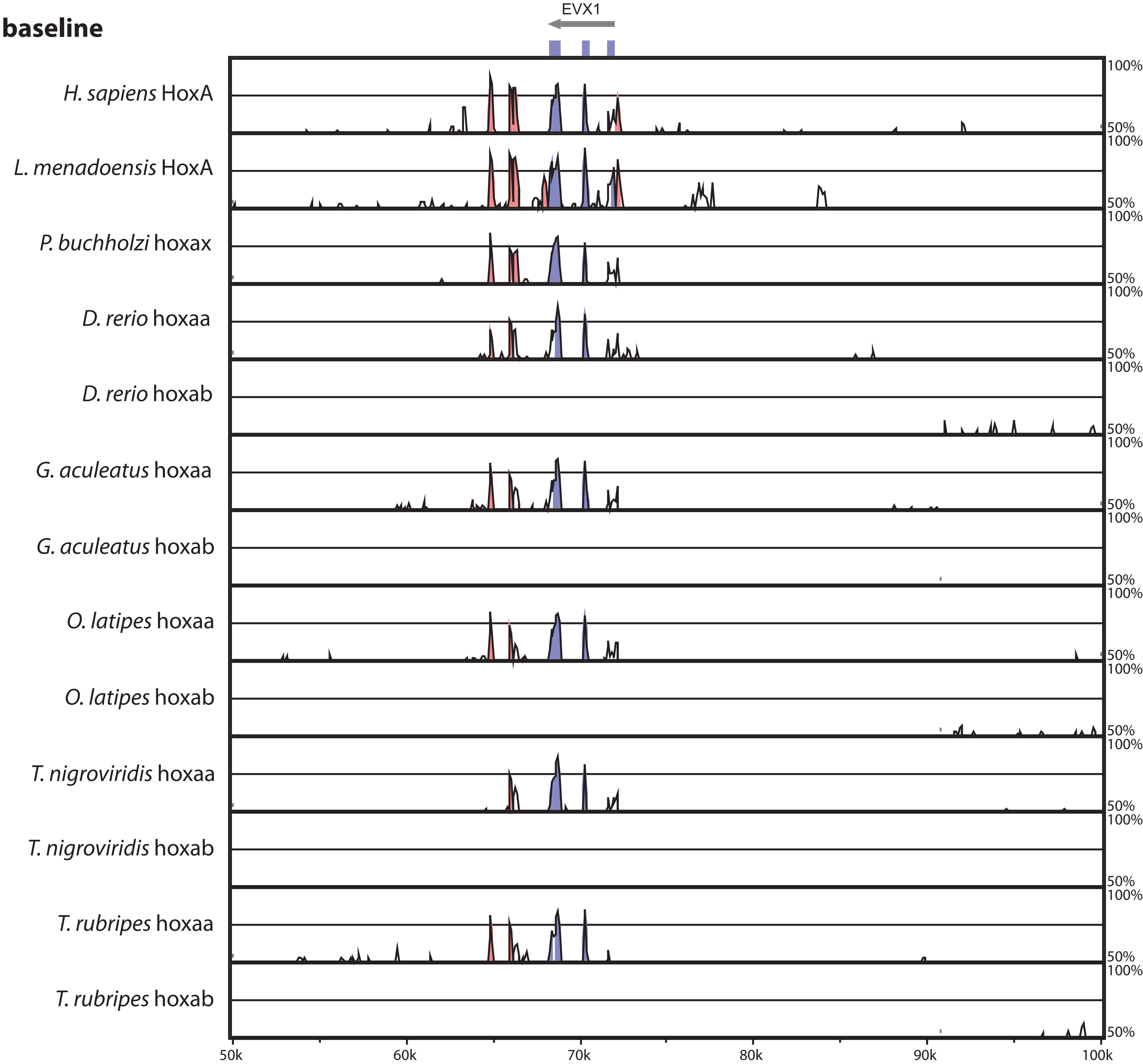

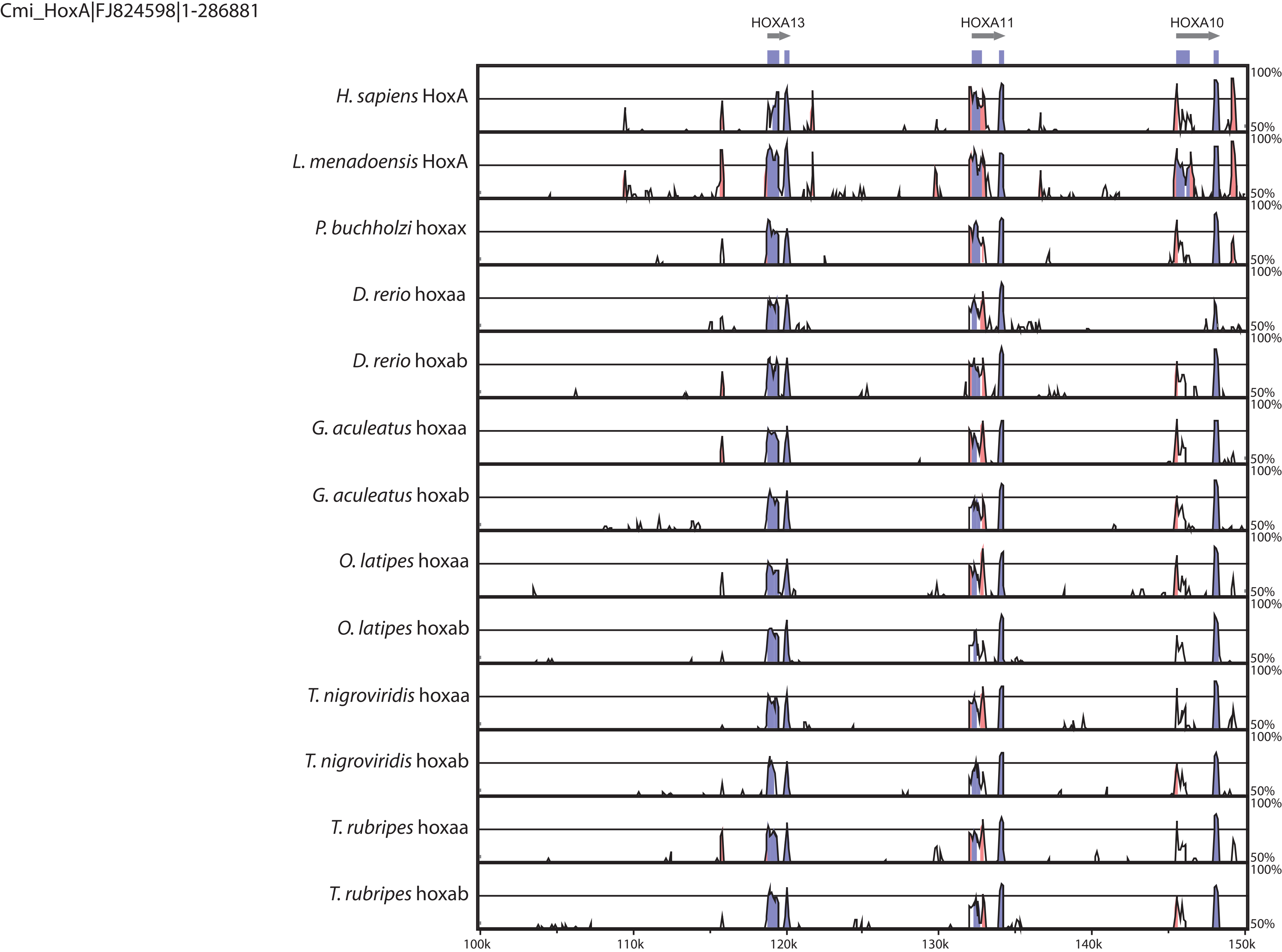

Cmi_HoxA|FJ824598|1-286881
HOXA13
HOXA11
HOXA10
H. sapiens HoxA
L. menadoensis HoxA
P. buchholzi hoxax
D. rerio hoxaa
D. rerio hoxab
G. aculeatus hoxaa
G. aculeatus hoxab
O. latipes hoxaa
O. latipes hoxab
T. nigroviridis hoxaa
T. nigroviridis hoxab
T. rubripes hoxaa
T. rubripes hoxab
100%
50%
100k
110k
120k
130k
140k
150k

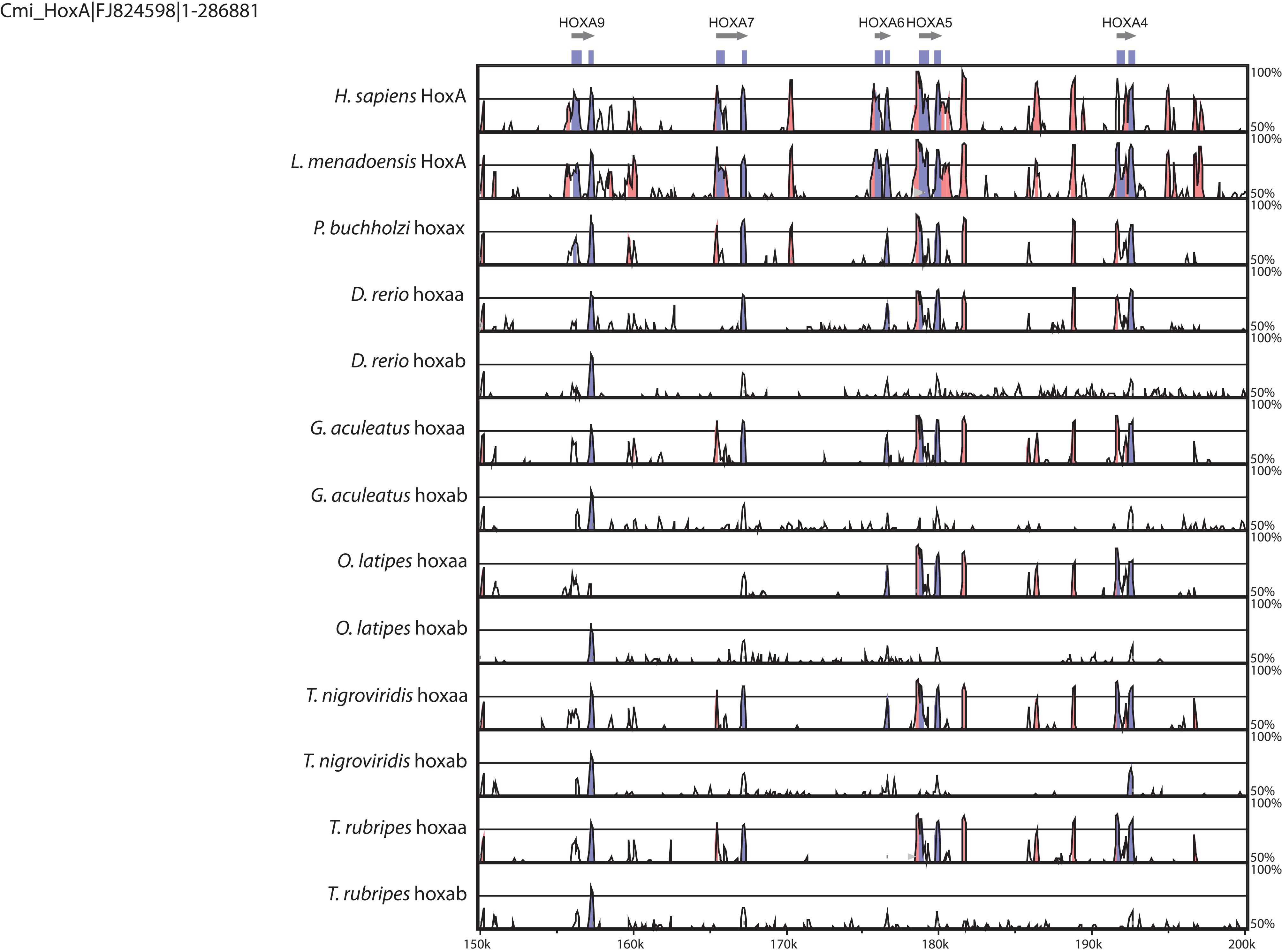
Cmi_HoxA|FJ824598|1-286881
HOXA9
HOXA7
HOXA6
HOXA5
HOXA4
H. sapiens HoxA
L. menadoensis HoxA
P. buchholzi hoxax
D. rerio hoxaa
D. rerio hoxab
G. aculeatus hoxaa
G. aculeatus hoxab
O. latipes hoxaa
O. latipes hoxab
T. nigroviridis hoxaa
T. nigroviridis hoxab
T. rubripes hoxaa
T. rubripes hoxab
100%
50%
150k
160k
170k
180k
190k
200k

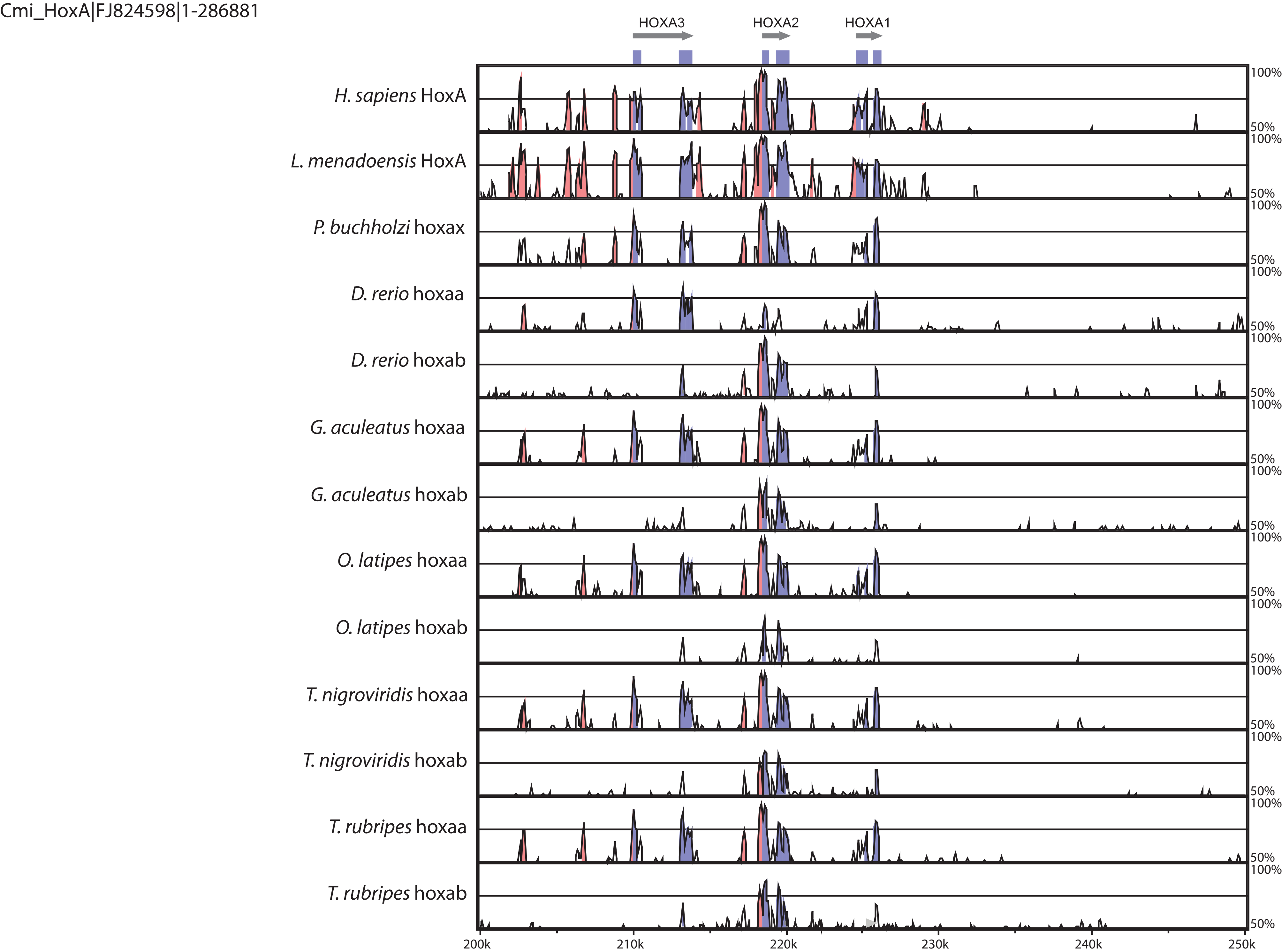

Cmi_HoxA|FJ824598|1-286881
HOXA3
HOXA2
HOXA1
H. sapiens HoxA
L. menadoensis HoxA
P. buchholzi hoxax
D. rerio hoxaa
D. rerio hoxab
G. aculeatus hoxaa
G. aculeatus hoxab
O. latipes hoxaa
O. latipes hoxab
T. nigroviridis hoxaa
T. nigroviridis hoxab
T. rubripes hoxaa
T. rubripes hoxab
100%
50%
200k
210k
220k
230k
240k
250k

Cmi_HoxB|FJ824599.1|1-368325

HoxB VISTA alignment with *C. milli* baseline

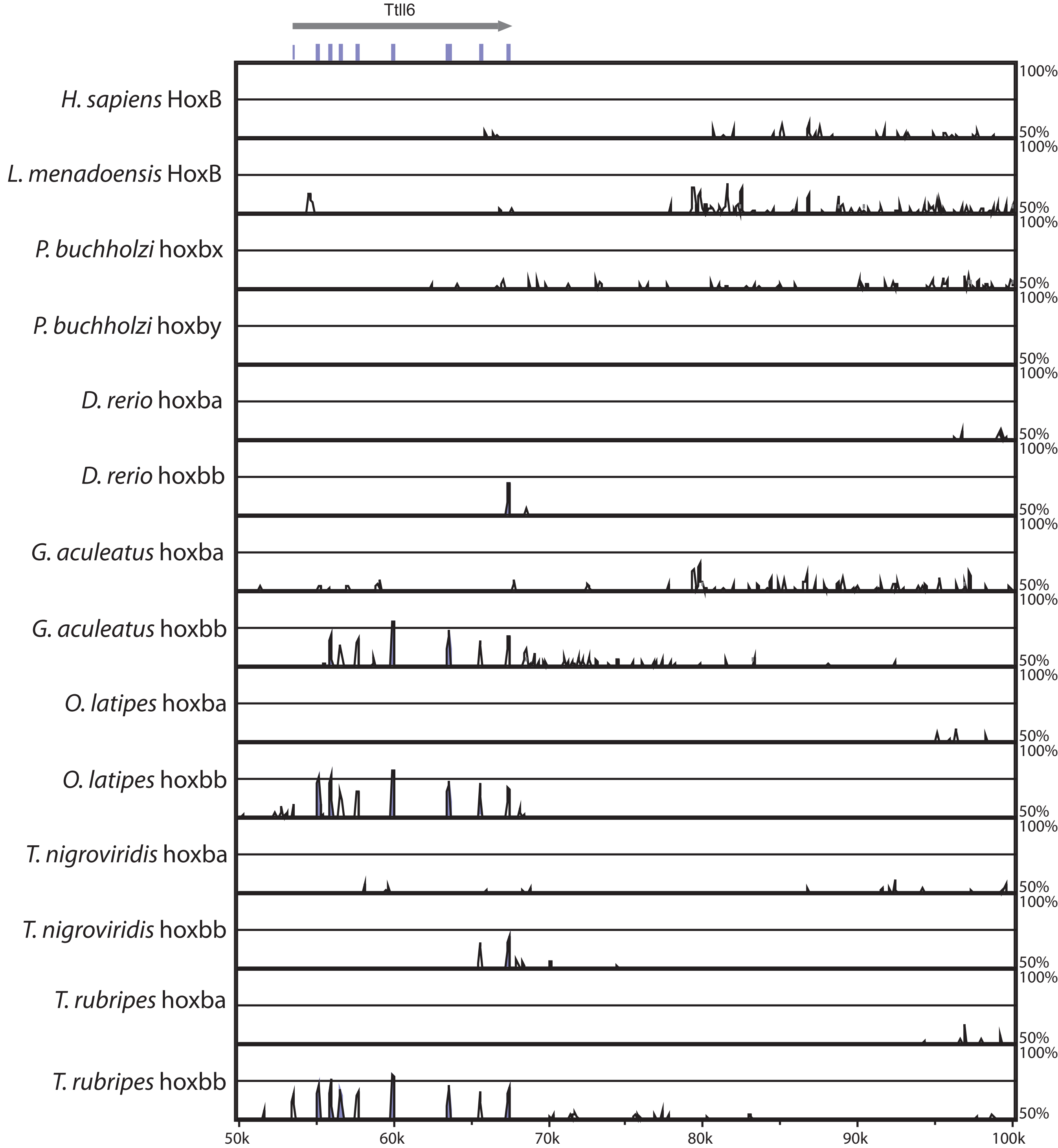

Cmi_HoxB|FJ824599.1|1-368325

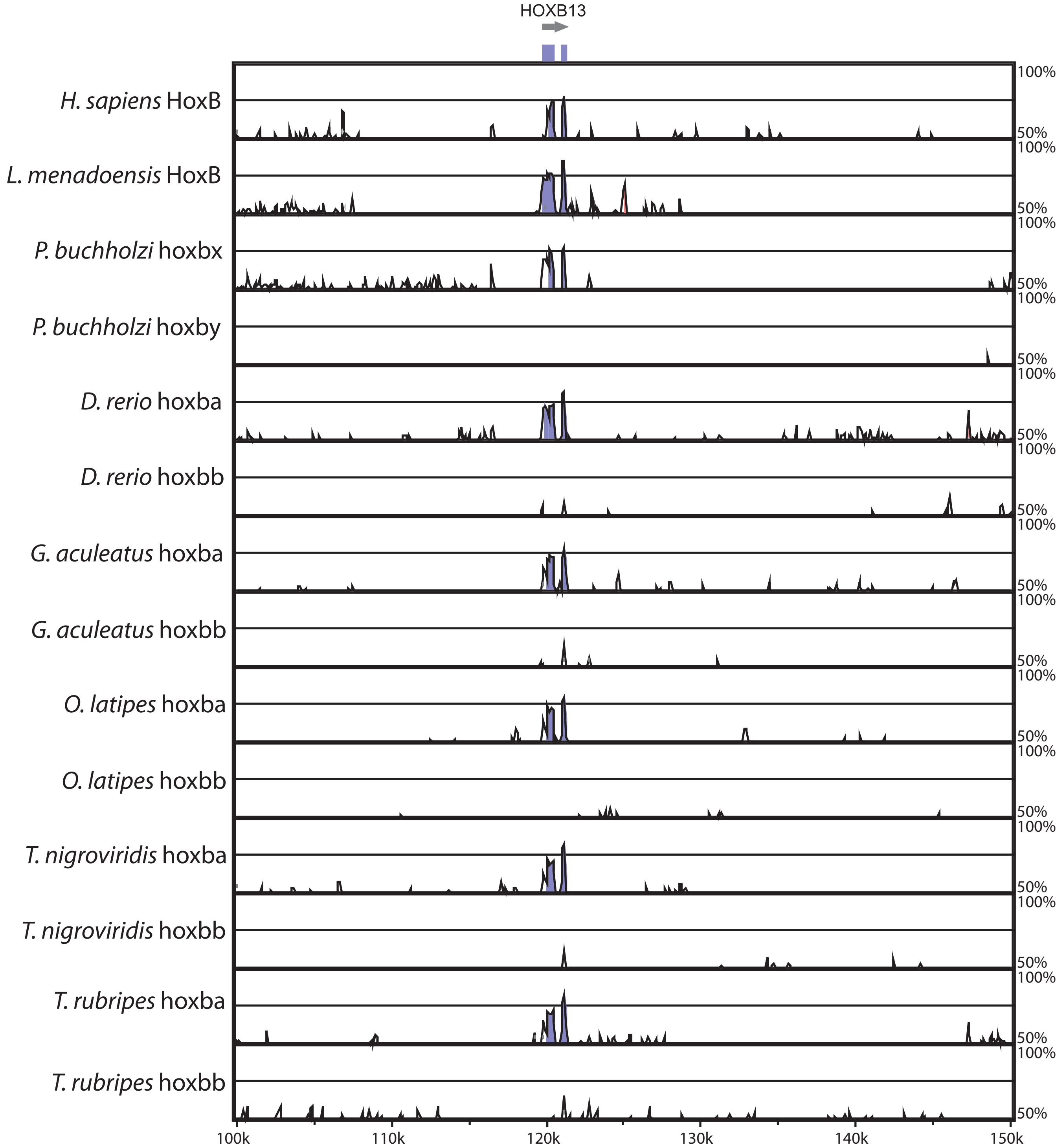

Cmi_HoxB |FJ824599.1|1-368325

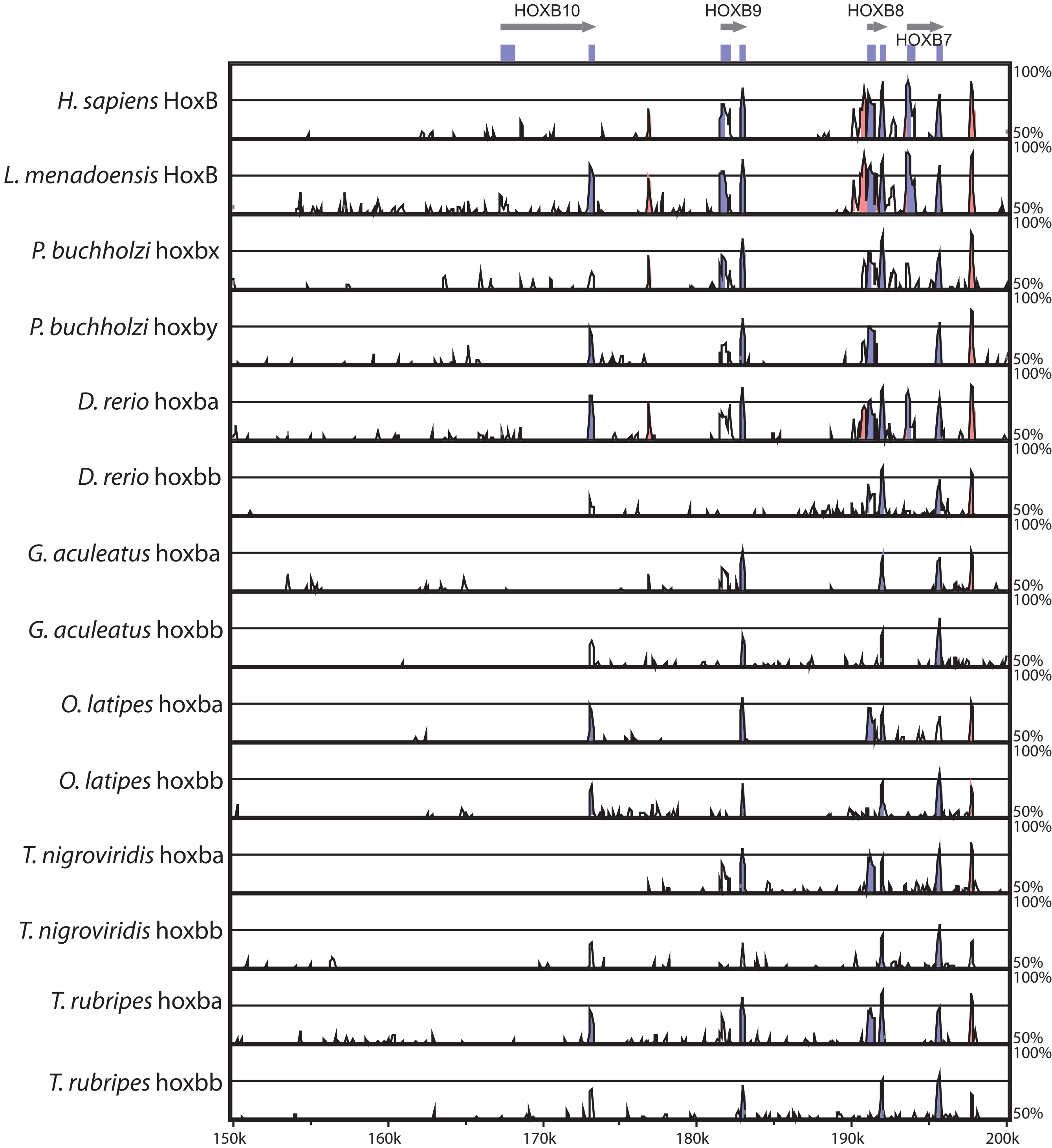

Cmi_HoxB|FJ824599.1|1-368325

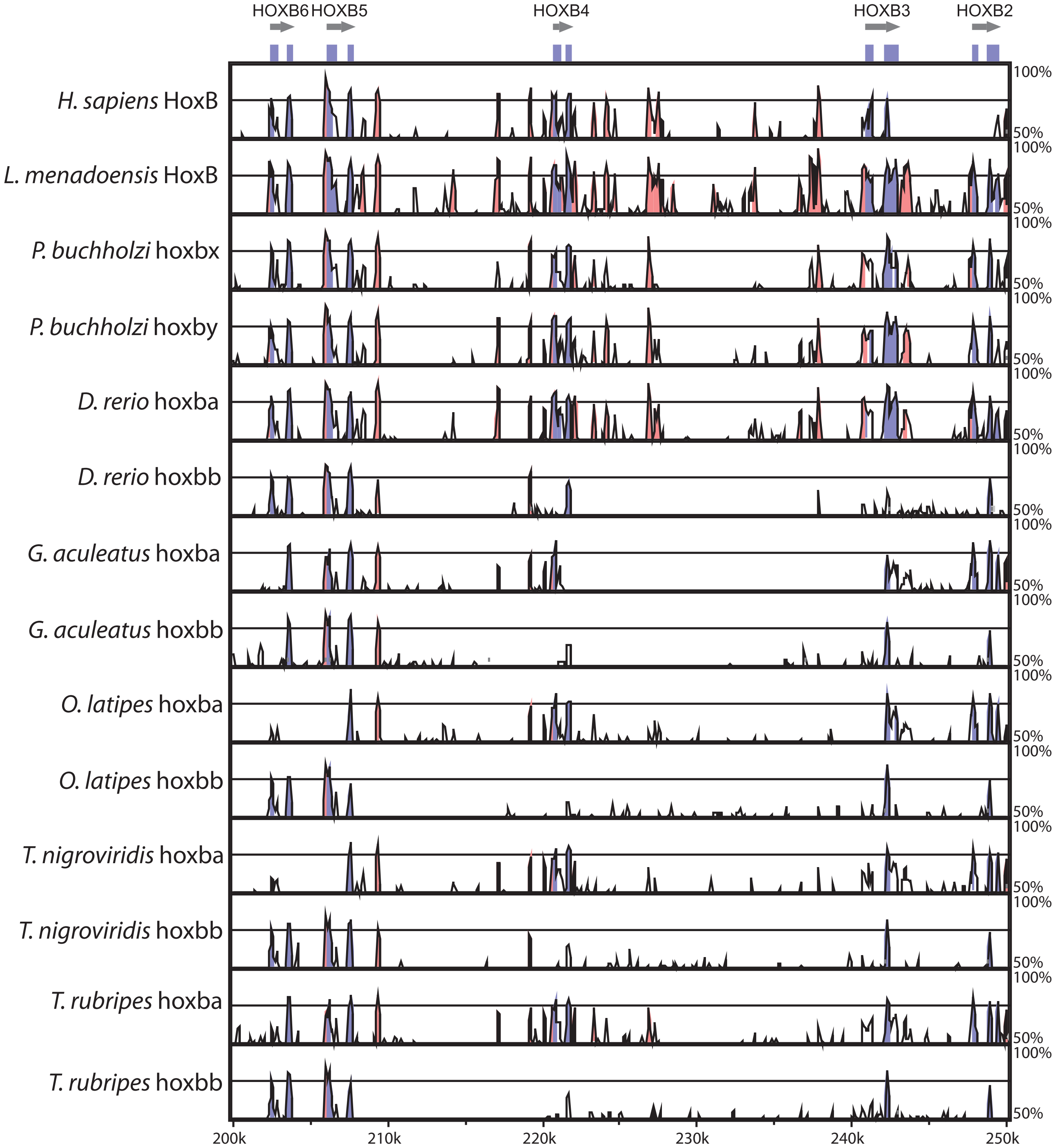

Cmi_HoxB|FJ824599.1|1-368325

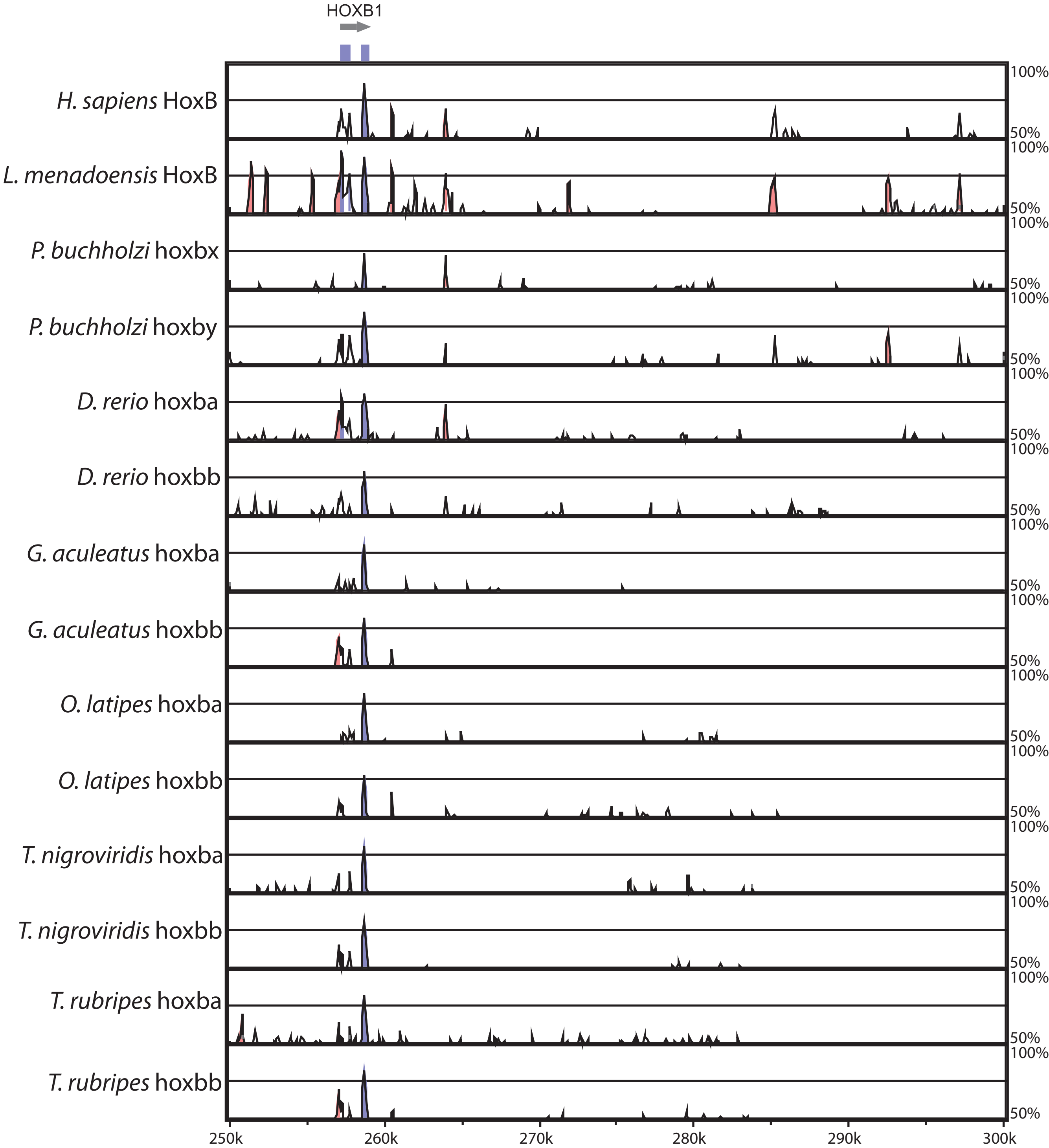

Cmi_HoxC |FJ824600.1|1-274370

HoxC VISTA alignment with *C. milli* baseline

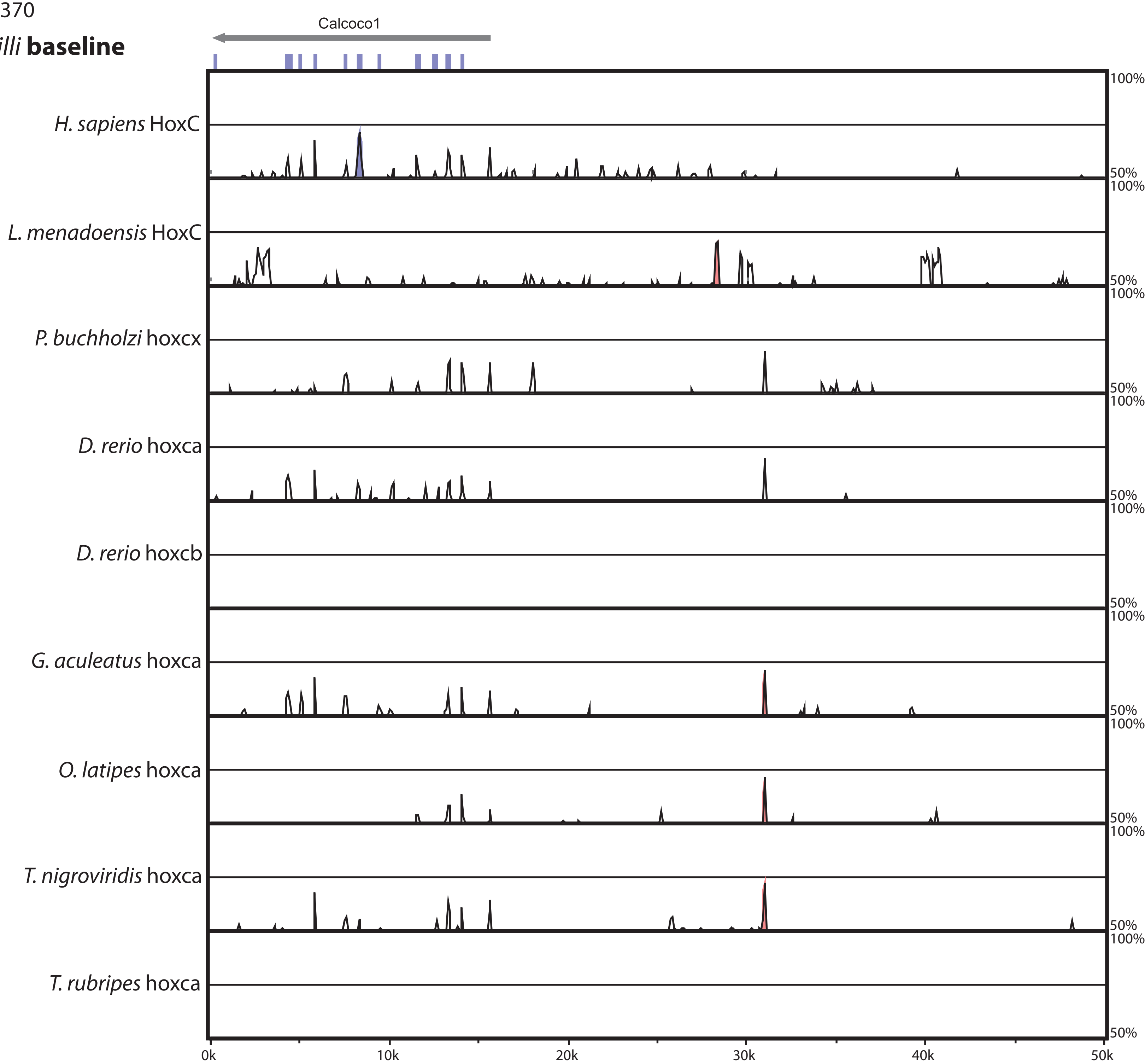

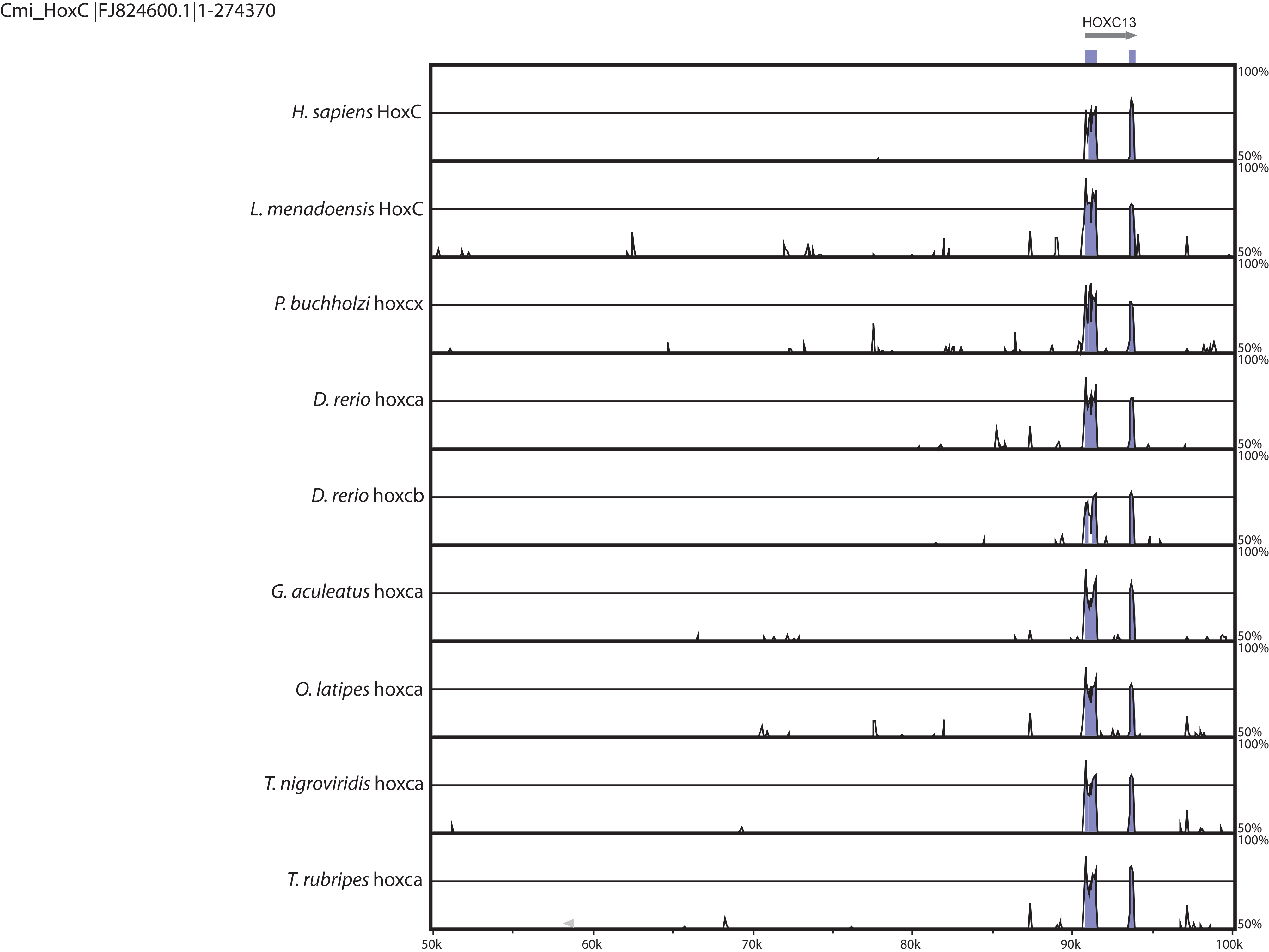
Cmi_HoxC |FJ824600.1|1-274370
HOXC13
H. sapiens HoxC
L. menadoensis HoxC
P. buchholzi hoxcx
D. rerio hoxca
D. rerio hoxcb
G. aculeatus hoxca
O. latipes hoxca
T. nigroviridis hoxca
T. rubripes hoxca
100%
50%
50k
60k
70k
80k
90k
100k

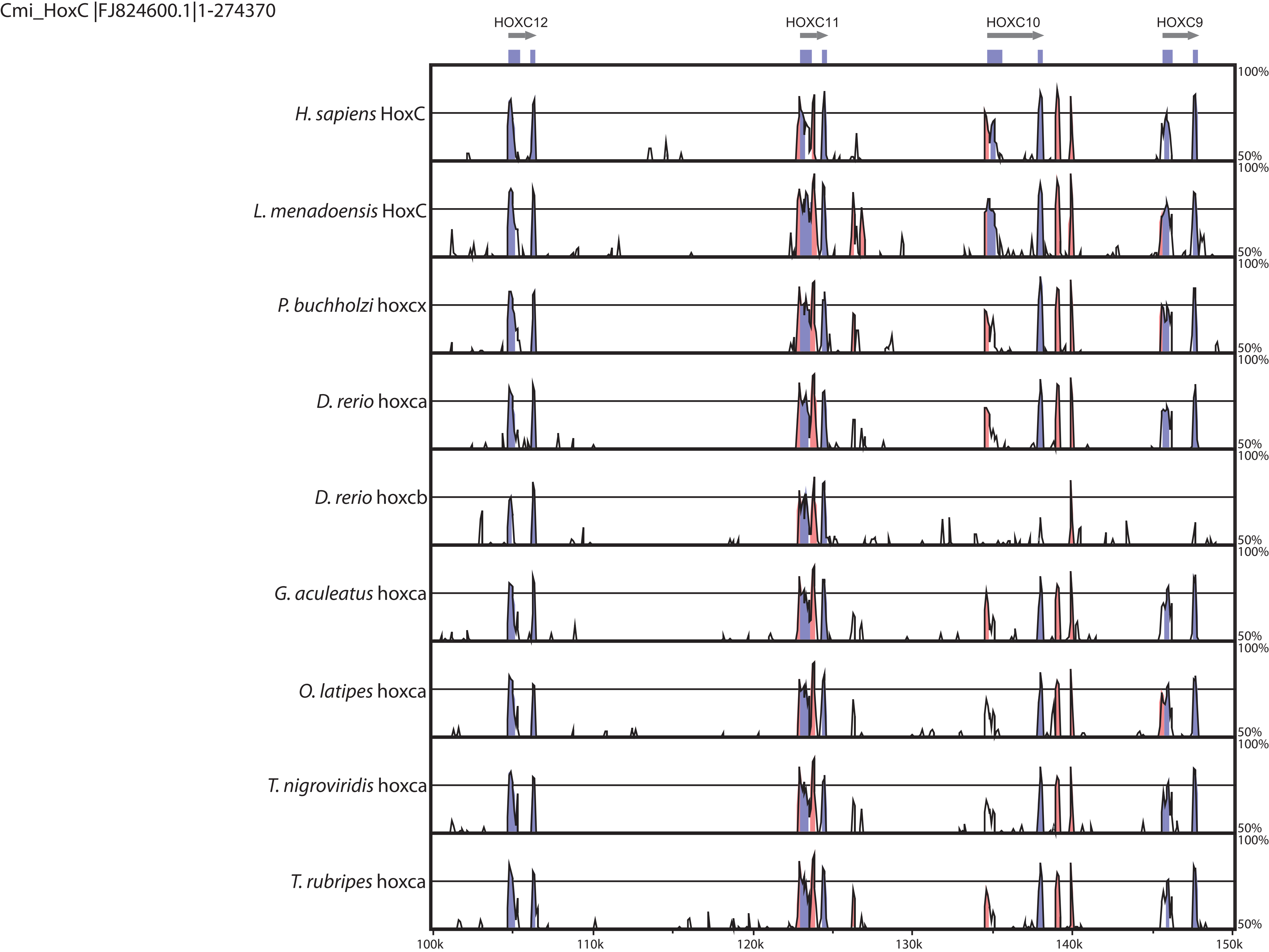

Cmi_HoxC |FJ824600.1|1-274370
HOXC12
HOXC11
HOXC10
HOXC9
H. sapiens HoxC
L. menadoensis HoxC
P. buchholzi hoxcx
D. rerio hoxca
D. rerio hoxcb
G. aculeatus hoxca
O. latipes hoxca
T. nigroviridis hoxca
T. rubripes hoxca
100%
50%
100k
110k
120k
130k
140k
150k

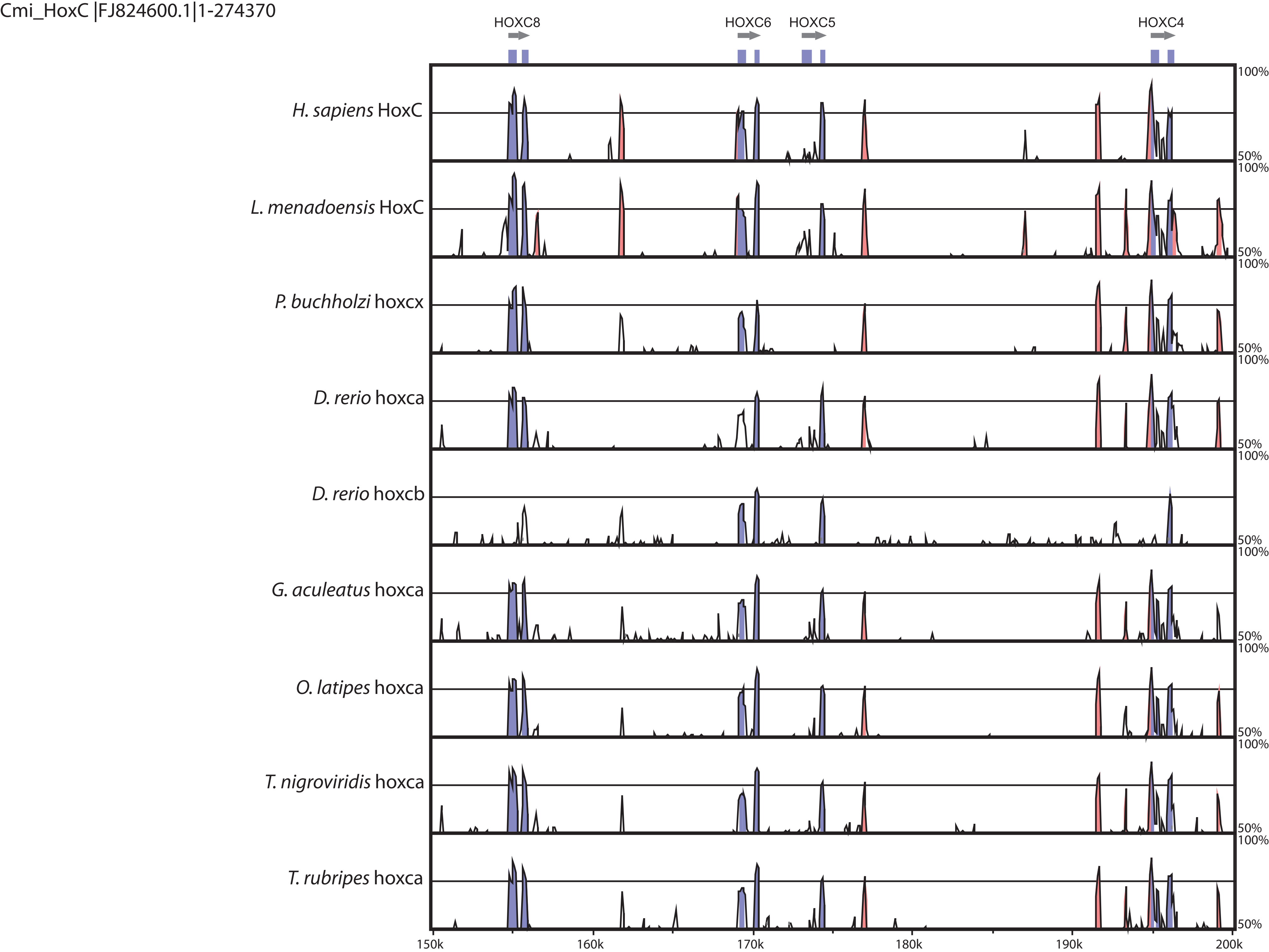
Cmi_HoxC |FJ824600.1|1-274370
HOXC8
HOXC6
HOXC5
HOXC4
H. sapiens HoxC
L. menadoensis HoxC
P. buchholzi hoxcx
D. rerio hoxca
D. rerio hoxcb
G. aculeatus hoxca
O. latipes hoxca
T. nigroviridis hoxca
T. rubripes hoxca
100%
50%
150k
160k
170k
180k
190k
200k

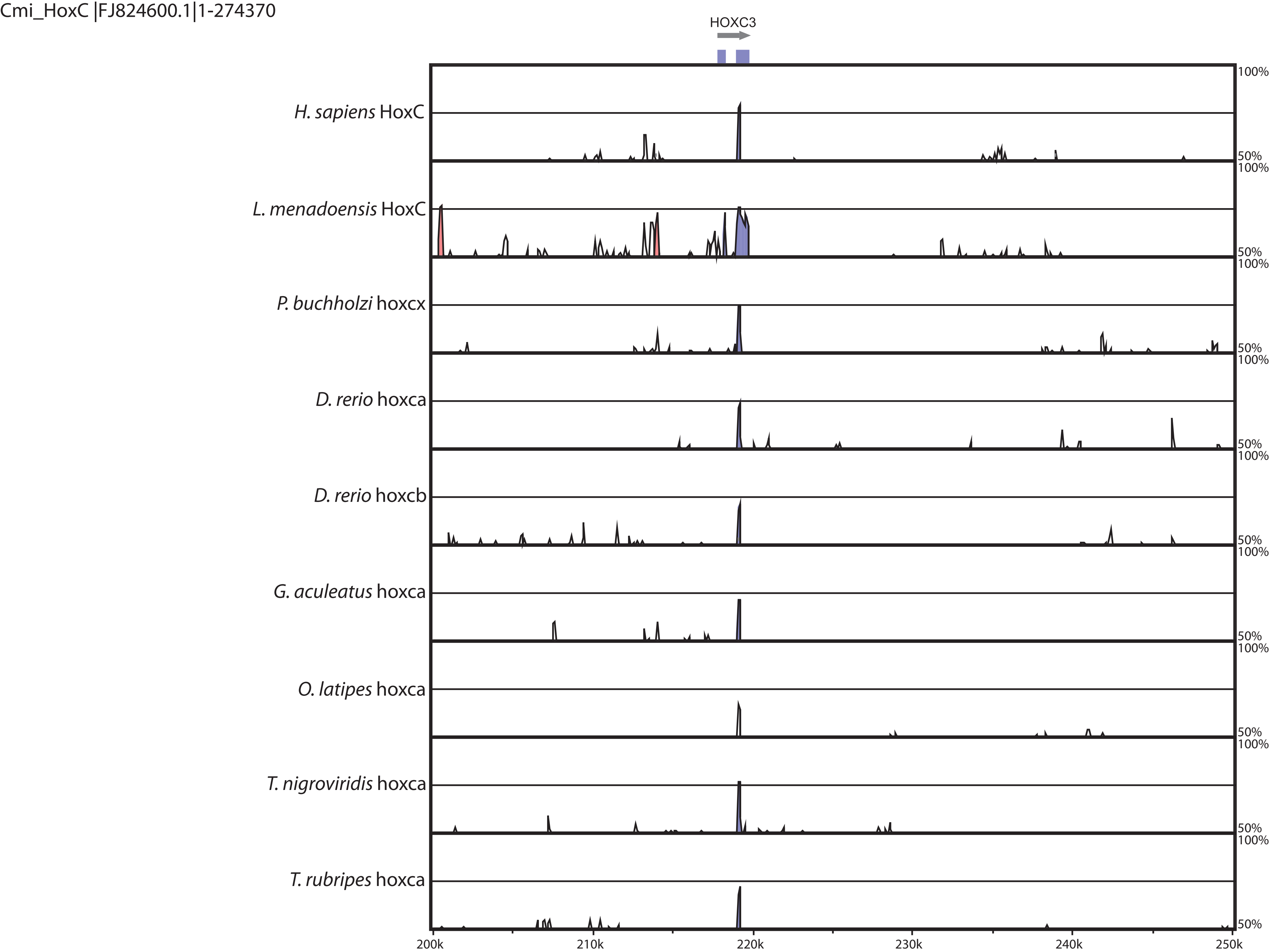

Cmi_HoxC |FJ824600.1|1-274370
HOXC3
H. sapiens HoxC
L. menadoensis HoxC
P. buchholzi hoxcx
D. rerio hoxca
D. rerio hoxcb
G. aculeatus hoxca
O. latipes hoxca
T. nigroviridis hoxca
T. rubripes hoxca
100%
50%
200k
210k
220k
230k
240k
250k

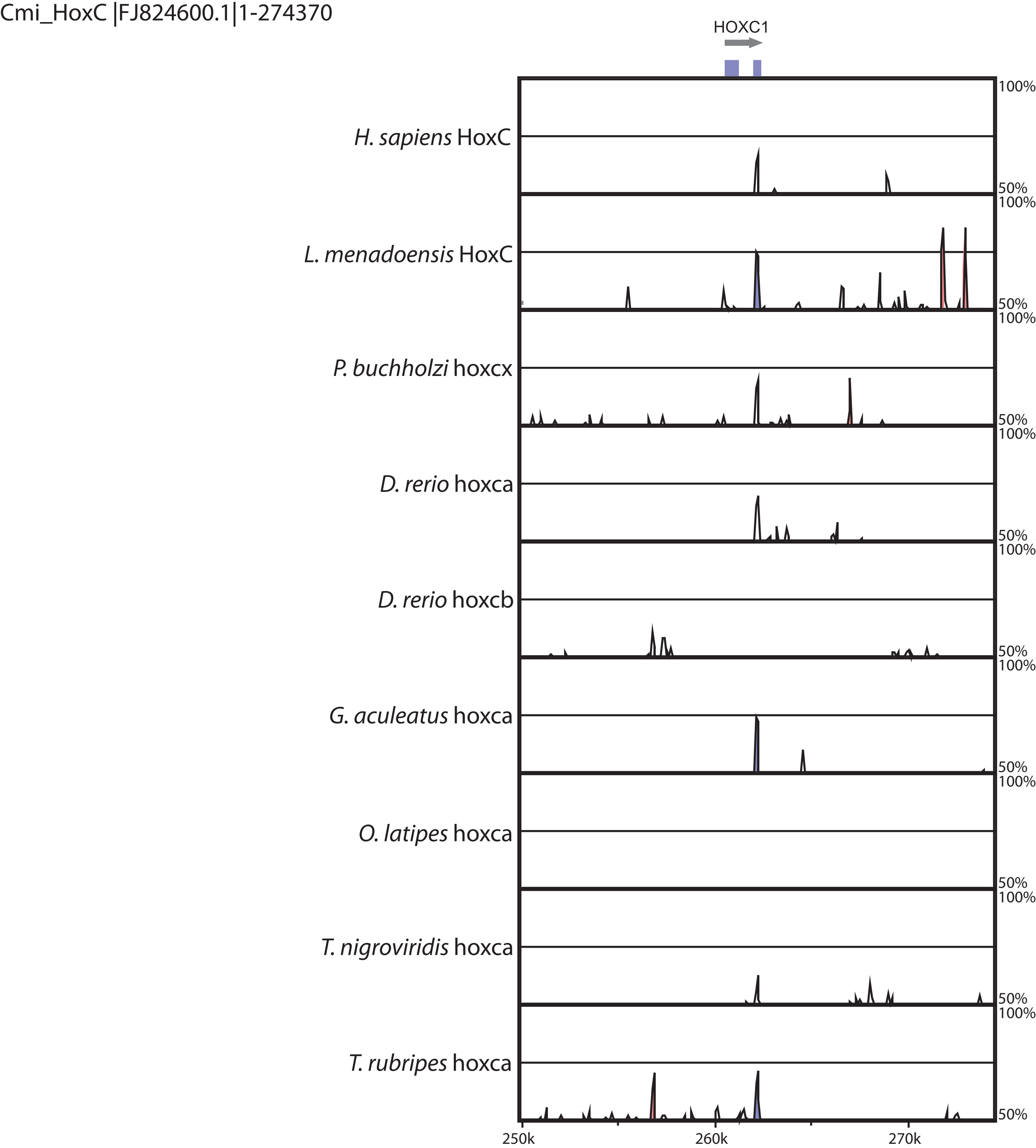
Cmi_HoxC |FJ824600.1|1-274370
HOXC1
H. sapiens HoxC
L. menadoensis HoxC
P. buchholzi hoxcx
D. rerio hoxca
D. rerio hoxcb
G. aculeatus hoxca
O. latipes hoxca
T. nigroviridis hoxca
T. rubripes hoxca
100%
50%
250k
260k
270k

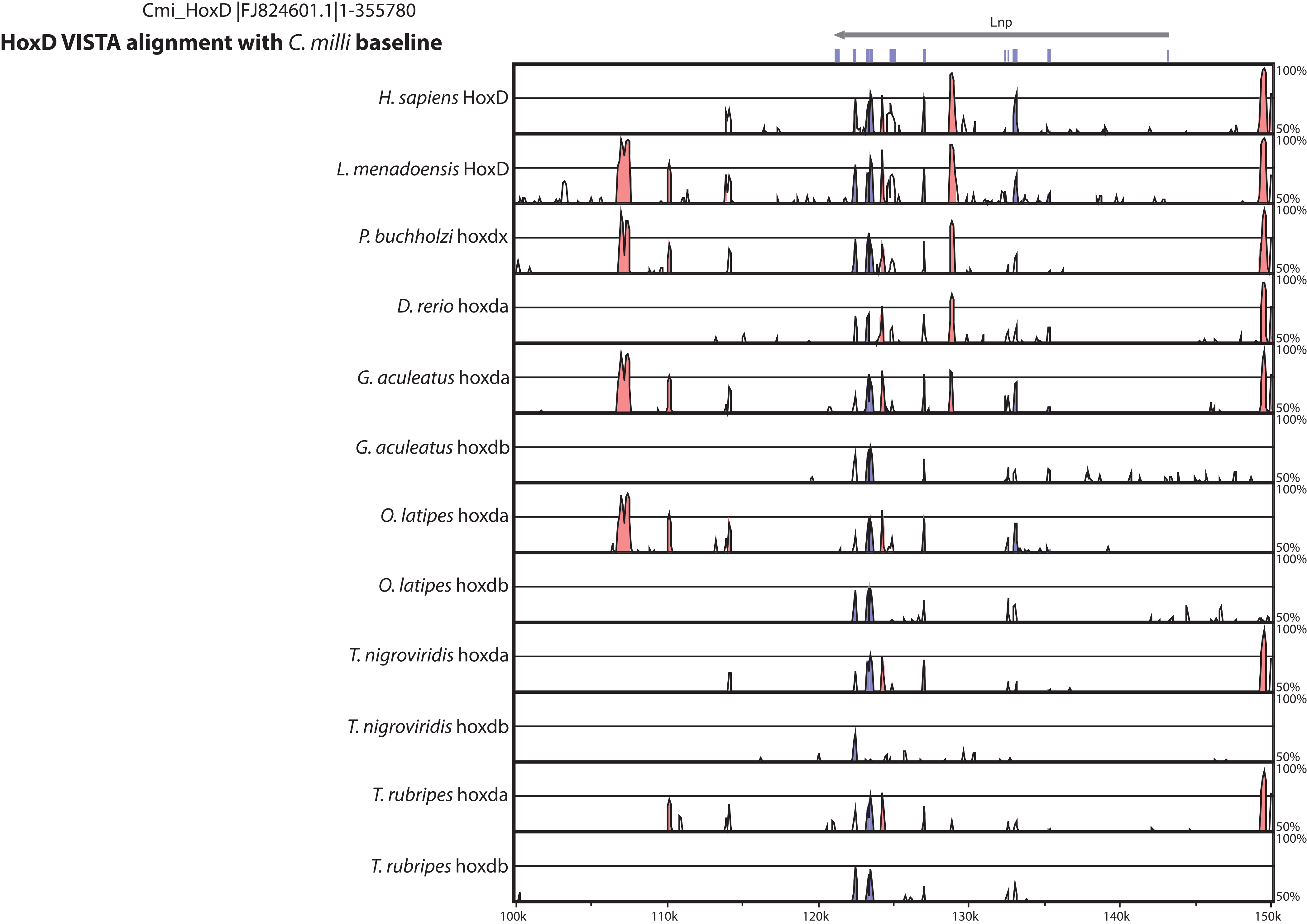
Cmi_HoxD |FJ824601.1|1-355780
HoxD VISTA alignment with C. milli baseline
Lnp
H. sapiens HoxD
L. menadoensis HoxD
P. buchholzi hoxdx
D. rerio hoxda
G. aculeatus hoxda
G. aculeatus hoxdb
O. latipes hoxda
O. latipes hoxdb
T. nigroviridis hoxda
T. nigroviridis hoxdb
T. rubripes hoxda
T. rubripes hoxdb
100%
50%
100k
110k
120k
130k
140k
150k

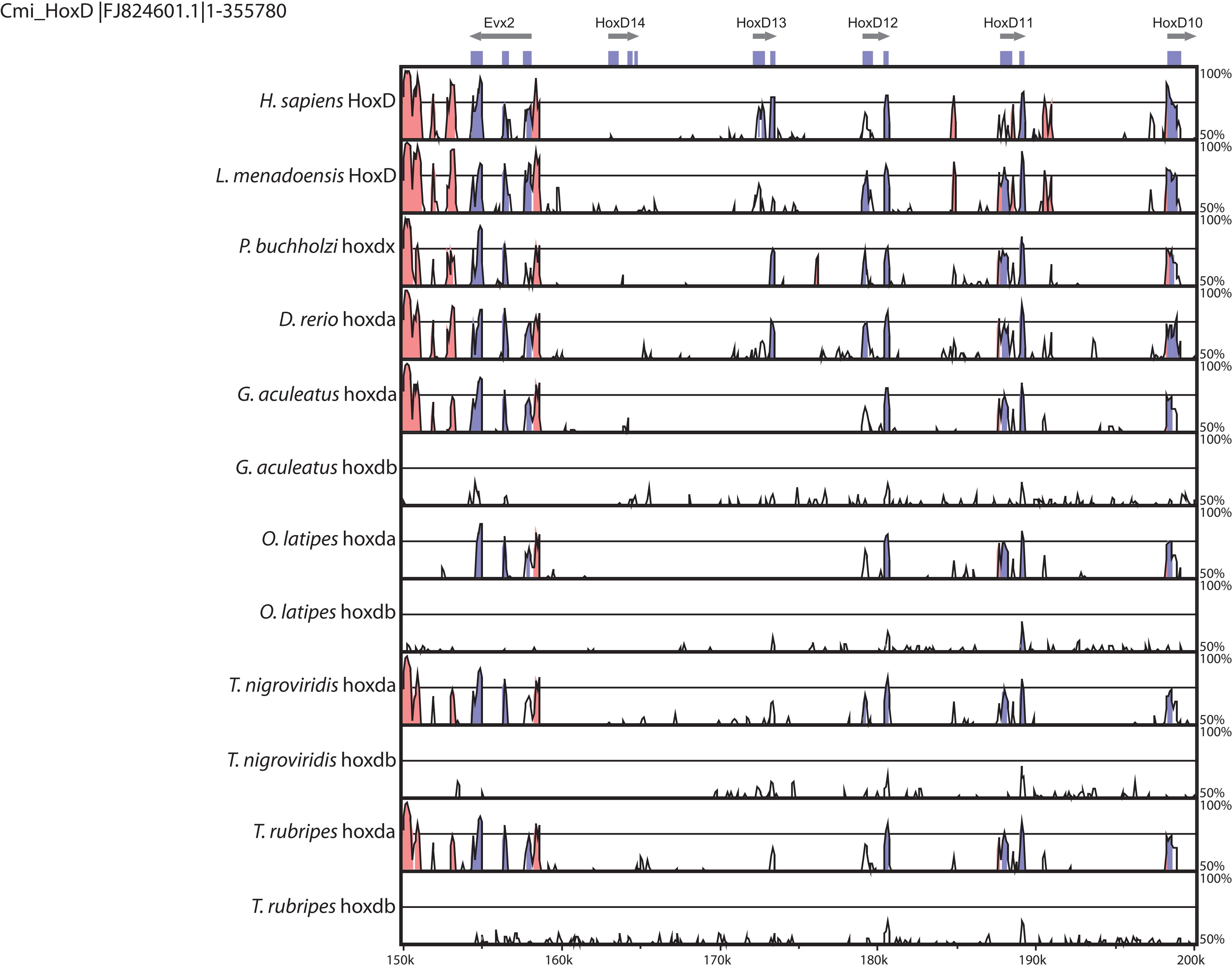

Cmi_HoxD |FJ824601.1|1-355780
Evx2
HoxD14
HoxD13
HoxD12
HoxD11
HoxD10
H. sapiens HoxD
L. menadoensis HoxD
P. buchholzi hoxdx
D. rerio hoxda
G. aculeatus hoxda
G. aculeatus hoxdb
O. latipes hoxda
O. latipes hoxdb
T. nigroviridis hoxda
T. nigroviridis hoxdb
T. rubripes hoxda
T. rubripes hoxdb
100%
50%
150k
160k
170k
180k
190k
200k

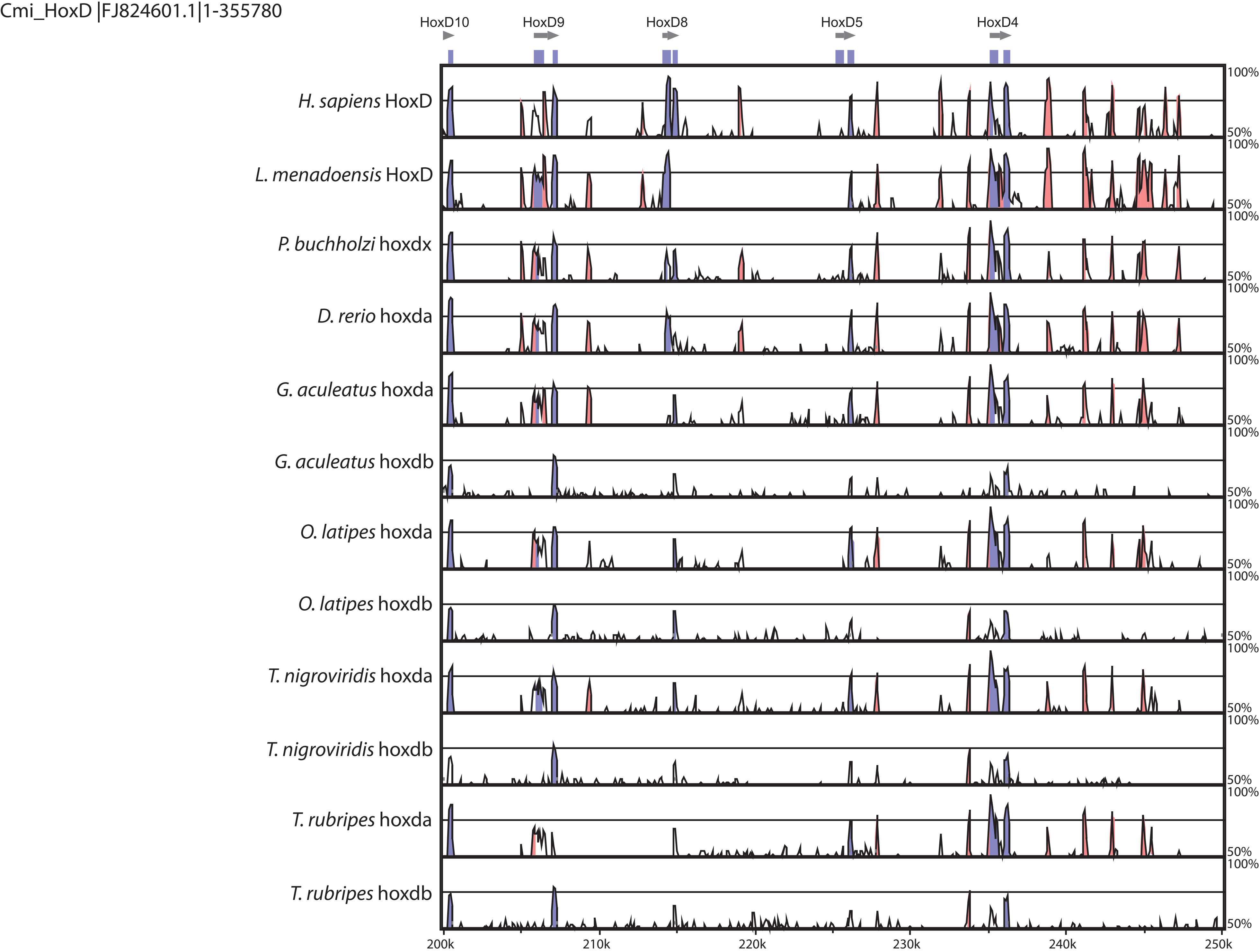

Cmi_HoxD |FJ824601.1|1-355780
HoxD10
HoxD9
HoxD8
HoxD5
HoxD4
H. sapiens HoxD
L. menadoensis HoxD
P. buchholzi hoxdx
D. rerio hoxda
G. aculeatus hoxda
G. aculeatus hoxdb
O. latipes hoxda
O. latipes hoxdb
T. nigroviridis hoxda
T. nigroviridis hoxdb
T. rubripes hoxda
T. rubripes hoxdb
100%
50%
200k
210k
220k
230k
240k
250k

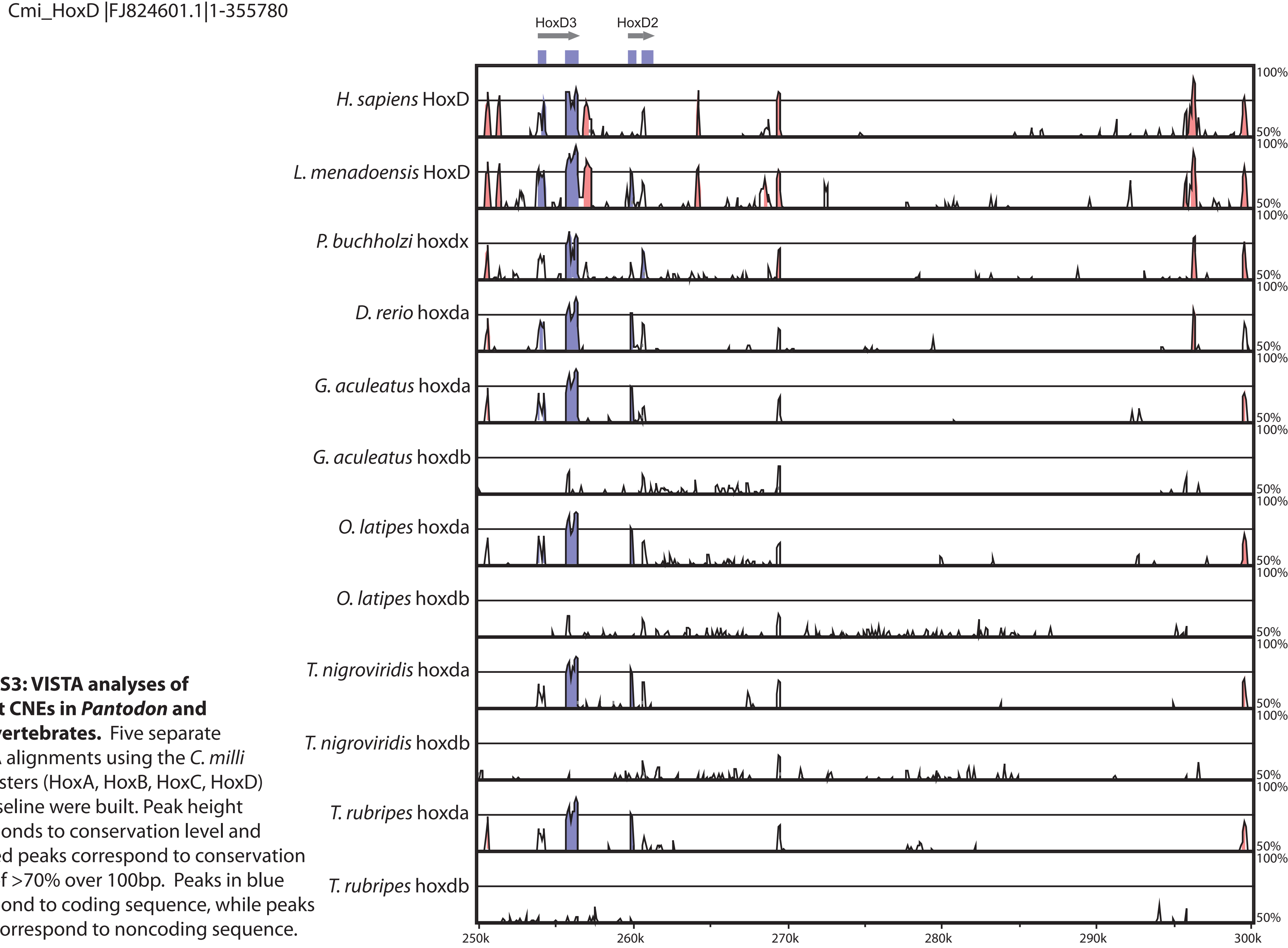

Figure S3: VISTA analyses of ancient CNEs in *Pantodon* and other vertebrates. Five separate mVISTA alignments using the *C. milli* hox clusters (HoxA, HoxB, HoxC, HoxD) as a baseline were built. Peak height corresponds to conservation level and coloured peaks correspond to conservation levels of >70% over 100bp. Peaks in blue correspond to coding sequence, while peaks in red correspond to noncoding sequence.

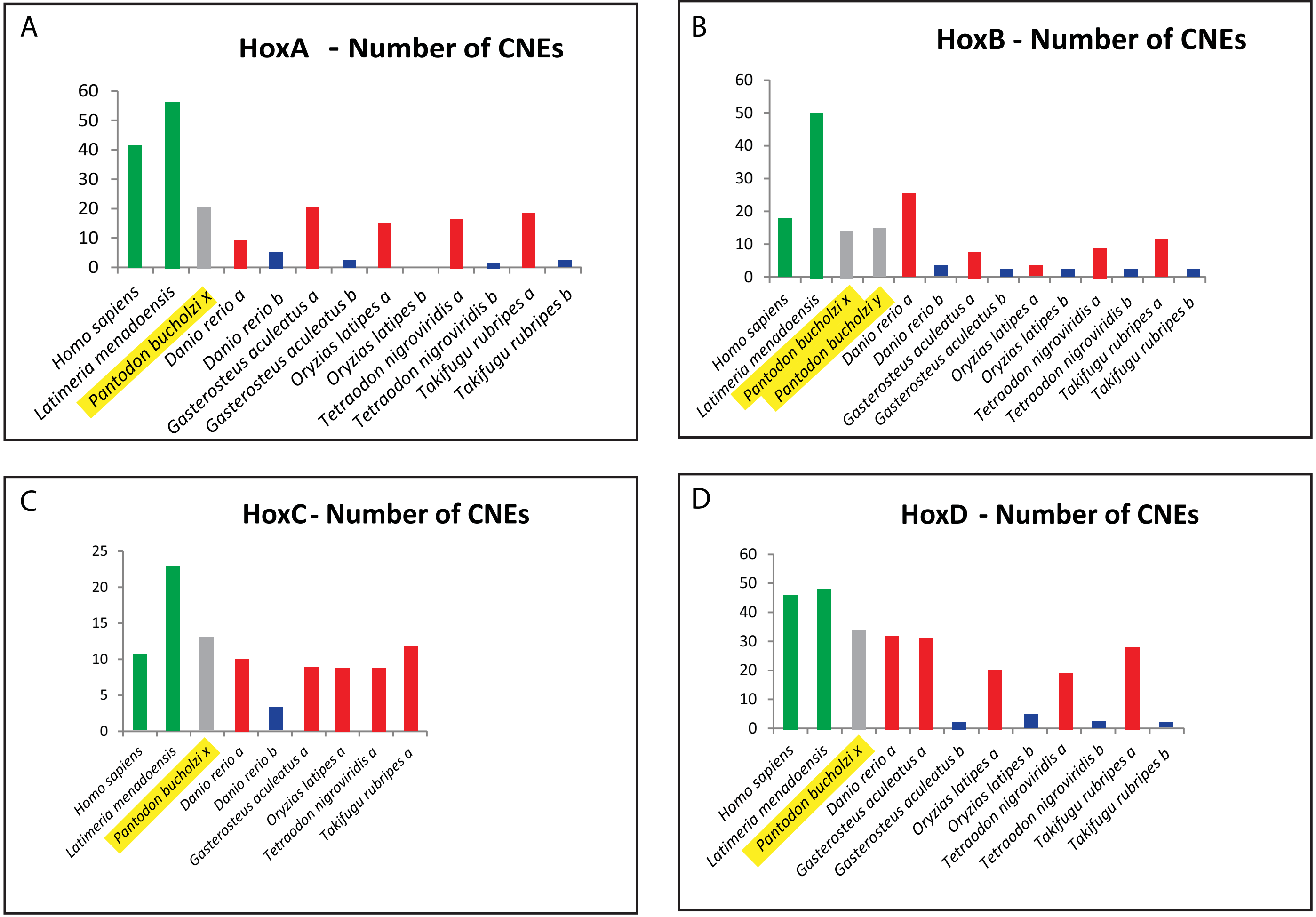

Figure S4: Ancient CNE conservation. Histograms showing the number of CNEs identified by multi VISTA alignment using *Callorhinchus milli* as the baseline sequence. The mLAGAN algorithm and a cutoff of 70% identity over 100bp was used. Columns corresponding to pre-TGD sarcopterygian Hox cluster CNE counts are represented in green, the post-TGD clupeocephalan 'a' cluster counts are in red, and the post-TGD clupeocephalan 'b' clusters are in blue. *Pantodon* Hox cluster CNE counts are coloured in gray. While extreme asymmetry in the retention of ancient CNEs between the post-TGD 'a' and 'b' clusters of other teleosts is evident, the hoxbx and hoxby clusters of *Pantodon* retain nearly equal numbers of ancient CNEs (B).
